# Abstracts of the 39th Annual CAPO Conference—Building Hope: Integrating Sustainable, Innovative and Accessible Care in Psychosocial Oncology 6 to 7 June 2024

**DOI:** 10.3390/curroncol31060257

**Published:** 2024-06-16

**Authors:** Peter Traversa, Doris Howell

**Affiliations:** 1 Canadian Association of Psychosocial Oncology, Toronto, ON M5A 1S2, Canada; 2 Princess Margaret Cancer Research Institute, University of Toronto, Toronto, ON M5G 2M9, Canada

**Keywords:** psychosocial, oncology, cancer, research, accessible, sustainable, innovation

## Abstract

On behalf of the Canadian Association of Psychosocial Oncology, we are pleased to present the Abstracts from the 2024 Annual Conference, titled “Building Hope: Integrating Sustainable, Innovative and Accessible Care in Psychosocial Oncology”. The Conference was held in Calgary from 6 June 2024 to 7 June 2024. This conference brought together key stakeholders including multidisciplinary professionals from nursing, psychology, psychiatry, social work, spiritual care, nutrition, medicine, rehabilitation medicine, occupational health and radiation therapy for both adult and pediatric populations. Participants included clinicians, researchers, educators in cancer care, community-based organizations and patient representatives. Patients, caregivers and family members presented abstracts that speak to their role in managing cancer experiences and care. Over one hundred (150) abstracts were selected for presentation as symposia, 20 min oral presentations, 10 min oral presentations, 90 min workshops and poster presentations. We congratulate all the presenters on their research work and contribution.

## 1. Abstract Themes

- Adapting PSO care in LMI countries.
- Cancer treatment-related symptom and toxicity management.
- Cancer care across the life span (children, adolescent and young adults, adults and older adults).
- Community-based and volunteer cancer care services.
- Complementary and integrative cancer care.
- Digital health and cancer care.
- Exercise/pre-habilitation and rehabilitation in cancer.
- Equity, diversity and inclusion in cancer care and research.
- Healthcare provider wellness.
- Implementation science, knowledge translation and synthesis.
- Innovation in psychosocial oncology interventions.
- Palliative and end-of-life care.
- Pandemics and cancer care issues.
- Patient-oriented research approaches.
- Primary, secondary and tertiary cancer prevention.
- Sociodemographic, culture and sex/gender issues in cancer.
- Survivorship.
- Other value-based and person-centered cancer care.

## 2. Symposia

### 2.1. S1 Interventions to Improve Transition from Pediatric to Adult Care for Youth Diagnosed with Cancer

**Moderator**

Leandra Desjardins, Nathalie Labonté.

**Summary**

There is a growing population of pediatric cancer survivors who are facing a shift in focus from acute treatment to long-term care follow-up. The transition from pediatric to adult healthcare services requires increased autonomy in several areas including: disease self management, interpersonal assertiveness and navigating academic and employment resources. Families report feeling unprepared to face these new challenges.

This symposium has two overarching aims:

(1) We will share three different research-based intervention approaches to support transition readiness in pediatric cancer survivors: screening transition skills in clinical care, transition preparation workshops for youth and caregivers and the development of an academic and vocational navigation program.

(2) The symposium will be co-moderated by Nathalie Labonté, a parent of a young adult who was diagnosed with a brain tumor as a child, who also holds a master’s degree in research (subject: “My student has cancer: How to intervene?”), and collaborated at various levels on all three projects described above. She will share her experience as a parent of a young adult transferring to adult care and as a partner in research on this topic.

We hope to foster engagement in the important topic of transition preparation for pediatric cancer survivors.

**S1-24**

#### 2.1.1. Targeted Transition Readiness Workshop Intervention for Pediatric Brain Tumor Survivors (PBTS)

Leandra Desjardins, Marco Bonanno, Julie Carrier, Nathalie Labonté, Carole Provost, Tziona Lugasi, Andrea Saragosti, Benedicte Koukoui, Geneviève Brault and Serge Sultan.

**Background/rationale or objectives/purpose**

PBTS are at risk of physical, cognitive and psychosocial difficulties which may impact the development of autonomy skills necessary to navigate adult healthcare (transition readiness skills). However, existing transition interventions often focus solely on disease management. This presentation aims to describe the first transition intervention workshops targeting the specific needs of PBTS and their caregivers.

**Methodology or Methods**

**Sample and setting**: this study used a mixed method to evaluate three 1 ½ h hybrid-format workshops for dyads of PBTS (ages +14 years) and their caregivers (N = 11 dyads).

**Procedures**: Each workshop covered a specific topic (disease management, social competence and cognitive functioning) and followed the same structure (topic presentation, person with lived experience sharing, teaching two strategies). Feasibility and satisfaction were assessed via recruitment, retention, a satisfaction survey and brief focus groups.

**Results**: The dyads retention rate was 65%. Overall, the participants reported the workshops as useful (parents = 86%; PBTS = 73%). Parents were the least satisfied with the practical activities and PBTS with the duration of the workshops. The dyads most appreciated hearing from others with lived experience.

**Impact on practice or results**

There is a need to focus on transition readiness skills beyond disease self-management and to consider other contextual factors (social relationships and skills, cognitive and academic functioning) impacting autonomy.

**Discussion or Conclusions**

There is the potential for a transdiagnostic application of these workshops, given that other pediatric populations experience similar challenges. We are now exploring this next step in our clinical research program.

**S1-72**

#### 2.1.2. Preparing for the Implementation of Transition Readiness Assessment: A Qualitative Study of Pediatric Cancer Survivor and Parent Experiences and Preferences

Marika Monarque, Nadege Gendron Granger, Buron Laurianne, Carole Provost, Zeev Rosberger, Tsimicalis Argerie, Nathalie Labonté, Élodie Bergeron, Marco Bonanno and Leandra Desjardins.

**Background/rationale or objectives/purpose**

A crucial element for the long-term well-being of pediatric cancer survivors is preparing for the transition from pediatric to adult healthcare. Guided by an *implementation science approach*, this project seeks to integrate transition readiness screening in the routine care of survivors, using the validated *Transition Readiness Assessment Questionnaire* (TRAQ). This study assessed pediatric cancer survivor and parent experiences with transition preparation and their preferences for screening and resources.

**Methodology or Methods**

Focus groups were conducted with 14–18-year-old pediatric cancer survivors (n = 14) and parents (n = 5). Focus groups assessed perceptions of care at their long-term follow-up clinic, transition preparedness, preferences for TRAQ administration (e.g., format, moment, location) and preferences for transition readiness resources. Interviews were analyzed with an inductive thematic analysis approach.

**Impact on practice or results**

The participants suggested leveraging technology (e.g., QR codes, online administration) for screening and transition resource delivery. They preferred receiving the TRAQ in the waiting room during annual appointments and suggested modifying certain items for clarity. The youth reported being unaware of what transition entailed and parental distress was described regarding long-term follow up care and transition preparation. Individualized support and clinician discussions regarding transition preparation and screening were suggested to address barriers.

**Discussion or Conclusions**

This first step in an implementation science approach is essential to align TRAQ implementation with the needs of families. The next steps involve documenting implementation processes and outcomes on transition readiness. This project will inform the creation of a screening implementation manual to improve transition care in other sites in Canada, with far-reaching implications for youth with chronic conditions.

**S1-7**

#### 2.1.3. Development of a Transition Liaison Program Improving School and Work Services for AYA Pediatric Brain Tumor Survivors (PBTS)

Marco Bonanno ^1,2,3^, Leandra Desjardins ^1,2,4^, Claude Julie Bourque ^5^, Lye-Ann Robichaud ^3,6^, Nathalie Labonté ^4^, Carole Provost ^1^, Marie-Claude Charrette ^1^, Michel Duval ^1,2,5^ and Serge Sultan ^2,3,6^.

^1^  CHU Sainte-Justine, Montréal, Canada.
^2^  CHU Sainte-Justine Research Center, Montréal, Canada.
^3^  Psycho-Oncology Center (CPO), Montréal, Canada.
^4^  Pediatric Oncology Neuro Social Lab (PONS), Montréal, Canada.
^5^  Department of Pediatrics of Université de Montréal, Montréal, Canada.
^6^  Department of Psychology of Université de Montréal, Montréal, Canada.

**Background/rationale or objectives/purpose**

PBTS may experience academic and employment difficulties during aftercare. However, there are only a few evidence-based structured programs which provide long-term support to ensure and monitor their school re-entry and labor market integration. This presentation aims to describe the steps leading to the development of a medical-school–employment liaison program tailored for PBTS.

**Methodology or Methods**

We organized two online workshops with a convenience sample of professionals (N = 15) and decision makers (N = 6) from different sectors (health, education, community organizations) based on the results of an online survey from PBTS and parents (N = 68). The workshop with professionals aimed at identifying the main characteristics that would define the liaison team. The workshop with decision makers aimed at identifying institutional strategies for promoting the implementation of an intersectoral liaison program (2 h each). Data analysis was performed via deductive content analysis.

**Impact on practice or results**

The participants highlighted three main axes to be developed in the future liaison program: awareness-raising actions regarding the needs of PBTS, the long-term evaluation of PBTS (re)integration needs and follow-up strategies and roles and responsibilities of its members. The participants formulated recommendations on the mandate of the liaison team: enabling liaison between hospitals and rehabilitation centers with access to work programs and counseling, coordinating the integration between secondary and post-secondary education levels, providing guidance and advising survivors throughout the different stages of life and providing equitable access to resources and services.

**Discussion or Conclusions**

The next step will focus on the design and pilot testing for the implementation of the medical-school–work transition liaison program.

### 2.2. S3 Changing the Approach of Psychosocial Oncology Care to Address Unmet Needs in Regional, Rural and Remote Settings

**Moderator**

Anna Singleton, Rachel MacNeil and Julia Kaal.

**Summary**

People living with and beyond cancer in regional, rural and remote communities are under-represented in psycho-oncology research and experience significant unmet healthcare needs. Novel approaches for the development and implementation of psycho-oncology research and clinical care are critical for addressing these needs. In this symposium, speakers will present various strategies to equitable and accessible psycho-oncology research and care for people living in these communities. First, A. Singleton (Sydney University, Australia) and R. Strong (NWT Breast Health/Breast Cancer Action Group) will co-present an overview of psycho-oncology research and care in Australia and Canada. They will also showcase how they co-designed a community-led digital health program that will deliver support and connections to psycho-oncology care in Northwest Territories Canada. Second, R. MacNeil from the Hotıì Ts’eeda NWT SPOR Unit will share their experiences with supporting psycho-oncology research and care for Indigenous peoples in NWT. Third, KJ. Kaal and M. Arab from the Nova Scotia Health Cancer Care program will showcase the implementation and impact of and patient satisfaction with community-based psycho-oncology care in rural Nova Scotia. The barriers and enablers to each strategy will be discussed and implications for optimizing the accessibility of future research and clinical care in regional, rural and remote settings.

**S3-29**

#### 2.2.1. Co-Designing the TextMeHealthy Program to Support Women in Northwest Territories (NWT) Canada; a Community-Based Volunteer-Led Collaboration with Australian Researchers

Anna C Singleton ^1^, Allyson Todd ^2^, Rebecca Raeside ^2^, Karice K Hyun ^1,3^, Rosanna Strong ^4^, Jill Christensen ^4^, The NWT Breast Health/Breast Cancer Action Group Board members ^4^, TextMeHealthy Project Advisory Committee ^4,5,6^, Stephanie R Partridge ^2^ and Julie Redfern ^1^.

^1^  Engagement and Co-design Research Hub, The University of Sydney, Sydney, Australia.
^2^  School of Nursing, The University of Sydney, Sydney, Australia.
^3^  Concord Repatriation Hospital, Sydney, Australia.
^4^  The NWT Breast Health/Breast Cancer Action Group, Yellowknife, Canada.
^5^  University of Alberta, Edmonton, Canada.
^6^  Hotıì Ts’eeda NWT SPOR Unit, Yellowknife, Canada.

**Background/rationale or objectives/purpose**

NWT has limited access to psycho-oncology care and research. Community-based organizations, including the NWT Breast Health/Breast Cancer ‘Action Group’, deliver accessible resources. However, research collaborations with NWT community-based organizations are limited. We aimed to co-design a digital health psycho-oncology support program through a collaboration between the Action Group and Sydney University (Australia). The enablers and barriers are discussed.

**Methodology or Methods**

The iterative mixed-methods co-design process of a psycho-oncology-focused text message intervention, including focus groups, surveys to rate message acceptability, usefulness and appropriateness (5-point Likert Scales; score/15) and end-user consensus. Eligibility: NWT health professionals, researchers, community members or women diagnosed with breast, lung, ovarian, cervical or colorectal cancer. Recruitment: emails, phone calls and social media with e-consent. Transcripts and free-text data were parallel coded and analyzed using deductive thematic analysis.

**Impact on practice or results**

The participants (N = 41) had a mean age ± SD of 58 ± 11 years and identified as women (100%), Indigenous (12/41; 29%) and university/college graduates (38/41; 93%). This process resulted in 82 highly rated (scores ≥ 13.5/15), co-designed text messages, which form the ‘TextMeHealthy’ program (2–4 messages/week for 3 months). Message themes included mental health, self-management (e.g., exercise, side-effects), navigating rural healthcare and affordable nutrition. Subthemes included Indigenous health practices (cultural, spiritual) and web-access to resources. Collaboration enablers included passionate community members, open communication and strong community connections. Barriers: skepticism about ‘outsiders’, funding and time constraints.

**Discussion or Conclusions**

This community-led co-design process was feasible and resulted in highly rated, locally and culturally appropriate text messages. Enablers and barriers may facilitate future research–community–organization collaboration in remote communities. The delivery of TextMeHealthy in NWT will be evaluated 2024–2025.

**S3-42**

#### 2.2.2. Innovative Approaches to Supporting Grassroots Community Research in the Northwest Territories

Rachel MacNeill
Hotıì ts’eeda, Yellowknife, Canada.

**Background/rationale or Objectives/purpose**

Hotıì ts’eeda is a Canadian Institute of Health Research-funded SPOR SUPPORT Unit for the Northwest Territories. It is the first and only unit of its kind to be hosted in an Indigenous government, the Tłı̨chǫ Government and governed primarily by Indigenous governments. This innovative model allows the organization to use creative, responsive approaches to redefining research in the northern Indigenous context and support grassroots health and wellness projects that might otherwise fall through the gaps of traditional health and research funding.

**Methodology or Methods**

This presentation will describe Hotıì ts’eeda’s unique approach to defining and supporting community health and wellness research, rooted in Indigenous and northern values. Using the “Text Me Healthy” project as an example, it will identify the ways in which these values can be applied to varying contexts and make the case for expansion in the definition of health research.

**Impact on practice or results**

Hotıì ts’eeda aims to contribute to the broader conversation about health and wellness work in northern, rural and Indigenous contexts, pushing the boundaries on what is considered research and who gets to make decisions around what happens in these communities.

**Discussion or Conclusions**

With the growing emphasis on patient-oriented research, Hotıì ts’eeda hopes to strengthen and expand these important approaches to the benefit of communities across Canada.

**S3-66**

#### 2.2.3. Impact, Opportunities and Satisfaction with Psychosocial Oncology Care at a Rural Community-Based Oncology Center in Nova Scotia

Katrin Julia Kaal ^1,2^, Terrilee O’Connell ^1^, Lisa D’Eon ^1^, Pam Robichaud ^1^, Jennifer Boone ^1^, Camryn Salyzyn ^1^, Anna Nazarova ^1^, Marianne Arab ^1^ and Joy Tarasuk ^1^.

^1^  Nova Scotia Health, Cancer Care Program, Halifax, Canada.
^2^  Dalhousie University, Halifax, Canada.

**Background/rationale or objectives/purpose**

A rural community-based oncology center in Nova Scotia successfully implemented a new model of care to better meet patient needs relating to finances, travel and accessing comprehensive cancer care closer to home. Added resources included a full-time social worker and hematology-focused nurse practitioner. Utilizing an interdisciplinary, team-based approach to cancer care, the social worker and NP identified the specific needs of patients suffering from cancer-related fatigue (CRF), characterized by excessive and persistent exhaustion, as a gap in care. Their initiative employed an in-person group format to provide comprehensive education on CRF, stress management, sleep hygiene, exercise, nutrition, emotional health and inter-personal relationships.

**Methodology or Methods**

The group’s implementation was informed by a rapid review of best practices and gaps in comprehensive CRF programs. The initial cancer-related fatigue group focused on patients with hematologic malignancies and entailed four consecutive weekly sessions and a booster session. The participants completed the FACIT-F fatigue measure to assess baseline and post-group levels of fatigue and quality of life. Additionally, the content and format of the group were evaluated.

**Impact on practice or results**

The development of the group format allowed participants to better understand, accept and manage their fatigue and was associated with reduced fatigue and a better quality of life.

**Discussion or Conclusions**

A dedicated effort facilitated the success of a pilot CRF-specific patient group at a rural community oncology site. The second group is scheduled for April. Going forward it will be offered twice a year (spring and fall) at the pilot site and eventually scaled to seven additional community oncology sites in NS.

### 2.3. S4 Innovative Mind–Body Interventions in Oncology

**Moderator**

Linda Carlson.

**Summary**

Complementary and Integrative Medicine (CIM) therapies are commonly used by people living with cancer, with over 50% reporting the use of at least one CIM therapy since their cancer diagnoses. These therapies include evidence-based mind–body interventions such as mindfulness meditation and yoga, as well as more experimental modalities including psychedelic-assisted therapies. This series of talks from the Carlson Lab covers a range of interventions and delivery modalities of CIM therapies, applying different research methodologies from a large-scale randomized controlled trial to qualitative and survey methods, as well as biomarker analyses.

The first talk will describe the SEAMLESS Canada-wide randomized controlled trial, which is evaluating the efficacy of a mindfulness app based on the Mindfulness-Based Cancer Recovery (MBCR) program to manage stress and treatment-related side-effects. The second talk will describe the Mindful-Gut study, which is extending our understanding of the mind–body connection by evaluating the effects of a traditional in-person MBCR program on the gut microbiome and psychosocial symptoms. The final talk will review our burgeoning program on psychedelic-assisted therapies by describing the PAT study, which surveyed several hundred people diagnosed with cancer, healthcare providers and healthcare administrators about their knowledge, beliefs and attitudes towards psychedelic-assisted therapies.

**S4-64**

#### 2.3.1. Does Sex, Age or Ethnicity Matter? A Cross-Sectional Examination of Demographic Moderators of Mindfulness and Emotion Regulation in a Diverse Sample of People Living with Cancer

Hanna Conradi, Tina Nguyen and Linda Carlson.
University of Calgary, Calgary, Canada.

**Background/rationale or objectives/purpose**

Many cancer survivors experience chronic and distressing post-treatment psychosocial symptoms. Mindfulness-Based Interventions (MBIs) reduce psychosocial symptoms by decreasing reliance on emotion regulation strategies such as rumination and experiential avoidance (EA). However, whether demographic characteristics moderate associations between mindfulness and psychosocial symptoms through emotion regulation has yet to be explored. This study aims to (1) confirm the mediating role of rumination and EA in the relationship between mindfulness and specific psychosocial symptoms and (2) test sex and age as moderators of the mediation.

**Methodology or Methods**

This cross-sectional study assesses baseline data from 345 participants in the SEAMLESS mindfulness app trial. Patient-reported measures for trait mindfulness, rumination, experiential avoidance, psychosocial symptoms and quality of life will be collected. Structural Equation Modeling (SEM) will be employed to test whether the intervention reduces symptoms through rumination and EA and to test demographic factors such as moderators of the mediation.

**Impact on practice or results**

N = 143 participants are currently enrolled and 48.1% are female. The mean age is 61.78 (SD = 13.28). Ethnicities include Caucasian—80.5%, black—1.6%, Latin American—2.3%, South Asian—3.9%, Filipino—1.6%, Mixed or unsure—9.4%. Types of cancer diagnoses include breast—34.3%, prostate—23.8%, head and neck—4.2%, lymphoma—11.2%, gastrointestinal—13.3%, other 9.8%. Recruitment and analysis are ongoing. The results of SEM analyses will be presented.

**Discussion or Conclusions**

The results will delineate potential mediating and moderating mechanisms that may account for the beneficial effects of MBIs for survivors and inform how MBIs can be individualized to support equity, diversity and inclusion within cancer care.

**S4-94**

#### 2.3.2. Exploration of Attitudes and Beliefs toward Psychedelic-Assisted Therapies (PAT) for Cancer-Related Psychosocial Symptoms in Healthcare Providers, Patients and Policy Makers

Haley Mather, Tina Nguyen, Julie Deleemans and Linda Carlson.
University of Calgary, Calgary, Canada.

**Background/rationale or objectives/purpose**

Given growing interest in the use of psychedelics to treat psychosocial symptoms in people with cancer, this study explored attitudes toward psychedelic-assisted therapy (PAT) among healthcare providers (HCPs), policy makers/administrators and people living with cancer (PLWC) in Canada.

**Methodology or Methods**

An anonymous cross-sectional online survey utilizing a 98-item questionnaire tailored to each participant group was administered.

**Impact on practice or results**

In total, 423 Canadian stakeholders completed this survey: 78 HCPs, 333 PLWC and 12 administrators. Fifty-eight percent were women and there was wide diversity in age, ethnicity and political and religious backgrounds. Utilizing validated measures from the Attitudes Toward Psychedelics Questionnaire, 56.7% of PLWC and 81.7% of HCPs stated that they supported medical legalization of psychedelics, with administrators unanimously in favor. Additionally, 54.2% of PLWC and 77.6% of HCPs felt the use of psychedelics for cancer patients under controlled conditions was safe. Furthermore, 40.7% of PLWC and 31.7% of HCPs were concerned about motives behind psychedelic legalization, while 50% of administrators remained undecided. Regarding adverse effects, 39.2% of PLWC and 47.5% of HCPs expressed worry, with administrators split. Notably, over 50% in all groups recognized the psychedelics’ potential spiritual benefits, showed curiosity in other people’s experiences and expressed their own interest in psychedelic use.

**Discussion or Conclusions**

The survey reveals a broad yet cautious endorsement of psychedelics’ therapeutic potential among key stakeholders, signaling an evolving readiness to incorporate them into healthcare. Varied views on potential risks and benefits underscore the need for targeted education and policies that reflect these insights, ensuring the informed integration of psychedelics into healthcare based on stakeholder consensus.

**S4-102**

#### 2.3.3. The Mindful-Gut Feasibility Trial Protocol: Investigating Effects of Mindfulness-Based Cancer Recovery (MBCR) on the Gut Microbiome, Mindfulness and Gastrointestinal and Psychosocial Symptoms in People with Cancer

Jamie Petersson, Julie Deleemans, Athina Spiropoulos, Raylene Reimer and Linda Carlson.
University of Calgary, Calgary, Canada.

**Background/rationale or objectives/purpose**

Chemotherapy has enduring effects on cancer survivors’ gut microbiota, which are associated with psychosocial symptomatology. Considering the chronic gastrointestinal (GI) and psychosocial symptoms observed in cancer survivors, investigating interventions to improve gut health and psychosocial wellbeing is critical. This remains unstudied; hence, this study aims to determine if a complementary and integrative cancer care program, mindfulness-based cancer recovery (MBCR), impacts the gut microbiota and alleviates GI symptoms.

**Methodology or Methods**

This study design is a pilot proof-of-concept pre-post cohort study with a 9-week MBCR intervention. Target sample size is 15 participants. Stool samples along with GI, mindfulness and psychosocial measures will be collected pre- and post-intervention. Gut microbiota composition will be analyzed through 16S rRNA gene sequencing on stool samples. The primary outcome is feasibility of enrollment and data collection. The secondary outcomes are gut microbiota composition, mindfulness, GI and psychosocial symptoms.

**Impact on practice or results**

The recruitment and retention rates, completeness of data collection and effect sizes of the MBCR intervention on outcome measures will be assessed. We hypothesize that MBCR will result in: (1) significant alterations in the gut microbiota composition; (2) a decline in the prevalence and severity of GI and psychosocial symptoms and improved mindfulness and: (3) significant correlations between specific bacteria and GI-, mindfulness- and psychosocial-related outcomes.

**Discussion or Conclusions**

This novel study will investigate the potential impact of readily accessible, cost-effective integrative cancer care intervention on gut microbiota and GI symptoms, extending our knowledge of MBCR effects beyond measures of psychosocial health to include the gut microbiome for the first time.

### 2.4. S5 Establishing Patient-Oriented Research Priorities in Canadian Oncology Populations across the Lifespan: Illustrations of the James Lind Alliance Priority Setting Partnership

**Moderator**

Fiona Schulte.

**Summary**

There is a growing recognition of the importance of engaging patients as partners in the research process, including in the establishment of research priorities. The James Lind Alliance (JLA) Priority Setting Partnership (PSP) methodology is the gold standard for establishing patient-oriented research agendas and has been applied to dozens of health conditions worldwide. The systematic process of a JLA PSP results in a “Top 10” list of research priorities developed by patients, caregivers and clinicians that are primed for action by scientists and research funders. However, researchers report uncertainty about how to conduct rigorous, high-quality research priority setting exercises. The speakers in this symposium will describe three Canadian JLA PSPs that are at various stages and engage patients with cancer from across the lifespan. First, P. Tutelman and C. Thurston will co-present on the establishment and initial development of the Adolescent and Young Adult Cancer PSP. Next, L. Jibb will discuss the recently completed Pediatric Cancer PSP, including considerations for engaging youth in priority setting exercises. Finally, N. Nixon will describe key findings from the Metastatic Breast Cancer PSP and will outline steps to disseminate and implement the priorities. Recommendations for future PSPs in cancer care will be discussed.

**S5-65**

#### 2.4.1. Establishing a James Lind Alliance Priority Setting Partnership on Adolescent and Young Adult Cancer in Canada

Perri Tutelman ^1,2^, Chantale Thurston ^3^, Tamara Rader ^4,5^, Brianna Henry ^1^, Fiona Schulte ^1,6^, on behalf of the AYA Cancer PSP Team ^7^.

^1^  University of Calgary, Calgary, Canada.
^2^  Tom Baker Cancer Center, Calgary, Canada.
^3^  AYA CAN, Winnipeg, Canada.
^4^  James Lind Alliance, Southampton, UK.
^5^  Canadian Agency for Drugs and Technologies in Health, Ottawa, Canada.
^6^  Alberta Children’s Hospital, Calgary, Canada.

**Background/rationale or objectives/purpose**

Adolescents and young adults (AYAs) diagnosed with cancer have distinct needs and inferior outcomes compared to younger and older patients. A patient-oriented research agenda could improve care for Canadian AYAs with cancer. We report on the early development of a James Lind Alliance (JLA) Priority Setting Partnership (PSP) to establish the top 10 research priorities for AYA cancer in Canada.

**Methodology or Methods**

This project follows the JLA PSP methodology whereby patients, caregivers and clinicians come together to co-develop research priorities following a standardized, multi-step process.

**Impact on practice or results**

A call for expressions of interest to join the AYA Cancer PSP Steering Group was launched in January 2023 in both English and French. The call was open for a period of 2 weeks. A total of 78 applications were received and 16 individuals representing an equal distribution of patients/caregivers and clinicians were selected. The selection of members was based on ensuring diverse representation in terms of: geography, age, gender, race/ethnicity, cancer history and clinical role. Additionally, 33 community-based cancer organizations across the country are engaged in the PSP as project partners. The process to gather, process and verify uncertainties in the area of AYA cancer is currently underway.

**Discussion or Conclusions**

This project will result in a top 10 list of research priorities for AYA cancer in Canada identified by AYA patients, caregivers and clinicians. This patient-oriented research agenda is a crucial step towards improving outcomes and care for AYAs with cancer in Canada.

**S5-56**

#### 2.4.2. What Research Questions Matter Most to Patients? Final Results and Follow-Up of the Metastatic Breast Cancer Priority Setting Partnership

Nancy Nixon.

**Background/rationale or Objectives/purpose**

Research is fundamental to the management of cancer; however, many studies are researcher- or industry-led, with minimal input from patients and caregivers. The James Lind Alliance (JLA) is an initiative that brings patients, caregivers and clinicians together in priority setting partnerships (PSPs) to determine priorities in research. Breast cancer remains the most common cancer among women, with an estimated third of patients developing metastatic disease. With advances in treatment, women are living longer with metastatic breast cancer (MBC), in some cases many years. Here we present the results from the Canadian MBC PSP and provide an update on our current research.

**Methodology or Methods**

Following the established JLA approach, MBC patients, caregivers and health professionals were surveyed to elicit their questions regarding MBC. Research questions were generated from the survey responses and an interim prioritization survey was conducted to identify a shortlist of questions to take to a final meeting.

**Impact on practice or results**

One thousand, one-hundred and ninety-four responses were collected from 668 individuals (49% patients; 13% physicians; 9% caregivers; 4% allied health care professionals; 2% patient organization representatives; 23% other), which were refined into 62 unique questions. The interim prioritization survey was completed by 174 individuals and the top 27 questions were taken to a final meeting where MBC patients, caregivers and health professionals prioritized the questions and reached the top 10.

**Discussion or Conclusions**

The top 10 questions cover a wide range of research questions, identified by valuable stakeholders being priorities. This list has been used to inform prioritization and funding of future MBC research.

**S5-58**

#### 2.4.3. Setting a New Research Agenda in Canadian Pediatric Cancer: A James Lind Alliance Priority Setting Partnership with Patients, Survivors, Family Members and Clinicians

Lindsay Jibb
University of Toronto, Hospital for Sick Children, Toronto, Canada.

**Background/rationale or objectives/purpose**

Rigorous pediatric cancer research is needed to improve child and family outcomes during and after therapy. We aimed to conduct a Canada-wide Priority Setting Partnership with pediatric cancer patients, survivors, family members and clinicians, identifying and prioritizing research questions to direct action by funders and scientists.

**Methodology or Methods**

A steering group oversaw our James Lind Alliance approach and Canadian childhood cancer partner organizations were recruited. First, a national online survey collected research uncertainties that were collated into indicative questions. Questions were systematically checked against published evidence to identify those that were unanswered. A second survey facilitated question prioritization. A priority question shortlist was then taken to a consensus-building workshop, where the top ten priorities were decided.

**Impact on practice or results**

The respondents (n = 352) submitted over 600 potential questions in English or French. Following the removal of ‘out of scope’ questions, question collation and our evidence check, 50 questions were prioritized by 201 participants. The top 24 questions underwent final prioritization at a workshop attended by 24 survivors, family members and clinicians. The top ten priorities reflect the breadth of the pediatric cancer continuum, focusing on access to innovative therapies; physical, mental and psychosocial impacts and needed supports; preventing and treating relapsed and refractory cancers; and survivorship issues.

**Discussion or Conclusions**

Shared Canadian pediatric cancer research priorities were identified by stakeholders not typically involved in research agenda setting. The priorities suggest a call to action on minimizing barriers to accessing care, improving the capacity to implement evidence into practice and the delivery of holistic and psychosocial care.

### 2.5. S6 Engaging Stakeholders in Survivorship Research to Increase Its Reach and Impact: Lessons Learned from the Perspective of Researchers and Clinicians

**Moderator**

Sophie Lebel.

**Summary**

Collaboration among researchers, healthcare professionals, survivors and community partners is an important goal of Canada’s Strategy for Patient-Oriented Research (SPOR) to build a sustainable, accessible and equitable health care system. Such collaborations have the potential to increase the access to evidence-based programs for cancer survivors and care partners, which remains a challenge outside of major cancer centers. However, including stakeholders in research does require specific considerations.

This symposium will focus on two recent group interventions that engaged stakeholders in their adaptation, development and/or evaluation to ensure patient-centered outcomes and lessons learned from the perspectives of researchers, clinicians and patients. With the aim of increasing access to more Canadians affected by cancer, both interventions were offered via (1) community partners and (2) online. First, Jani Lamarche will report on the adaptation and pilot testing of the Fear of Recurrence Therapy to family caregivers of adult cancer survivors. Next, Nicole Rutkowski will report on a 4-week CBT and psychoeducation community-based program that addresses cancer-related fatigue. Last, Julia Parrott will report on her experience and those of other clinicians involved in the elaboration and delivery of these interventions.

The panel will discuss clinical implications and directions for engagement of stakeholders in future research.

**S6-84**

#### 2.5.1. Giving Voice to Many: The Inclusion of Stakeholders in the Adaptation and Evaluation of the Family Caregiver—Fear of Recurrence Therapy

Jani Lamarche ^1^, Rinat Nissim ^2^, Jonathan Avery ^2^, Jiahui Wong ^3^, Christine Maheu ^4^, Sylvie D. Lambert ^4^, Andrea M. Laizner ^4^, Jennifer Jones ^2^, Mary Jane Esplen ^5^ and Sophie Lebel ^1^.

^1^  University of Ottawa, Ottawa, Canada.
^2^  Princess Margaret Cancer Center, Toronto, Canada.
^3^  Cancer Chat De Souza Institute, Toronto, Canada.
^4^  McGill University, Montreal, Canada.
^5^  University of Toronto, Toronto, Canada.

**Background/rationale or objectives/purpose**

When developing interventions, including stakeholders (e.g., patients/survivors, caregivers, community/medical partners) from the onset ensures that their perspectives are thoroughly considered and integrated, ultimately enhancing the relevance, acceptability and effectiveness of interventions. In this adaptation and evaluation of the Family Caregiver—Fear Of Recurrence Therapy (FC-FORT), the scarcity of the literature concerning effective interventions addressing the fear of cancer recurrence (FCR) among family caregivers (FC), coupled with the distinct challenges faced by this group, underscores the importance of engaging stakeholders throughout the process.

**Methodology or Methods**

An advisory board [researchers (n = 10), therapists (n = 2), FC (n = 4)] was created to adapt FC-FORT. Following the adaptation, women FC (n = 28) experiencing FCR were recruited through hospitals, community partners and social media. Therapists (n = 3) with extensive experience in psychosocial oncology and offering online groups were also recruited. The advisory board met on 7 occasions to review FC-FORT’s content, manuals and recruitment strategies. Once adapted, FC completed 7 weeks of online group therapy (FC-FORT). FC and therapists completed questionnaire packages and semi-structured exit interviews to explore their experience with FC-FORT. Descriptive statistics and conventional content analyses were used.

**Impact on practice or results**

The participation rate was 76%. The satisfaction and response rates were above 80%. The stakeholder feedback highlighted overlooked FCR challenges needing to be addressed (e.g., communication), effective/ineffective aspects and feasibility issues (e.g., recruitment). The unique considerations of working with stakeholders will be discussed.

**Discussion or Conclusions**

Owing to the inclusion of stakeholder perspectives, FC-FORT’s adaptation is based on unique FC needs related to FCR. After addressing feasibility issues, FC-FORT’s efficacy is ready for testing in a randomized control trial.

**S6-108**

#### 2.5.2. Brief Intervention for Cancer-Related Fatigue: Partnering with a Patient Advisory Board and Community Partners for Sustainable Intervention Development

Nicole Rutkowski ^1^, Patricia Barrett ^2^ and Sophie Lebel ^1^.

^1^  University of Ottawa, Faculty of Social Sciences, School of Psychology, Ottawa, Canada.
^2^  Ottawa Cancer Foundation, Ottawa, Canada.

**Background/rationale or objectives/purpose**

Approximately a third of cancer survivors will continue to experience moderate-to-severe fatigue for years post-treatment. Despite evidence-based interventions and guidelines available for cancer-related fatigue (CRF), many interventions are not sustained after research completion in community or hospital settings. Several barriers to the sustainability of interventions exist; however, partnership with community organizations can result in more tailored programming that meets the needs of an organization and increases the likelihood of long-term implementation.

**Methodology or Methods**

An evidence-based intervention for cancer-related fatigue was adapted and updated to the local community context in Ottawa, Canada, using the Knowledge-to-Action framework and in collaboration with a community partner and patient advisory board. A hybrid effectiveness implementation Type II was used to simultaneously evaluate whether the intervention remained effective while evaluating implementation efforts. Implementation outcomes will be presented such as patient satisfaction, retention and qualitative findings from focus groups. Long-term maintenance and continued work with community partners will be discussed.

**Impact on practice or results**

This intervention aims to bridge the gap between evidence-based recommendations and community implementation. The importance of involving patient and community partners will be discussed in relation to developing a patient-centered intervention that is tailored to the needs of a community organization. Lessons learnt and future directions will be presented.

**Discussion or Conclusions**

CRF has been reported as one of the most significant and debilitating post-treatment symptoms. The translation of knowledge to action through more widely available, sustainable and affordable programs is paramount to meeting the needs of an increasing cancer survivorship population.

**S6-61**

#### 2.5.3. Clinical Perspectives: Reflections on Facilitator Inclusion in the Adaptation and Evaluation of the Family Caregiver—Fear of Recurrence Therapy and Cancer-Related Fatigue Interventions

Julia Parrott ^1^, Melanie Baruch ^2^ and Patricia Barrett

^1^  Yarrow Psychotherapy, Ottawa, Canada.
^2^  Cancer Care Manitoba, Winnipeg, Canada.

**Background/rationale or objectives/purpose**

To improve the relevance, acceptability and sustainability of the Family Caregiver—Fear Of Recurrence Therapy and cancer-related fatigue interventions, facilitators (n = 3) with extensive experience in psychosocial oncology and online group therapy were recruited to provide clinical expertise and facilitate groups. Including therapists in the development of a program can make a difference in a program being successfully implemented by other clinicians.

**Methodology or Methods**

Facilitators were consulted using advisory boards, semi-structured interviews, weekly supervision and questionnaire packages throughout the development, evaluation and refinement of both interventions. In addition, the research team evaluated the fidelity of intervention administration weekly and feedback was provided regularly.

**Impact on practice or results**

In this presentation, facilitators will reflect on their inclusion in the research process and how it impacted the delivery of interventions, the participant’s experience of the interventions and the facilitators’ experience running an online group within a research study. Examples of the facilitator’s recommendations include adjusting the length of a session so there would be more time for connection and discussion and adaptations that aim to increase the accessibility of the intervention manuals.

**Discussion or Conclusions**

This study has shown that the facilitator’s inclusion in developing clinical interventions is feasible and meaningful. In the discussion, presenters will reflect on their experiences throughout the study.

## 3. Final Category: B. Cancer Treatment-Related Symptom and Toxicity Management

**4**

### 3.1. Economic Burden and Impact of Cancer Treatment and Post-Treatment for Adults Living in Rural Settings in Canada

Melba D’Souza ^1^, Hannah Nette ^2^, Subrahmanya Karkada ^1^ and Ashwin Nairy ^3^.

^1^  Thompson Rivers University, Kamloops, Canada.
^2^  InspireHealth Supportive Cancer Care, Vancouver, Canada.
^3^  University of British Columbia, Vancouver, Canada.

**Background/rationale or objectives/purpose**

Supporting cancer survivors in cancer care is essential for enabling individuals to manage the physical and psychosocial consequences of cancer treatment actively. Engaging newly diagnosed patients is crucial for cancer-supportive care delivery through healthcare and community services. Financial toxicity contributes to psychosocial distress among cancer patients and survivors and contextual factors distinctive to rural settings further impact patient experiences.

This research aims to assess the economic burden and impact of cancer on individuals coping with the consequences of cancer treatment in British Columbia. This study investigates associations between financial toxicity and psychosocial outcomes among a sample of rural cancer patients.

**Methodology or Methods**

A mixed methods approach was employed, enrolling cancer survivors who willingly participated in the research. Participants provided insights into facilitators and barriers faced during cancer treatment. Data from 151 participants were utilized to estimate multivariable regression models predicting the impact of cancer based on financial toxicity and its interplay with psychosocial outcomes.

**Impact on practice or results**

Significant associations were observed between financial toxicity and psychosocial outcomes among the rural participants. Economic challenges were associated with higher age groups, gender disparities, living with caregivers and treatment barriers among diverse sub-populations in post-treatment, recovery and coping.

**Discussion or Conclusions**

This study reveals essential components for integrating cancer supportive care into cancer care, highlighting the necessity for adaptable approaches tailored to the rural context. The confirmed link between financial toxicity and psychosocial well-being in rural settings highlights the importance of providing healthcare providers with the tools and resources to communicate with patients about financial distress.

**26**

### 3.2. Systemic Treatment for Brain Metastatic Triple-Negative Breast Cancer: A Systematic Review and Meta-Analysis

Linh Vu, Gordon Daly, Oluwapelumi Ogunlowo and Gavin Dowling.
Royal College of Surgeons in Ireland, Dublin, Ireland.

**Background/rationale or objectives/purpose**

Patients with advanced triple-negative breast cancer (TNBC) develop brain metastases (BM) in nearly 50% of cases. The management of this patient population is varied, involving a combination of local therapy and/or systemic treatment. Drug development for brain metastatic TNBC (TNBCBM) remains primitive, with few having reached clinical trials and even fewer currently approved. This systematic review summarizes and evaluates approved and emerging systemic therapies for patients with TNBCBM.

**Methodology or Methods**

Systematic search was conducted using databases PubMed, Clarivate Analytics/Web of Science, Embase.com and the Wiley/Cochrane Library. Eligible articles included clinical trials and prospective and retrospective studies reporting on median progression-free survival (mPFS), median overall survival (mOS) and/or objective response rate (ORR) of different systemic therapies for TNBCBM.

**Impact on practice or results**

Thirteen studies fulfilled the inclusion criteria, reporting on chemotherapy, immunotherapy, antibody-drug conjugates and tyrosine kinase inhibitors. Lu et al., assessing Bevacizumab administration prior to Etoposide and Cisplatin (BEEP regimen), reported the highest CNS-ORR of 100%. Chang et al.’s retrospective analysis of patients treated with a range of BBB-crossing and non-crossing chemotherapeutic agents yielded the highest mPFS of 32.8 months, while Du et al. demonstrated the highest mOS of 23.9 months for patients treated with immune-checkpoint inhibitors.

**Discussion or Conclusions**

The comprehensive systematic review showed that when used in combination with local treatment modalities, systemic therapies provided a marked benefit in controlling extra-cranial disease and preventing additional seeding to the brain. However, the small number of studies and heterogeneity of data emphasize the urgent need for further inclusions in clinical drug trials of this subgroup of patients.

**34**

### 3.3. Demonstrating the Utility of Patient-Reported Outcome-Derived Symptom Complexity in Predicting Overall Survival among Patients with Cancer in Alberta, Canada

Linda Watson, Claire Link, Siwei Qi, Se’era Anstruther, Andrea DeIure and Lisa Barbera.
Alberta Health Services, Calgary, Canada.

**Background/rationale or objectives/purpose**

Patient-Reported Outcome measures like the revised Edmonton Symptom Assessment System (ESAS-r) help patients accurately report their symptom burden. However, the magnitude of information gathered can be difficult to utilize in practice. This retrospective study aimed to demonstrate the utility of a symptom summary score, collected at a baseline timepoint, in predicting overall survival among patients with cancer.

**Methodology or Methods**

Patients aged 18 and older who had their first consult with Cancer Care Alberta (CCA) between 1 October 2019 and 1 April 2020, and completed the ESAS-r within 30 days following this consult were eligible for inclusion. A symptom complexity score (low, moderate and high) was generated from each completed ESAS-r, using a unique algorithm developed in CCA. Cox proportional hazards models were constructed to determine if symptom complexity and other demographic and clinical factors were associated with overall survival. The observation period for assessing survival was 1 October 2019–30 April 2023.

**Impact on practice or results**

In total, 5841 patients were included. At the end of the observation period, 68.6% of patients were alive. The Cox model revealed that, compared to patients with low symptom complexity, moderate-complexity patients had a 69% higher risk of death (HR = 1.69, 95% CI: 1.50–1.90, *p* < 0.001) and high-complexity patients had a 142% higher risk of death (HR = 2.42, 95% CI: 2.15–2.72, *p* < 0.001).

**Discussion or Conclusions**

The symptom complexity algorithm developed in CCA was shown to effectively predict the patients’ overall survival. Other cancer care programs which routinely utilize ESAS-r may want to consider utilizing a similar summary score to improve the interpretability of the information collected.

**80**

### 3.4. Assessing the Social Adjustment Difficulties in Survivors of Pediatric Acute Lymphoblastic Leukemia Treated without Cranial Radiation Therapy

Heide Adham, Kaelyn McDonald-Wirasinghe, Jenny Duong, Caitlin Forbes, Mehak Stokoe and Fiona Schulte.

**Background/rationale or objectives/purpose**

Early treatment for acute lymphoblastic leukemia (ALL) used cranial radiation therapy (CRT) to prevent leukemic cells from entering the central nervous system (CNS). Current treatment protocols have shifted away from CRT to treat ALL due to CRT’s link to neurocognitive deficits. With a growing population of ALL survivors, more research is needed to evaluate the long-term effects of modern treatments. The goal of this study was to characterize the social adjustment difficulties experienced by survivors of pediatric ALL who have not undergone CRT.

**Methodology or Methods**

We assessed survivors aged 8–17 at least two years after concluding treatment. Participants were >2 years off treatment or >5 years from diagnosis with no neurodevelopmental disorders or cognitive impairment. Survivors of pediatric ALL (n = 25) and non-CNS solid tumors (n = 18) were recruited from the long-term survival clinic at the Alberta Children’s Hospital. Healthy controls (n = 33) were included from a previous study.

Social adjustment was assessed using the Adaptive Behaviors Assessment System Third Edition Social Adaptive Domain. Group comparisons were made using Analysis of Variance tests. Hierarchical regression analyses were conducted to explore possible predictors of social adjustment.

**Impact on practice or results**

Survivors of ALL and solid tumors showed significantly worse cognitive–executive functioning than healthy controls. No significant differences in social adjustment or cognitive–executive functioning were observed between survivors of ALL and solid tumors.

**Discussion or Conclusions**

These findings indicate that survivors of pediatric ALL and non-CNS solid tumors experience poorer social adjustment. Identifying children who are at risk of developing social adjustment difficulties offers the best chance to promote healthy social development.

## 4. Final Category: C. Cancer Care across the Life Span (Children, Adolescents and Young Adults, Adults and Older Adults)

**35**

### 4.1. Coming Together through Feeling Apart: Exploring the Feasibility of a Novel Group Intervention for People in Their 30s and 40s Experiencing Different Types and Stages of Cancer

Dana Male ^1,2^, Sara Beattie ^1,2^, Perri Tutelman ^1,2^, Fiona Schulte ^2,3^, April Boychuk ^1^, Jennifer Pink ^4^ and Jessie Moorman ^5^.

^1^  Tom Baker Cancer Center, Calgary, Canada.
^2^  University of Calgary, Calgary, Canada.
^3^  Alberta Children’s Hospital, Calgary, Canada.
^4^  University of Waterloo, Waterloo, Canada.
^5^  Calgary Epilepsy Program, Calgary, Canada.

**Background/rationale or objectives/purpose**

People in their 30s and 40s affected by cancer experience a significant disruption of life plans. They also face isolation and challenges accessing well-matched services tailored to their life stage. To address the unique unmet psychosocial needs of this population, a six-week online psychotherapy group (‘PIVOT’) was developed and piloted.

**Methodology or Methods**

Recruitment included people in their 30s/40s who received treatment at the Tom Baker Cancer Center within the past five years, inclusive of any gender, cancer type or illness stage. This approach represented a departure from our center’s tumor-specific service model and commonplace practice of distinguishing between services for those with early-stage or advanced cancers. This study aimed to evaluate the feasibility of a six-week virtual psychotherapy group for people of diverse genders, types of cancer and disease stages.

**Impact on practice or results**

The sample included two cohorts (n = 8; n = 10) of participants with the following cancers: five breast, three lymphoma, two cervical, two leukemia, one gastrointestinal, one colorectal, one brain, one ovarian, one testicular and one melanoma. Twelve participants identified as female and six as male. The findings suggest the feasibility of the recruitment and implementation of PIVOT. Demographic/oncological diversity did not appear to represent a barrier to participation.

**Discussion or Conclusions**

This study challenges the practice of stratifying services based on common gender, cancer diagnosis or stage, suggesting that when patients in their 30s/40s are afforded opportunities to connect with age-matched peers, other factors that may be more important to match with other populations may be less relevant. Future directions include a qualitative data collection of participants’ disclosures of group cohesion.

**48**

### 4.2. Understanding the Individual Challenges and Experiences of Young Adults with High-Grade Glioma

Kaviya Devaraja ^1,2^, Maureen Daniels ^3,4^, Derek S. Tsang ^5^, Kim Edelstein ^4,6^, Julie Bennett ^7,4^, Cheryl Kanter ^3,4^, Warren Mason ^3^, Abha A. Gupta ^2,8^ and Jonathan Avery ^2,9^.

^1^  Institute of Medical Science, University of Toronto, Toronto, Canada.
^2^  Adolescent and Young Adult Program, Department of Supportive Care, Princess Margaret Cancer Center, Toronto, Canada.
^3^  Pencer Brain Tumor Center, Princess Margaret Cancer Center, Toronto, Canada.
^4^  Department of Supportive Care, Princess Margaret Cancer Center, University Health Network, Toronto, Canada.
^5^  Radiation Medicine Program, Princess Margaret Cancer Center, University Health Network, Toronto, Canada.
^6^  Department of Psychiatry, University of Toronto, Toronto, Canada.
^7^  Adolescent and Young Adult Program, Department of Supportive Care, Princess Margaret Cancer Center, Toronto, Canada.
^8^  Division of Medical Oncology and Hematology, Princess Margaret Cancer Center, Toronto, Canada.
^9^  School of Nursing, University of British Columbia, Vancouver, Canada.

**Background/rationale or objectives/purpose**

Young adults (YA) (aged 18–39) with high-grade glioma (HGG) experience headaches, seizures, cognitive changes, fatigue and nausea similar to older adults with the same diagnosis, but the impact may differ. YAs are at a life stage characterized by seeking autonomy, identity development, relationship building, embarking on a career and family planning. This study explores the experiences and challenges faced by YAs diagnosed with HGG to inform life-stage-appropriate support and resources.

**Methodology or Methods**

In this simultaneous mixed methods (Quantitative–Qualitative) study, we surveyed YA HGG patients at the Princess Margaret Cancer Center, exploring demographics, symptom experience and level of satisfaction with current care. We also interviewed participants, asking questions about their illness experience and needs. Survey responses were summarized using descriptive statistics. Thematic analysis of the interviews was conducted by creating a coding framework to determine emerging themes using NVivo 10.

**Impact on practice or results**

To date, 7/15 participants completed both surveys and interviews (three men; four women; mean age 30.57 (SD = 6.25), range 19–37). The results highlight the challenges of living with HGG because of (1) a disruption in completing education and achieving other life-stage-specific goals due to cognitive changes and seizures; (2) a lack of YA-specific education, advocacy, hope dialogue and support; and (3) limited opportunities for community building and connecting.

**Discussion or Conclusions**

These results highlight the importance of developing life-stage-specific resources, supports and community to help YAs achieve life-stage-specific goals. The next steps include completing recruitment and analysis and creating educational tools to better inform and support these YAs.

**50**

### 4.3. Teen Cancer Connection: Needs Assessment to Inform Development of an Adolescent and Young Adult Oncology Program at a Large Canadian Pediatric Center

Rebecca Cote ^1^, Chana Korenblum ^1,2^, David Brownstone ^1^ and Sarah Alexander ^1^.

^1^  The Hospital for Sick Children, Toronto, Canada.
^2^  Princess Margaret Cancer Center, Toronto, Canada.

**Background/rationale or objectives/purpose**

Adolescents and young adults with cancer (AYA) have unique biopsychosocial needs, often inadequately addressed by healthcare systems designed for younger or older patients. In response, developmentally tailored programs have been launched around the world, primarily in adult hospitals. To inform the creation of the first known AYA program in a Canadian pediatric center, a needs assessment was conducted.

**Methodology or Methods**

Surveys were administered to AYAs (age 12+) and oncology healthcare professionals at a large pediatric hospital. Fifty-one AYAs diagnosed with leukemia/lymphoma, brain tumors or solid tumors participated, 92.2% within 5 years of diagnosis. One hundred present of AYAs identified that they have unique psychosocial needs differing from younger children, with 70% reporting needs not adequately addressed by their healthcare team. Additionally, 11.8% often or always spoke with their team alone and infrequently discussed topics were related to sexual health, transition to adult care and fertility preservation. Program elements requested by AYAs were social engagement and mental health support.

Sixty-one psychosocial, nursing and medical professionals participated, identifying mental health, body image and impact on school/work as important AYA psychosocial topics. Psychosocial topics were discussed “often” by 25% and “sometimes” by 51%, with top barriers being lack of time and training and parental presence. Only 13% felt adequately prepared for these conversations.

**Impact on practice or results**

The results of this needs assessment will inform the development and evaluation of the new program, Teen Cancer Connection.

**Discussion or Conclusions**

The first in Canada, this program will fill an important clinical care gap and has the potential to expand in the future to support the development of similar programs across the country.

**52**

### 4.4. Fear of Cancer Recurrence Experienced by Pediatric Survivors of Childhood Cancer: A Scoping Review

K Brooke Russell ^1^, Araby Roberts ^1^, Holly Wright ^1^, Brianna Henry ^1^, Oserekpamen Favour Omobhude ^2^, Pauline Holmer ^3^, Rachelle Drummond ^1^, Tessa Verhesen ^4^, Caitlin Forbes ^1^, Mehak Stokoe ^1^, Lianne Tomfohr-Madsen ^5^ and Fiona Schulte ^1^.

^1^  University of Calgary, Calgary, Canada.
^2^  New York Medical College, Valhalla, USA.
^3^  University of Lucerne, Lucerne, Switzerland.
^4^  University of Alberta, Edmonton, Canada.
^5^  University of British Columbia, Vancouver, Canada.

**Background/rationale or objectives/purpose**

In contrast to the extensive literature on the fear of cancer recurrence (FCR) experienced by adults, the literature evaluating pediatric FCR has just begun to emerge. Given the rapidly expanding body of work assessing FCR in childhood and adolescence, a scoping review was conducted to synthesize the existing findings. We aimed to assess (1) the characteristics and methods of this literature, (2) how pediatric FCR has been measured and (3) the extant knowledge of FCR experienced by pediatric survivors of cancer.

**Methodology or Methods**

Inclusion criteria were (1) original reports, (2) participants diagnosed with cancer before age 18, (3) current mean age under 18, (4) FCR was explicitly measured (quantitatively) or captured (qualitatively), (5) FCR was from child perspective and (6) in English. Exclusion criteria were (1) case studies and (2) grey literature. Three databases were searched (Embase, MEDLINE and PsycINFO), as well as reference lists from included studies. All data were extracted in Covidence by a single author.

**Impact on practice or results**

Of the 3902 studies identified, 19 were included. The studies (published 1991–2023) encompassed diverse geological locations, study designs and measurement methods. Few assessed FCR as a primary aim (n = 6, 32%). Pediatric FCR was experienced by 43–90% of young survivors. FCR was often positively associated with age and somatic symptoms and negatively associated with quality of life and emotional functioning.

**Discussion or Conclusions**

FCR is a prevalent concern for children and adolescents. Additional evidence is needed to explore and confirm the current findings. Future pediatric FCR studies should aim to align with published priority research areas.

**75**

### 4.5. Introducing the FORT-Parent Study: An Adaptation of the Fear of Recurrence Therapy to Parents of Pediatric Cancer Survivors

Sophie Lebel ^1^, Celeste Holy ^1^, Janelle Gibson-Haerle ^2^, Jeff Brisson, Lauriane Giguère ^1^, Kimberly McMillan ^1^, Perri Tutelman ^2^, Heloise Sirois-Leclerc ^3^, Wendy Pelletier ^2^ and Fiona Schulte ^4^.

^1^  University of Ottawa, Ottawa, Canada.
^2^  University of Calgary, Calgary, Canada.
^3^  Children Hospital of Eastern Ontario, Ottawa, Canada.
^4^  University of Calgary, Calgary, Canada.

**Background/rationale or objectives/purpose**

Fear of cancer recurrence FCR can be addressed in cancer survivors with brief interventions, such as the FORT group intervention. However, none of these interventions have been tested with parents of childhood cancer survivors. The goals of the present study are (1) to work with an advisory board composed of researchers, clinicians and parents to iteratively adapt the six-week FORT intervention for parents (FORT-Parent) and (2) test the acceptability, feasibility and effect size of FORT-Parent in a multimethods randomized pilot study.

**Methodology or Methods**

Inclusion criteria: (1) parent (or primary caregiver) of a survivor of childhood cancer, (2) presenting with clinical levels of FCR and (3) access to a computer with an internet connection. Participants will be randomized to the intervention or to a wait-list control group, in which case they will be offered the intervention after the study. Methods: parents will be recruited directly via clinicians, databases of survivors of childhood cancer and community support partners. Feasibility criteria: (1) ability to recruit and randomize 36 parents in 12 months, (2) ability to deliver FORT-Parent to 29 parents in 15 months, (3) 80% of participants completing 5 out of 6 sessions, (4) complete measures for at least 90% of participants, (5) ability to deliver FORT-Parent as intended and (6) satisfactory ratings > 80%.

**Impact on practice or results**

The adaptation of the FORT intervention to parents is underway.

**Discussion or Conclusions**

Our proposed research has direct implications for clinical services’ development to improve the quality of life of parents of childhood cancer survivors.

**82**

### 4.6. The Gendered Nature of Body Image and Social Adjustment in Survivors of Pediatric Acute Lymphoblastic Leukemia and Solid Tumors

Jenny Duong ^1^, Sara Cho ^1^, Mehak Stokoe ^2^, Kaelyn McDonald-Wirasinghe ^3^, Caitlin Forbes ^1^, Brianna Henry ^1^, Victoria Forster ^4^, Janelle Gibson-Haerle ^1,5^, Michael Taccone ^6,7,8^ and Fiona Schulte ^1,9^.

^1^  Department of Oncology, Division of Psychosocial Oncology, Cumming School of Medicine, University of Calgary, Calgary, Canada.
^2^  Werklund School of Education, University of Calgary, Calgary, Canada.
^3^  Department of Neuroscience, University of Calgary, Calgary, Canada.
^4^  Women’s College Hospital, Toronto, Canada.
^5^  Ottawa Hospital Research Institute, University of Ottawa, Ottawa, Canada.
^6^  Department of Surgery, Division of Neurosurgery, University of Ottawa, Ottawa, Canada.
^7^  Childhood Cancer Survivor Canada, Toronto, Canada.
^8^  Faculty of Health Sciences, Ontario Tech University, Oshawa, Canada.
^9^  Hematology, Oncology, and Transplant Program, Alberta Children’s Hospital, Calgary, Canada.

**Background/rationale or objectives/purpose**

Over 80% of youths with cancer survive in Canada, but many survivors face social adjustment difficulties. Some evidence shows that gender and body image relate to social adjustment. Considering the socialization of gender norms during the pediatric development of self-concept, the current study aimed to examine correlations between gender typicality and body image and their relation to social adjustment in pediatric survivors.

**Methodology or Methods**

Survivors (*n* = 35, *M*_age_ = 13.22, 42.9% girls and 57.1% boys) and their parents were recruited from the local survivorship clinic in Calgary, Canada. Eligible participants were aged 8–17 years, diagnosed with ALL or a solid tumor, were ≥24 months off-treatment, English-speaking and had no cancer relapse or significant cognitive impairment. Survivors completed measures on their gender (Gender Typicality Scale), body image (Piers-Harris Self-Concept Scale 3—Physical Appearance and Attributes domain) and social adjustment (Children’s Loneliness and Social Dissatisfaction Scale, with higher scores indicating lower loneliness). Parent-proxy reports of social adjustment were also completed (BASC-3 Social Withdrawal subscale). Analyses included Pearson’s correlations and multiple regressions.

**Impact on practice or results**

Gender typicality and body image were marginally correlated (*r* = 0.299, *p* = 0.091). Greater gender typicality and a positive body image predicted lower self-reported loneliness (*β* = 0.309 and 0.366, *p* = 0.009). These variables were not significantly related to parent-proxy social adjustment.

**Discussion or Conclusions**

As pediatric survivors of cancer navigate gendered norms in their development and potential changes in body image, early psychosocial screenings and interventions are recommended to promote positive social adjustment in this population. Considerations for the psychosocial nature of gender may also benefit clinical practice.

**86**

### 4.7. A Study in Progress: Exploring Pediatric Oncology Healthcare Providers’ Barriers and Facilitators for Physical Activity Referral

Hannah Cripps, ^1^, Emma McLaughlin, ^1^, Laura St. John, ^1^, Fiona Schulte, ^2^, Amanda Wurz, ^1,3^ and S. Nicole Culos-Reed, ^1,4,5^

^1^  Faculty of Kinesiology, The University of Calgary, Calgary, Canada.
^2^  Department of Oncology, Division of Psychosocial Oncology, Cumming School of Medicine, University of Calgary, Calgary, Canada.
^3^  School of Kinesiology, University of the Fraser Valley, Chilliwack, Canada.
^4^  Department of Oncology, Cumming School of Medicine, University of Calgary, Calgary, Canada.
^5^  Department of Psychosocial Resources, Tom Baker Cancer Center, Calgary, Canada.

**Background/rationale or objectives/purpose**

While physical activity (PA) is considered safe and beneficial for children and adolescents diagnosed with cancer, most are not physically active enough to experience the potential benefits. Healthcare providers (HCP), often seen as trusted sources of health information, can effectively increase patients’ PA levels through referral to PA opportunities. The ongoing Implementation of Physical Activity for Children and adolescents on Treatment (IMPACT) trial provides an opportunity to assess why HCPs refer, do not refer and their barriers and facilitators to referring patients to PA opportunities in general.

**Methodology or Methods**

We will recruit up to 10 HCPs from the pediatric oncology teams at Stollery and Alberta Children’s Hospitals. Potential participants will be recruited via email and in-person recruitment strategies. Semi-structured interviews informed by the capability, opportunity and motivation behavior (COM-B) model will explore barriers and facilitators that pediatric oncology HCPs face when referring to PA.

**Impact on practice or results**

Demographic characteristics such as age, profession and years of experience will be used to describe the participants. Interviews will be analyzed using descriptive content analysis providing insight into HCP capabilities, opportunities and motivations to provide PA referral to IMPACT and other PA resources (e.g., educational materials, exercise programs).

**Discussion or Conclusions**

Identifying barriers and facilitators that pediatric oncology HCPs face when referring to PA will help inform efforts to increase referrals to the IMPACT trial specifically and PA opportunities generally. Increased referrals may lead to more children and adolescents with cancer being physically active, helping to manage the side-effects of treatment and enhancing quality of life.

**90**

### 4.8. Understanding the Experiences of Physical Activity, Body Image and Quality of Life in Young Adult Males Living with and beyond Cancer

Tana Dhruva, Jenna Sim, Chad W. Wagoner, Sarah J. Kenny, David M. Langelier and S. Nicole Culos-Reed.
University of Calgary, Calgary, Canada.

**Background/rationale or objectives/purpose**

For young adults (YA) living with and beyond cancer, diagnosis and cancer treatment can result in physical changes negatively impacting body image (BI) and quality of life (QOL). Physical activity (PA) is an evidence-based tool that may positively impact both BI and QOL. However, most research to date has focused on YA females. To address this gap, we explored the experiences of PA, BI and QOL in YA males living with and beyond cancer.

**Methodology or Methods**

A mixed-methods study was conducted with YA males (20–39 years old) diagnosed with cancer. Participants were recruited through pre-existing studies, support organizations and social media. Self-reported questionnaires assessed PA (Godin Leisure-Time Exercise Questionnaire), BI (Body Image Scale) and QOL (Functional Assessment of Cancer Therapy). Descriptive statistics summarized sample characteristics and questionnaire data. Semi-structured interviews analyzed using interpretive descriptions facilitated a deeper understanding of PA, BI and QOL.

**Impact on practice or results**

Participants (*n* = *9*; *median age 38*) reported participating in 180 min of activity/week and reported a leisure score index of 19 (range 15–90), which is considered moderately active. Participants demonstrated low BI distress (scores ranged from 11–17; median = 14) and above-average QOL well-being (scores ranged from 62–79; median = 68). Themes from seven interviews were (1) a loss of identity due to cancer, (2) building autonomy and identity using PA and (3) perceiving one’s body through the eyes of others.

**Discussion or Conclusions**

PA in YA males may be an important way to rebuild identity and BI following cancer. Enhancing YA male PA participation may afford potential BI and QOL benefits.

**114**

### 4.9. Traumatic Stress in Family Caregivers of Pediatric and Adult Patients with Acute Leukemia: Preliminary Mixed-Methods Results from a Prospective Observational Longitudinal Study

Stephanie Nanos ^1,2,3^, Lindsay Jibb ^1,3^, Carmine Malfitano ^2,1,3^, Sarah Alexander ^1,3^ and Gary Rodin ^2,3^.

^1^  The Hospital for Sick Children, Toronto.
^2^  The Princess Margaret Cancer Center, Toronto.
^3^  The University of Toronto, Toronto.

**Background/rationale or objectives/purpose**

Acute leukemia (AL) is a life-threatening blood cancer with acute onset, which requires intensive treatment and is associated with severe physical/psychological symptoms, which can be highly traumatic for family caregivers (FCs). Consequently, traumatic stress (TS) may be common in this population and increase the risk of poor health outcomes. However, systematic assessments and approaches for its prevention and treatment are lacking. We aim to determine the prevalence, severity, longitudinal course and predictors of TS in FCs of pediatric and adult patients with AL in the first year after a new diagnosis and to understand their lived experience and supportive care needs.

**Methodology or Methods**

We aim to recruit 223 FCs from two metropolitan cancer care sites within 3 months of admission. Quantitative data, including assessments of TS and related outcomes, are collected from self-report questionnaires on enrolment and 1, 3, 6, 9 and 12 months after admission and will be analyzed using descriptive/machine learning approaches. A subset of FCs (n = 50) will participate in semi-structured qualitative interviews at 3, 6 and 12 months which will be collected/analyzed using grounded theory.

**Impact on practice or results**

To date, 94 FCs (i.e., 76 parents, 15 partners and 3 adult children) have been recruited. In total, 76.6% report clinically significant TS at any one timepoint, which persists overtime such that 88.9% continue to report TS at ≥1 subsequent timepoints. Preliminary interview findings show that TS negatively impacts FC self-care.

**Discussion or Conclusions**

TS is prevalent in the FCs of patients with AL across the life course. Early intervention for its prevention and alleviation is needed to improve family-centered care quality.

**133**

### 4.10. A Longitudinal Investigation of Quality of Life in Children Attending Children’s Cancer Camp

Intisar El Houjairy ^1^, Caitlin Forbes ^2^ and Fiona Schulte ^2^.

^1^  Faculty of Science, University of Calgary, Calgary, Canada.
^2^  Department of Oncology, Cumming School of Medicine, University of Calgary, Calgary, Canada.

**Background/rationale or objectives/purpose**

Children impacted by cancer, through their own diagnosis or that of a family member, can experience psychosocial challenges such as loneliness. Children’s cancer camps have been recognized for fostering meaningful relationships and contributing to participants’ overall well-being. This study explores the impact of Kids Cancer Care Camps on the general quality of life (QOL) in children affected by cancer.

**Methodology or Methods**

Nineteen children (mean age = 11.4 years, SD = 2.0) attending a cancer camp, as well as their parents, were recruited to complete questionnaires at three time points: before camp, immediately post-return and three months afterwards. QOL was assessed through the Pediatric Quality of Life Inventory (PedsQL) 4.0 Generic Core self-report and parent-proxy questionnaire. Differences in QOL were evaluated using a paired-sample *t*-test.

**Impact on practice or results**

Parent-reported QOL was higher post-return (M = 86.0, SD = 16.21) compared to pre-camp (M = 76.1, SD = 20.43); *t*(18) = −2.73, *p* = 0.014. However, no difference was seen between pre-camp (M = 75.8, SD = 19.81) and three months later (M = 77.2, SD = 22.47). Self-reported scores did not reflect any changes in QOL over time.

**Discussion or Conclusions**

Overall, parents and children reported good QOL. The parent-reported improvement in QOL, post-camp, was not reflected in the children’s self-reports. This disparity in perception may stem from challenges in children’s self-evaluation or optimistic parental expectations and warrants further investigation. Our study was limited by a small sample size; however, it appears that cancer camps may support QOL. Future work should be carried out to better understand the psychosocial impact of attending a cancer camp.

**144**

### 4.11. Empowering Pediatric Sibling Hematopoietic Stem Cell Donor Voices through Digital Storytelling

Andrea Winther Klippenstein ^1,2^ and Christina West ^1,3^.

^1^  University of Manitoba, Winnipeg, Canada.
^2^  Red River College Polytech, Winnipeg, Canada.
^3^  University of Victoria, Victoria, Canada.

**Background/rationale or objectives/purpose**

This participatory, rights-based study sought to empower pediatric sibling donors in hematopoietic stem cell transplantation to share their donation stories through digital storytelling (DS).

**Methodology or Methods**

Six siblings between the ages of 12 and 21 donated stem cells to their brother or sister while they were under 18 years old. Research partners attended three meetings at the University of Manitoba through Zoom or in person.

Research partners expressed their donation stories using DS and participated in the thematic analysis of three digital stories. The primary researcher (AWK) and a secondary researcher (CW) took field notes to document the research experience. Two discussions were held with research partners on using DS and knowledge translation and AWK recorded, transcribed and analyzed these discussions using interpretive descriptions.

**Impact on practice or results**

The digital stories revealed six themes about the pediatric sibling stem cell donor experience: after-procedure thoughts, sibling support, hope, family experience, connections and pessimism and acceptance. AWK identified four themes in the field notes and research partner discussion transcripts related to the participatory, arts-based research process: community and connections between donors, the use of digital storytelling as a new research method, the therapeutic benefits of creating digital stories and the importance of privacy within the sibling donor experience.

**Discussion or Conclusions**

This study aimed to highlight the critical need for a greater understanding of the pediatric sibling stem cell donation experience and explore the potential use of DS to integrate pediatric sibling donor voices in future practices. The aim is to promote research with children by involving them in participatory approaches.

**147**

### 4.12. Advancing Mental Health Support for Early-Age-Onset Colorectal Cancer Patients and Caregivers

Zahra Askari ^1^, Chelsea Butler ^1^ and Patil Mksyartinian ^2^.

^1^  Colorectal Cancer Canada, Montreal, Canada.
^2^  Colorectal Cancer Canada, Montreal-Toronto, Canada.

**Background/rationale or objectives/purpose**

This study examines the crucial gaps that Canadian patients and caregivers with early-age-onset colorectal cancer (EAO CRC) encounter in obtaining mental health support. Treatment procedures were disseminated after a careful analysis of data from Colorectal Cancer Canada’s Never Too Young survey, which involved 108 EAO CRC patients and 20 caregivers and ran from 12 December 2022 to 1 May 2023.

**Methodology or Methods**

The psychological component revealed striking data: 90.1% of them withdrew from social interactions and 36.0% had ongoing emotional tiredness. Remarkably, 93.6% struggled with the widespread fear of cancer returning, which affected 65.5% of their everyday activities. Caregivers were equally stressed: 87.5% of them showed concern about the mental health of their loved ones and 31.3% reported continuous emotional exhaustion.

**Impact on practice or results**

Significant gaps in care are highlighted by this study, including awareness limitations, long-running diagnosis processes and severe mental health challenges. Recommendations include re-evaluating the CRC screening age for better early detection, increasing support for depression, anxiety and fear of reoccurrence and integrating mental health support into EAO CRC patient treatment.

**Discussion or Conclusions**

In conclusion, this study provides a thorough understanding of the difficulties experienced by EAO CRC patients and caregivers, in addition to highlighting the importance of advocating for a patient-centric strategy. Closing the knowledge gaps, improving communication and providing strong mental health support can act as a safety net, reducing the financial, emotional and physical costs associated with the EAO CRC process. We envision leveraging our findings to offer a roadmap for improving the quality of care for this vulnerable population.

## 5. Final Category: D. Community-Based and Volunteer Cancer Care Services

**40**

### 5.1. Reflections on Wellspring Alberta Members’ Experience about Community Psychosocial Support

H. M. Ashraf Ali ^1^, Stephanie Durante ^2^ and Josh Worley ^2^.

^1^  Wellspring Alberta, Edmonton, Canada.
^2^  Wellspring Alberta, Calgary, Canada.

**Background/rationale or objectives/purpose**

The central objectives of this study were to conduct impact evaluations of programs and supportive services offered by Wellspring Alberta for cancer patients, survivors, caregivers and those bereaved. In addition, other objectives were to investigate the perspectives of Wellspring Alberta members about the organization’s program delivery methods/modality (i.e., in-person vs online), to identify factors that are deemed to improve the experience of support through program participation and to identify barriers to access.

**Methodology or Methods**

This study incorporated mixed research methods that captured feedback from approximately 425 Wellspring Alberta members. Survey questions incorporated relevant subscales of the McGill Quality of Life Questionnaire and questions from the Measures of Processes of Care for Adults. Additional questions were asked to obtain information about barriers to participating in Wellspring Alberta programs. Interviews and focus group discussions were used to gather information about participants’ experiences with programs, program delivery methods and impacts and the challenges encountered.

**Impact on practice or results**

The analysis (in progress) has found common themes presenting Wellspring Alberta as a life-changing resource for individuals affected by cancer, with program participation, irrespective of modality, at Wellspring Alberta improving the quality of life among participants. Barriers and challenges to attending were identified including transportation to centers, technology challenges and program scheduling conflicts.

**Discussion or Conclusions**

The study outcomes will enhance Wellspring Alberta’s organizational operations, such as its organizational reach and program design/implementation, and reduce barriers to access, which should contribute to improvements in the members’ quality of life and increase equitable access. The study outcomes will directly benefit Wellspring Alberta members and indirectly benefit other organizations that provide psychosocial cancer support.

**53**

### 5.2. The Affective, Behavioral and Cognitive (ABC) Model for Graduate Student Integration into Community-Based Cancer-Supportive Organizations

Yoojung Kim ^1^ and Carmen Loiselle ^1,2,3^.

^1^  Division of Experimental Medicine, Faculty of Medicine and Health Sciences, McGill University, Montreal, Canada.
^2^  Department of Oncology, Faculty of Medicine and Health Sciences, McGill University, Montreal, Canada.
^3^  Segal Cancer Center, Montreal, Canada.

**Background/rationale or objectives/purpose**

Psychosocial care addresses sensitive issues that individuals affected by cancer often face. This poses a challenge for graduate students navigating such topics as they engage with patients, caregivers and family members while conducting psychosocial oncology research. Here, we illustrate a graduate student’s perspective underscoring the benefits of community engagement to support their research by learning about the lived experiences of individuals affected by cancer. The importance of adopting social and community contexts in cancer research is shared to support fellow students in the field.

**Methodology or Methods**

This graduate student is exposed to three cancer bereavement support programs offered by Hope and Cope, a volunteer-based community organization in Montreal, Quebec: Living with Loss, Mourning Walk and Mourning Cafe. Through journaling after each weekly encounter with bereaved caregivers and family members, the graduate student engages in self-reflection, documenting learned lessons and newly gained perspectives. Documented reflections are organized into categories.

**Impact on practice or results**

The journaling contents are categorized by exemplars of affective, behavioral and cognitive components. For instance, hearing program participants’ experiences allows emotions to emerge for impactful research to support individuals affected by cancer. Directly engaging with the community builds rapport, encourages active listening and acting upon any program content and/or delivery gaps. These experiences add richness to the understanding of psychological perspectives of grief.

**Discussion or Conclusions**

Sharing these experiences can lead to insightful discussions among graduate students within the field. Exercising all three (affective, behavioral and cognitive) approaches in psychosocial oncology research may encourage peer students towards community engagement, thereby supporting their learning as they navigate their studies.

**67**

### 5.3. A Five-Year Program’s Impact on Developing a Patient-Based Community at Medisprof Cancer Center

Camelia Moraru ^1,2^, Ruxandra Mara Oprea ^3^, Iulia Udrea ^3^, Camelia Toma ^4^ and Isai Popa ^4^.

^1^  Asociatia Medisprof, Cluj-Napoca, Romania.
^2^  Medisprof Cancer Center, Cluj-Napoca, Romania.
^3^  Asociatia Proiect A Zece, Cluj-Napoca, Romania.
^4^  Asociatia Pacientilor Voluntari, Cluj-Napoca, Romania.

**Background/rationale or objectives/purpose**

Based on an approach similar to the Optimal Healing Environment Model (Samueli Institute, 2004), the 10+ ArtProject and MEDISPROF Association’s five-year program addressed several patients’ needs beyond standard care—emotional and psychological support, art therapy and mindfulness-based counseling—in an attempt to contribute to their empowerment. The workshops’ results decorated medical spaces of the clinic in a pursuit of making them familiar and manageable. With three different types of support groups, the impact on the participants is evaluated by discussing the engaging and long-term motivational strategies for the patients, other participants (medical and auxiliary staff and caregivers) and organizers.

**Methodology or Methods**

Although the initial aim was space transformation, the interaction between patients and emotional regulation along the cancer continuum, the program impacted a larger category of direct and indirect beneficiaries that contributed to positive outcomes at a community level, on a broader scale than originally intended. The impact is analyzed based on insights gathered from 30 interviews with patients, organizers, artists involved, volunteers, staff from the clinic and caregivers.

**Impact on practice or results**

- Normalizing the condition and capability of patients to become actively involved in receiving support on other needs.
- Transforming the image of cancer for the participants involved.
- Developing more functional and adapted coping strategies.
- Open up the community towards embracing a medical reality and become part of the solution.

**Discussion or Conclusions**

Empowered patients are resources for their peers and all participants in support groups.

Changing the representation of cancer has a major impact at a community level, re-writing the fundamental coping strategies and life’s meaning.

**69**

### 5.4. A Survey of Patient, Family and Caregiver Needs to Inform Community-Based Cancer-Supportive Initiatives

Saima Ahmed ^1,2^, Yoojung Kim ^1^, Rick Simoneau ^2^, Suzanne O’Brien ^2^ and Carmen G. Loiselle ^1,2,3^.

^1^  McGill University, Montreal, Canada.
^2^  Hope and Cope, Montreal, Canada.
^3^  Segal Cancer Center, CIUSSS du Centre-Ouest-de l’Île-de Montréal, Montreal, Canada.

**Background/rationale or objectives/purpose**

Hope and Cope is a community-based cancer organization that assists individuals affected by cancer “regain a sense of control and wellbeing, reduce social isolation, and restore hope” by offering various initiatives (workshops, support groups and activities) in multiple modalities (in-person, virtual and hybrid). A 2024 Quality Improvement Survey was the first step in a co-design approach to involve patients, family members and caregivers in identifying needs and preferences on current and future program offerings.

**Methodology or Methods**

Patients, family members and caregivers (N = 200) in oncology in Montreal, Quebec, are invited to complete the survey. Links and QR codes to the survey are shared through the Hope and Cope newsletter, social media and posters. A paper version of the survey is available at Hope and Cope reception desks. Respondents are asked to answer 11 questions including their preferences for activity times and modalities, support group types, as well as which cancer-specific/treatment-related, community support and physical wellness topics they are most interested in.

**Impact on practice or results**

Data collection and analysis is to be completed by March 2024. A focus will be on the overall topics of interest according to the participant profiles by cancer type, stage, sex/gender and age.

**Discussion or Conclusions**

This quality improvement survey provides a promising way to ensure programs are co-created with the needs and preferences of participants in mind.

**145**

### 5.5. The Importance of Peer Support for the Blood Cancer Community: Personalized Support from Diagnosis through Life after Cancer

Desiree Naylor.
Leukemia and Lymphoma Society of Canada, Calgary, Canada.

**Background/rationale or objectives/purpose**

This poster will describe the results of our literature review on the impact of peer support on health outcomes. In 2022, the Leukemia and Lymphoma Society of Canada relaunched its peer support program with a rigorous process for volunteer recruitment and training, risk management and ongoing evaluations. More virtual support groups were offered during the pandemic as another way to connect with peers, with an impressive participation rate and excellent evaluation.

People affected by blood cancer can feel isolated and even misunderstood by family and friends during their cancer experience. When this happens, it can be destabilizing. Communicating with someone who has experienced a similar cancer diagnosis can bring a level of understanding. Receiving a blood cancer diagnosis is a traumatic experience and some people may find it difficult to believe in the future. Talking to someone who has a good quality of life makes the future possible and gives hope.

Many cancer survivors feel the need to give back. Peer support is one way to keep them engaged in their community and give meaning to the cancer experience.

**Methodology or Methods**

Needs assessment and literature review.

**Impact on practice or Results**

Peer support is a powerful and effective coping strategy for those who feel hopelessness and isolation during their cancer experience. Knowing that others have had similar experiences, and that they can help, may make all the difference in how someone lives through the challenge of cancer.

**Discussion or Conclusions**

Peer support can improve health outcomes for those affected by blood cancer.

People affected by a blood cancer:

- Require frequent and long-term follow-up care.
- Have to manage symptoms and side-effects at home.
- Have unique needs and the experience is different from people with solid tumors.
- Have higher levels of anxiety.
- Have higher levels of depression.

## 6. Final Category: E. Complementary and Integrative Cancer Care

**2**

### 6.1. The Role of Hardiness on the Psychological Wellbeing of Caregivers of Patients with Cancer in Uganda: A Hardiness Perspective

Rachel Kansiime ^1,2,3^, Milton Mutto ^4^, Jackson Orem ^2^, Godfrey Zari Rukundo ^5^ and Simon Kizito ^6^.

^1^  Joint Clinical Research Center, Kampala, Uganda.
^2^  Uganda Cancer Institute, Kampala, Uganda.
^3^  Mulago National Specialized Hospital, Kampala, Uganda.
^4^  Pincer Research and Training Institute, Kampala, Uganda.
^5^  Mbarara University of Science and Technology, Mbarara, Uganda.
^6^  Makerere University, Kampala, Uganda.

**Background/rationale or objectives/purpose**

Caring for patients with cancer places substantial emotional and psychological strain on caregivers, particularly in resource-limited settings such as Uganda. This study investigated the role of hardiness as a protective factor in mitigating the psychological distress faced by caregivers of patients with cancer in Uganda.

Hardiness, characterized by a combination of commitment, control and challenge, is proposed as a resilience factor that may influence how caregivers perceive and cope with the stressors associated with providing care for a loved one with cancer. This research aimed to explore the role hardiness plays on the psychological well-being of caregivers, offering insights into potential avenues for targeted psychosocial support.

**Methodology or Methods**

A cross-sectional study was carried out at the Uganda Cancer Institute and Mbarara Regional Referral Hospital between June 2019 and December 2021. Participants were informal adult caregivers of patients who had been diagnosed with cancer at the two sites. Their socio-demographic attributes were recorded using a questionnaire that was developed while scores on hardiness and psychological well-being were measured using the adjusted hardiness scale and adjusted the general health questionnaire.

The tools were subjected to exploratory factor analysis. Composite indices were generated and used to determine quantitative measures of psychological well-being. The derived scores were then used in the subsequent analyses of the effect of hardiness on psychological well-being using Stata 14 software.

**Impact on practice or results**

The results inform the development of targeted interventions to enhance hardiness, thus promoting the psychological well-being of cancer caregivers in resource-limited settings.

**Discussion or Conclusions**

Hardiness affects caregivers’ psychological well-being and needs to be enhanced.

**25**

### 6.2. Access to Medical and Integrative Care for Patients with Cancer Residing in Rural Saskatchewan: A Qualitative Study in Progress

Mohamad Baydoun.
University of Regina, Regina, Canada.

**Background/rationale or objectives/purpose**

As the first step in a program of research exploring and intervening on the health-related needs and concerns of individuals with cancer in rural Saskatchewan, this qualitative study aims to explore the views of rural Saskatchewanians on accessing medical and integrative care.

**Methodology or Methods**

This qualitative research employs an interpretive descriptive methodology. Eligibility criteria include (1) a current/active cancer diagnosis, (2) age 18+ years and (3) residency in a rural community in Saskatchewan. Verbal consent is confirmed and digitally recorded for a semi-structured videoconference 60 min interview at a mutually agreeable date and time.

**Impact on practice or results**

As of February 2024, we have successfully conducted four interviews with participants diagnosed with cancer residing in rural Saskatchewan. We are employing a concurrent approach to data collection and analysis, aiming to complete this phase by the end of 2024 with an anticipated total of 20 participants. The interviews are exploring the experiences of participants as they navigate the healthcare system, specifically focusing on their access to medical and integrative oncology services and identifying any obstacles they face in receiving care.

**Discussion or Conclusions**

The results can help us address the needs of patients and achieve high-quality and comprehensive cancer care for all Saskatchewanians, wherever they live. The rapid translation of our results to clinicians can improve cancer care delivery in real time and provide a foundation for future innovation in cancer care in Saskatchewan.

**36**

### 6.3. Fear. Facts. Fantasy. Faith.

Eleanor Deckert.
Freelance Columnist, Instructor, Author, Avola, BC, Canada.

**Background/rationale or objectives/purpose**

All of the most advanced scientific interventions in the world could not save my life if I were too full of fear to accept treatment.

**Methodology or Methods**

Cancer means death.

Treatment will save my life.

I am not afraid to die. I am afraid of treatment: Cut. Burn. Poison.

I was on the verge of refusing treatment.

Counseling saved my life.

**Impact on practice or results**

The radiation appointment takes care of my BODY in a few minutes.

How will I take care of the rest of my SELF for the other twenty-three-and-a-half hours every day? HEART? SPIRIT? MIND?

I needed someplace safe, comforting, more solid than all of this turmoil. I needed to talk. Who would listen? I needed skills to quiet the storm, address the underlying childhood fear, logistics and resources. These were provided by my counsellor. You, like her, will save lives.

**Discussion or Conclusions**

FEAR.

From the moment my doctor said, ‘cancer’, fear grew inside me.

I could not taste my food and could hardly swallow. I could not listen, read, remember or plan.

I could not sleep. It was not safe for me to drive.

FACTS.

I knew I needed reliable facts, logical explanations. But, I found contradiction, controversy, confusion. I asked simple questions: Do we know how cancer starts? Can it be Prevented? Removed? Stopped? Every answer was “Maybe”.

FAITH.

Faith had been the central core of my life, but suddenly it felt fake.

FANTASY.

I imagined myself as a falconer. My falcon would remove the deadly eels from the marsh, restoring it to a healthy balance.

**43**

### 6.4. KST for Cancer: Exploring a Kundalini Somatic Therapy (KST) Intervention for Post-Traumatic Growth in People with Cancer

Julie Deleemans ^1^, Hanna Conradi ^1^ and Linda Carlson ^1^.

^1^  University of Calgary Cumming School of Medicine, Calgary, Canada.

**Background/rationale or objectives/purpose**

Kundalini Somatic Therapy (KST) is a novel approach that uses somatic strategies and works with an individuals’ energy to support the process of Kundalini Awakening (KA). Spontaneous KA experiences reportedly expand one’s consciousness, which can result in long-lasting therapeutic changes. However, most KA experiences occur spontaneously; it is unknown if KST can reliably induce these experiences. Cancer is associated with high levels of distress and trauma, which can adversely impact patients’ physical and emotional health. This pilot proof-of-concept study aims to determine whether a KST intervention can (i) elicit a KA experience and altered states of consciousness, (ii) improve psychosocial health and (iii) support post-traumatic growth.

**Methodology or Methods**

Using a mixed-methods, single-arm design, we aim to recruit N = 30 people, aged ≥ 18 years, with a current or previous cancer diagnosis scoring ≥ 4 on the distress thermometer. The intervention consists of 7 weekly 2 h in-person group (n = 10) sessions. Validated patient-reported outcome measures of distress, trauma, depression, anxiety, post-traumatic growth, mystical experiences, altered states of consciousness, and kundalini awakening will be collected at baseline, week 7, and at a 3-month follow-up. Qualitative, semi-structured interviews will be conducted with 12 diverse participants post-intervention.

**Impact on practice or results**

Quantitative data will be analyzed using descriptive statistics and paired-sample t-tests. A thematic inductive analysis will be applied to the qualitative data. Preliminary results from cohort 1 will be presented at the conference.

**Discussion or Conclusions**

This first-of-its-kind study will explore a novel biofield therapy intervention to support people with cancer. If successful, this intervention may yield evidence for effective, non-invasive opportunities for fostering healing and post-traumatic growth.

**123**

### 6.5. Society for Integrative Oncology—American Society of Clinical Oncology Joint Guideline on Integrative Therapies for Symptoms of Anxiety and Depression in Adults with Cancer

Linda Carlson ^1^ and SIO-ASCO Anxiety–Depression Guidelines Panel Members ^2^.

^1^  University of Calgary, Calgary, Canada.
^2^  Society for Integrative Oncology, Washington, DC, USA.

**Background/rationale or objectives/purpose**

The Society for Integrative Oncology (SIO) partnered with the American society of Clinical Oncology (ASCO) to produce a series of evidence-based guidelines for the treatment of cancer-related symptoms and side-effects. The most recent guideline was intended to provide evidence-based recommendations to health care providers on integrative approaches to managing anxiety and depression symptoms in adults with cancer.

**Methodology or Methods**

SIO and ASCO convened an interdisciplinary expert panel to review the literature, which included systematic reviews, meta-analyses and randomized controlled trials published from 1990 through to 2023. The outcomes of interest included anxiety or depression symptoms as measured by validated psychometric tools. Expert panel members used this evidence and informal consensus with the Guidelines into Decision Support methodology to develop evidence-based guideline recommendations.

**Impact on practice or results**

The literature search identified 110 relevant studies (30 systematic reviews and 80 randomized controlled trials), which included over 400 individual RCTs to inform the evidence base for this guideline. Recommendations were made for mindfulness-based interventions (MBIs), yoga, relaxation, music therapy, reflexology and aromatherapy (using inhalation) for treating symptoms of anxiety during active treatment and MBIs, yoga, acupuncture, tai chi and/or qigong and reflexology for treating anxiety symptoms after cancer treatment. For depression symptoms, MBIs, yoga, music therapy, relaxation and reflexology were recommended during treatment and MBIs, yoga and tai chi and/or qigong were recommended post-treatment.

**Discussion or Conclusions**

While several approaches appear effective, limitations include the assessment of the risk of bias, the nonstandardization of therapies, a lack of diversity in the study samples, a lack of active control conditions and an inability to provide conclusive recommendations for some popular therapies.

**129**

### 6.6. The Integrative Oncology (IO) Clinic Study: Assessment of Psychological Outcomes in Clinic Participants

Tina Nguyen ^1^, Safiya Karim ^1,2^ and Linda E. Carlson ^1^.

^1^  University of Calgary, Calgary, Canada.
^2^  Alberta Health Services, Calgary, Canada.

**Background/rationale or objectives/purpose**

Combining the use of complementary therapies (CT) with conventional treatment in an integrative oncology (IO) model has shown improvements in outcomes such as quality of life, psychological functioning and other side-effects in people with cancer. Many patients are self-navigating CT use without healthcare provider (HCP) guidance; this has the potential to do harm or waste patient resources. Problematically, HCPs have reported limited knowledge about CTs in general and how to incorporate them into patients’ care. The IO clinic at the Tom Baker Cancer Center was launched in 2022 to meet this need.

**Methodology or Methods**

High-needs patients undergoing conventional cancer treatments were referred to the clinic by HCPs. Participants consulted with a medical oncologist trained in IO to come up with a CT treatment plan. The Edmonton Symptom Assessment Scale-r was used to measure patient-reported outcomes at the baseline and first follow-up appointment. Paired sample t-tests were performed to compare the two timepoints.

**Impact on practice or results**

Preliminary data from 27 participants in the IO Clinic Study were analyzed. The mean patient age was 55.45 years (SD = 16.58) and 100% were female. Significant changes were observed for pain (t_26_ = 2.760, *p* = 0.010), tiredness (t_26_ = 2.935, *p* = 0.007), drowsiness (t_26_ = 3.002, *p* = 0.006) and anxiety (t_26_ = 2.498, *p* = 0.019) between the two timepoints.

**Discussion or Conclusions**

IO clinic attendance was associated with improvements in patients’ psychosocial outcomes. These preliminary results suggest that having a clinic where patients can receive personalized support on how to integrate CT into their conventional treatment may help better support cancer patients and survivors.

## 7. Final Category: F. Digital Health and Cancer Care

**45**

### 7.1. Predicting Psychosocial Needs Using Natural Language Processing

John-Jose Nunez ^1,2^, Bonnie Leung ^2^, Cheryl Ho ^1,2^, Raymond Ng ^1^ and Alan Bates ^1,2^.

^1^  UBC, Vancouver, Canada.
^2^  BC Cancer, Vancouver, Canada.

**Background/rationale or objectives/purpose**

Cancer patients often have unmet psychosocial needs. While sometimes a result of a lack of resources, the inefficient identification of needs also contributes. Prior work has shown that oncologists may only identify about a third of patients with significant psychosocial distress, for example. We aimed to test Natural Language Processing (NLP), a form of artificial intelligence, as a tool for using data recorded during clinical interactions to identify a need for psychosocial services.

**Methodology or Methods**

We analyzed the text of 47,625 oncologists’ new assessment notes using both traditional and neural NLP. The documents represented 80% of all new assessments by medical and radiation oncologists at BC Cancer between April 2011 and December 2016. We assessed the models’ ability to predict a referral to counselling/social work and psychiatry within one year using balanced accuracy (BA) and a receiver-operating characteristic area-under-the-curve (AUC).

**Impact on practice or results**

The best models achieved very good performance in predicting a referral to psychiatry (BA 76.1%, AUC 0.815) and counselling/social work (BA 68.8%, AUC 0.752) services. Mental health-related tokens (word stems) such as “depress” as well as demographic factors such as occupational status were important in predicting both psychiatry and counselling. Tokens associated with a patient’s cancer were more often used in predicting psychiatry. The models also demonstrated the ability to use factors such as family history of cancer and symptoms suggesting use of steroids to predict the need for psychosocial supports.

**Discussion or Conclusions**

NLP shows promise as a tool for the early identification of psychosocial needs using only the oncology new assessment note.

**55**

### 7.2. Preparing to Transition from Pediatric to Adult Care: A Web Resource Page for Young People Diagnosed with Cancer

Nadège Gendron Granger ^1,2^, Marika Monarque ^2,3^, Carole Provost ^4^, Wendy Louis-Delsoin ^1^, Nathalie Labonté ^1^, Élodie Bergeron ^5^, Zeev Rosberger ^6,7,8^, Argerie Tsimicalis ^9^, Serge Sultan ^2,10^ and Leandra Desjardins ^1,2^.

^1^  Pediatric Oncology Neuro Social Lab of Sainte-Justine University Hospital Center (CHUSJ), Montreal, Canada.
^2^  Department of Psychology of University of Montreal, Montreal, Canada.
^3^  Pediatric Oncology Neuro Social Lab of Sainte-Justine University Hospital Center (CHUSJ), Montréal, Canada.
^4^  Sainte-Justine University Hospital Center (CHUSJ), Montreal, Canada
^5^  Leucan, Montreal, Canada.
^6^  Lady Davis Institute for Medical Research (LDI) of Jewish General Hospital, Montreal, Canada.
^7^  Department of Oncology of McGill University, Montreal, Canada.
^8^  Department of Psychology and Psychiatry of McGill University, Montreal, Canada.
^9^  Ingram School of Nursing of McGill University, Montreal, Canada.
^10^  Psycho-Oncology Center of Sainte-Justine University Hospital Center (CHUSJ), Montreal, Canada.

**Background/rationale or objectives/purpose**

Individuals diagnosed with a pediatric cancer require lifelong medical follow-up, but are often unprepared for the transition to adult healthcare settings. There are limited transition web-based resources available, particularly in French, to help support this process. This study aimed to create a bilingual transition resource website informed by youths treated for pediatric cancer and their parents.

**Methodology or Methods**

We conducted a longitudinal study using a mixed approach at CHU Sainte-Justine. We recruited adolescents previously diagnosed with cancer, off treatment, aged 14 to 18 (focus groups n = 13; survey n = 44), and caregivers (focus groups n = 5; survey n = 46) to participate. Surveys assessed knowledge of existing transition resources. Transition webpage content was based on review of transition resources and collaboration with a patient partner. Qualitative focus groups gathered feedback on positive and negative aspects of the webpage and preferences regarding content and format.

**Impact on practice or results**

Survey data revealed a low awareness among youths and parents of websites providing transition resources (e.g., Transition Hub 0%, Parachute 16 and 17%). Leucan was the most well-known website (91 and 93%). Focus groups indicated mental health resources were critical for youths and that dedicated transition content was needed for parents.

**Discussion or Conclusions**

This study is part of a larger project aiming to implement transition readiness screening in pediatric oncology follow-up care in Quebec. We have now included the QR code of the revised and co-created website to the screening questionnaire and hope to see a positive impact of screening and resource guidance on youths’ transition skills.

**63**

### 7.3. A Community Engagement Event during the Development of a Mobile Health Intervention for Survivors of Childhood Cancer

Brianna Henry ^1^, Rachelle Drummond ^1^, Iqra Rahamatullah ^1^, Caitlin Forbes ^1^, Sharon Hou ^1,2^ and Fiona Schulte ^1,3^.

^1^  Department of Oncology, Cumming School of Medicine, University of Calgary, Calgary, Canada.
^2^  Department of Psychology, BC Children’s Hospital, Vancouver, Canada.
^3^  Alberta Children’s Hospital, Calgary, Canada.

**Background/rationale or objectives/purpose**

Although survivors of childhood cancer require long-term follow-up (LTFU) care that is evidence- and exposure-based, fewer than 50% attend LTFU care. Innovative and accessible approaches that engage survivors are urgently needed. This study describes a community engagement event designed to verify previously identified components and co-develop additional components for inclusion in mobile health (mHealth) intervention as part of a larger, multi-phase project.

**Methodology or Methods**

A total of 28 participants, including survivors (n = 10), caregivers (n = 2), clinicians (n = 6) and researchers (n = 10), were recruited via LTFU clinics and community settings to participate in a half-day virtual community engagement event. Participants engaged in small and large group discussions co-facilitated by 2 researchers and 2 patient partners using open-ended questions facilitated through Mentimeter, Google Jamboard and Zoom. A graphic recording captured themes during the event and an online satisfaction survey (Likert scale 1–5) was conducted one week afterward.

**Impact on practice or results**

Participants endorsed the accuracy, representativeness and resonance of components to be included in a mHealth intervention. Discussions highlighted a desire to include family members as platform users and the importance of privacy considerations. Participants responded positively to the graphic recording. Participants who completed the satisfaction survey (n = 8) strongly agreed that they felt their views were heard (*M* = 5.00; *SD* = 0.00) and they were able to discuss them freely (*M* = 4.71; *SD* = 0.45).

**Discussion or Conclusions**

The community engagement event achieved its objectives of co-designing a mHealth intervention and gathering feedback on study results. This event was a critical step in validating preceding work and will guide the subsequent phase of developing an mHealth intervention prototype.

**116**

### 7.4. Stakeholder Codesign of a Patient-Portal-Based Peer-Support Service for People Diagnosed with Cancer

Andréa Maria Laizner ^1,2,3^, Loes Knaapen ^1^, Luc Galarneau ^1^, Susie Judd ^1^, Tristan Williams ^1^, Kelly Agnew ^1^, Ridhi Mittal ^1^ and John Kildea ^1,3^.

^1^  The Research Institute of the McGill University Health Center, Montreal, Canada.
^2^  McGill University Health Center, Montreal, Canada.
^3^  McGill University, Montreal, Canada.

**Background/rationale or objectives/purpose**

Cancer patients navigate many medical, logistical and psychosocial challenges that can cause distress. Cancer-related distress can be reduced through peer support. A new cancer patient can obtain some foresight through the hindsight of a previous patient. Therefore, our goal is to provide a mechanism via a patient portal app to match current patients with previous patients for the purpose of facilitating in-person peer support (OncoBuddy) and sharing relevant written advice (OncoConseil).

**Methodology or Methods**

We are co-designing, with cancer patients and other stakeholders, an artificial intelligence-driven approach to incorporate peer-matching into the Opal patient portal app (opalmedapps.com). Eight cancer patient users of Opal completed semi-structured interviews that were transcribed verbatim and content categorized into themes.

**Impact on practice or results**

The stakeholder codesign process was invaluable. The interviews led to changing the name of the proposed peer support service from OncoBuddy and OncoConseil to the more inclusive OpalBuddy and OpalTips, respectively. Patients wanted access to support from a peer mentor across the illness trajectory and life-stage events. Ideal peer mentor characteristics depended upon, among others, the issue/concern, accessibility, diagnosis and treatment, age and life stage, being a good listener and able to provide practical information and support, but relative importance varied across patients. The Opal development team created mockup solutions based upon the possible criteria.

**Discussion or Conclusions**

Patient participants highly valued a peer-support service offered in a patient portal and were willing to engage in stakeholder co-designs to create such a service. In terms of peer matching, the ‘ideal mentor’ does not exist, but needs to be based on patient preferences.

**137**

### 7.5. A Multi-Phased, Patient-Oriented Approach to Co-Design an mHealth Intervention for Young Adult Survivors of Childhood Cancer to Enhance Their Follow-Up Care

Sharon Hou ^1,2^, Brianna Henry ^1^, Rachelle Drummon ^1^, Holly Wright ^1^, Kyle Mendonça ^1^, Caitlin Forbes ^1^ and Fiona Schulte ^1^.

^1^  University of Calgary, Calgary, Canada.
^2^  University of British Columbia, Vancouver, Canada.

**Background/rationale or objectives/purpose**

Survivors of childhood cancer are at risk of medical, psychological and social late effects. To screen for these risks, the receipt of consistent and cancer-specific follow-up care is critical. However, less than 50% of survivors attend their follow-up care and only 35% of them recognize that they could have a serious health problem. The use of mobile health (mHealth) is a promising form of intervention to educate, connect and empower survivors on the importance of their follow-up care.

**Methodology or Methods**

This study employed patient-oriented research methods and was grounded in an established conceptual framework for co-design of mHealth. Qualitative data were gathered as part of a larger, multi-phased project regarding survivors of childhood cancer and their follow-up care experiences. Data collected were analyzed using reflexive thematic analysis and verified through member checking techniques.

**Impact on practice or results**

We co-facilitated five virtual focus groups with 22 survivors of childhood cancer (mean age = 29.19 years, *SD* = 4.78) with patient partners. We conducted telephone interviews with seven healthcare providers. The participants reported residing from five provinces across Canada. The participants identified five priority areas to be included in an mHealth platform: connections, education and information, engagement, personalization and resources.

**Discussion or Conclusions**

The results from the current study provided the necessary foundation to progress in intervention development. The next step of this multi-phased project is to pilot an innovative and accessible mHealth intervention prototype based on the core components identified. Ultimately, outcomes are intended to enhance the follow-up care and long-term outcomes of survivors of childhood cancer.

## 8. Final Category: G. Exercise/Pre-Habilitation and Rehabilitation in Cancer

**9**

### 8.1. Understanding How Clinical Conversations of Physical Activity Are Incorporated into Care for Non-Curative Cancer Patients

Jodi Langley ^1,2^, Allison Kearley ^3^, Grace Warner ^1^, Christine Cassidy ^1^, Robin Urquhart ^1,2,4^, Mary MacNeil ^1,4^ and Melanie Keats ^1,2,4^.

^1^  Dalhousie University, Halifax, Canada.
^2^  Beatrice Hunter Cancer Research Institute, Halifax, Canada.
^3^  Patient Partner, New Glasgow, Canada.
^4^  Nova Scotia Health, Halifax, Canada.

**Background/rationale or objectives/purpose**

Oncology Care Providers (OCPs) have a trusted relationship with patients, which puts them in a good position to engage in physical activity (PA) discussions at different timepoints throughout diagnosis and treatment. Furthermore, their understanding of individual patient limitations and symptom burden make them uniquely qualified to provide tailored PA recommendations. The objective of this study is to understand the perspectives of OCPs and knowledge users (e.g., managers) on how clinical conversations of PA can be incorporated into care for those living with non-curative cancer.

**Methodology or Methods**

This is a qualitative description interview study to better understand how clinicians engage in physical activity discussions with their patients.

**Impact on practice or results**

Sixteen in-depth interviews took place with 11 OCPs and 5 knowledge users (e.g., health administrators, senior leadership). The following themes were identified: (1) OCPs give vague advice regarding PA with their patients, (2) deciding how and when patients are ready to discuss PA takes a personal toll on OCPs and (3) OCP champions of PA are key to answering “on the fly” questions.

**Discussion or Conclusions**

PA discussions are an important aspect to consider during clinical care. Certain OCPs may have a greater history of engaging in PA themselves and knowledge surrounding the benefits of PA. Therefore, they are more likely to engage more frequently in PA discussion. **Clinical Implications**: there is a need to support clinicians and give them the tools to engage in conversations surrounding PA at all stages of cancer diagnosis and treatment and, if needed, refer patients to further PA supports.

**38**

### 8.2. Predictors of Adherence to an Exercise Oncology Intervention for Individuals Living in Rural and Remote Communities

Daniel Sibley ^1^, Chad Wagoner ^2^, Julianna Dreger ^2^, Melanie R. Keats ^3^, Jodi Langley ^3^, Margaret L. McNeely ^4^, Colleen Cuthbert ^2^, Linda Trinh ^1^, Daniel Santa Mina ^1^ and S. Nicole Culos-Reed ^2^.

^1^  University of Toronto, Toronto, Canada.
^2^  University of Calgary, Calgary, Canada.
^3^  Dalhousie University, Halifax, Canada.
^4^  University of Alberta, Edmonton, Canada.

**Background/rationale or objectives/purpose**

Exercise interventions have been shown to mitigate the health-related burden of cancer in a dose-dependent manner, implying that higher rates of adherence are associated with increased effectiveness. The purpose of this preliminary analysis was to examine the baseline predictors of adherence to an exercise oncology intervention.

**Methodology or Methods**

EXercise for Cancer to Enhance Living well (EXCEL) is an ongoing, pan-Canadian, 12-week rural and remote exercise oncology study. A binomial logistic regression was performed to determine the predictors of adhering to EXCEL. Predictor variables were demographic (age, sex, marital status, education and smoking), program delivery (online vs. in-person), clinical (cancer type, metastasis and treatment status) and patient-reported (Functional Assessment of Cancer Therapy—General [FACT-G], exercise self-efficacy, Edmonton Symptom Assessment System [ESAS]). The outcome variable was adherence calculated as a percentage of exercise classes attended and categorized as low (≤50%) versus high (>75%).

**Impact on practice or results**

In total, 1137 participants were included and were mostly female (72%), on treatment (68%), married (57%), attended virtual classes (85%) and had breast cancer (47%), with a mean age of 58 years. The mean adherence was 77% and 276 and 505 participants had low and high adherence, respectively. Adherence data were not available for 356 participants. The predictors of high adherence included age (OR [CI] = 1.04 [1.02, 1.05]), marital status (OR [CI] = 0.338 [0.11, 0.99]) and FACT-G social well-being (OR [CI] = 0.947, [0.91, 0.99]; all *p* < 0.05).

**Discussion or Conclusions**

This preliminary analysis outlines the predictors of adhering to an exercise oncology intervention and highlights the need for greater adherence supports for individuals who are younger, separated and report greater social well-being.

**78**

### 8.3. Examining Baseline Characteristics and Exercise Adherence in Participants with Metastatic Cancer Enrolled in the Community-Based Alberta Cancer Exercise Program: A Secondary Analysis

Shirin M. Shallwani ^1,2^, Anil Abraham Joy ^1,3^, Edith Pituskin ^1^, Michelle Audoin ^4^, S. Nicole Culos-Reed ^5^ and Margaret L. McNeely ^1,3^.

^1^  University of Alberta, Edmonton, Canada.
^2^  McGill Lymphedema Research Program, Montreal, Canada.
^3^  Cross Cancer Institute, Edmonton, Canada.
^4^  Toronto, Canada.
^5^  University of Calgary, Calgary, Canada.

**Background/rationale or objectives/purpose**

Despite the psychosocial and fitness benefits of exercise, individuals with metastatic cancer are particularly underserved in terms of appropriate exercise support. They face challenging barriers to exercise participation, including symptoms of fatigue, mental health concerns and risks of injury (e.g., bone fracture). Further research is needed on how to tailor and implement evidence-based exercise programs in real-world settings as part of supportive cancer care for people with metastatic cancer.

The objective of our study is to describe baseline characteristics and exercise adherence in participants with metastatic cancer enrolled in the Alberta Cancer Exercise (ACE) program.

**Methodology or Methods**

The ACE study examines the effectiveness and implementation of a 12-week community-based exercise program for people with cancer. We conducted a secondary data analysis of a subgroup of 310 participants from the ACE study with the confirmed presence of metastatic cancer. Participant characteristics at the baseline were analyzed descriptively. Study completion and program attendance rates were tracked at 12 weeks.

**Impact on practice or results**

The participants’ mean age was 57.8 years. Common cancer types included breast (33%), genitourinary (16%), digestive (14%) and lung (11%). Most participants reported experiencing fatigue (90%) and being sedentary/insufficiently active (79%) at the baseline. At 12 weeks, 88.3% of participants completed the follow-up assessment and mean exercise adherence was 72.8%.

**Discussion or Conclusions**

Examining the characteristics of participants with metastatic cancer can help us understand who may be able to participate in community-based exercise programs as part of supportive cancer care. This research can inform the design of exercise strategies that better meet the unique needs of this underserved cancer population.

**85**

### 8.4. Protocol for a Multiphasic Multimodal Exercise Prehabilitation Program for Individuals Receiving Allogeneic Stem Cell Transplant

Jocelyn Cannon ^1^, Julia Daun ^1^, Chad Wagoner ^1,2,3^, Jessica Danyluk ^1^, David Langelier ^4,5^, George Francis ^4^, Sarah Perry ^5^ and Nicole Culos-Reed ^1,5,6^.

^1^  Faculty of Kinesiology, University of Calgary, Calgary, Canada.
^2^  Department of Community Health Sciences, Cumming School of Medicine, University of Calgary, Calgary, Canada.
^3^  Ohlson Research Initiative, Cumming School of Medicine, University of Calgary, Calgary, Canada.
^4^  Physical Medicine and Rehabilitation, Department of Clinical Neurosciences, Cumming School of Medicine, University of Calgary, Calgary, Canada.
^5^  Department of Oncology, Cumming School of Medicine, University of Calgary, Calgary, Canada.
^6^  Department of Psychosocial Resources, Tom Baker Cancer Center, Alberta Health Services, Calgary, Canada.

**Background/rationale or objectives/purpose**

Allogeneic stem cell transplantation (ASCT) is a lifesaving treatment for acute leukemia which is associated with decreased physical function and quality of life. Exercise may provide a sense of control and improve both physical and psychosocial indices, yet its delivery is a challenge in the ASCT patient population. The Alberta Cancer Exercise–Hematopoietic Stem Cell Transplant (ACE-HSCT) study is thus examining the feasibility of and patient outcomes from multiphasic, multimodal prehabilitation exercise intervention.

**Methodology or Methods**

Adults ≥ 18 years diagnosed with acute leukemia who are eligible for ASCT are referred by the clinical team. Participants receive weekly 10–45 min tailored exercise sessions in-person or online. Sessions are delivered by Clinical Exercise Physiologists and are embedded in a positive motivational framework with health coaching to support behavior change. Additionally, participants are offered twice-weekly 10–15 min yoga sessions with a certified yoga instructor. Feasibility measures include participant satisfaction, recruitment, enrollment, measurement completion rates, program adherence and safety. Assessments of physical function and patient-reported outcomes occur at baseline, one-week pre-transplant, one-week post-hospital discharge and 12-weeks post-discharge. Objective physical activity is assessed across all timepoints.

**Impact on practice or results**

Recruitment opened in October 2024 and will be ongoing until 2025. To date, 10 participants have enrolled into the study. The yoga protocol will begin in Spring 2024.

**Discussion or Conclusions**

The trial will assess the feasibility and potential impact of exercise prehabilitation in standard cancer care for ASCT. ACE-HSCT may offer participants an opportunity to enhance the patient’s self-efficacy and sense of control over their wellness during an intense period of focus on their illness.

**100**

### 8.5. Exploring the Implementation of an Online Yoga Trial for Young Adults Affected by Cancer

Emma McLaughlin ^1^, S Nicole Culos-Reed ^1,2,3^, Hannah Cripps ^1^, Julianna Dreger ^1^ and Amanda Wurz ^1,4^.

^1^  Faculty of Kinesiology, University of Calgary, Calgary, Canada.
^2^  Department of Oncology, Cumming School of Medicine, University of Calgary, Calgary, Canada.
^3^  Department of Psychosocial Resources, Tom Baker Cancer Center, Cancer Care, Alberta Health Services, Calgary, Canada.
^4^  School of Kinesiology, University of the Fraser Valley, Chilliwack, Canada.

**Background/rationale or objectives/purpose**

Yoga is safe and beneficial for individuals living with and beyond cancer. Yet, there is little research exploring the role of yoga for young adults (YAs) affected by cancer, and little research has explored the online delivery of yoga with this cohort. To address this, we built on our pilot yoga work and developed an online, Canada-wide yoga intervention for YAs affected by cancer.

**Methodology or Methods**

Since 09/2021, we have been conducting a hybrid effectiveness-implementation trial to assess the yoga intervention. YAs diagnosed with cancer between the ages of 18 and 39 years old are offered 60 min yoga classes 2×/week online. Specific implementation markers include reach, feasibility [i.e., retention, attendance, percentage of missing data, time and cost of delivery], fidelity and adverse events.

**Impact on practice or results**

As of 09/2023, 94 YAs have expressed interest, 66 have consented and enrolled, 42 have completed the intervention and 38 have completed the post-intervention assessments. Retention to the trial is 90% and, on average, 17 of 24 (71%) classes in each session have been attended. Missing data to-date are <5% and over 200 h have been allocated for intervention training and delivery, costing upwards of CAD 8400. No adverse events have been reported.

**Discussion or Conclusions**

These data have provided invaluable information to guide ongoing trial delivery, as results are reviewed during regular quality improvement cycles every 6 months. Further, implementation data findings will inform plans for the sustainable implementation of future community-based online or in-person yoga interventions for YAs affected by cancer.

**115**

### 8.6. The Role of Patient and Caregiver Voices in an Online Physical Activity Trial for Children and Adolescents Affected by Cancer

Emma McLaughlin ^1^, Mira Penney ^2^, Bridget Penney ^2^, Laura Wich ^2^, Hannah Cripps ^1^, Georgia Kaluznick ^1^, Carolina Chamorro-Viña ^1,3^, Sara Fisher ^4^, S Nicole Culos-Reed ^1,5,6^ and Amanda Wurz ^1,7^.

^1^  Faculty of Kinesiology, University of Calgary, Calgary, Canada.
^2^  Participant Advisory Board, Canada.
^3^  Kids Cancer Care Foundation of Alberta, Calgary, Canada.
^4^  Stollery Children’s Hospital, Edmonton, Canada.
^5^  Department of Oncology, Cumming School of Medicine, University of Calgary, Calgary, Canada.
^6^  Department of Psychosocial Resources, Tom Baker Cancer Center, Cancer Care, Alberta Health Services, Calgary, Canada.
^7^  School of Kinesiology, University of the Fraser Valley, Chilliwack, Canada.

**Background/rationale or objectives/purpose**

Patient-oriented research (POR) is conducted to ensure the research question and process focuses on priorities and outcomes that are relevant to patients. A goal of POR is to implement findings to improve healthcare outcomes and patients’ quality of life. We used a POR approach to develop an online physical activity (PA) intervention and a hybrid effectiveness-implementation trial for children and adolescents affected by cancer (i.e., IMPACT).

**Methodology or Methods**

Currently, two patients and two caregivers serve on the IMPACT Advisory Board (IAB) alongside trial staff, clinicians and community partners to ensure the family/caregiver and patient voices inform the vision and mission of IMPACT. The IAB meets online quarterly to address relevant IMPACT research issues and processes (e.g., recruitment issues, engagement of clinicians) and to provide input on the overall delivery of the PA intervention and implementation of outreach strategies. IAB members can share feedback at any time via email or phone calls.

**Impact on practice or results**

The IAB was formed in 08/2023 and through online meetings (n = 3) and email correspondence, to-date they have addressed streamlining recruitment materials and sharing patient and caregiver testimonials in outreach materials. Outreach initiatives supported by the IAB include building awareness through media days and sharing testimonials for grant applications. The patient and caregiver experiences will be shared in this presentation.

**Discussion or Conclusions**

This presentation highlights how the IAB is informing the ongoing conduct of a PA intervention and trial that has the potential for wide-spread implementation efforts in pediatric cancer care for resources that may enhance participant wellness, build hope and support quality of life.

**132**

### 8.7. Exploring Factors Associated with the Uptake of Exercise Recommendations among Hematological Cancer Patients in Canada

Joshua Tulk and Tavis Campbell.
Department of Psychology, Faculty of Arts, University of Calgary, Calgary, AB, Canada.

**Background/rationale or objectives/purpose**

Interventions promoting exercise can ameliorate treatment-related symptoms among individuals with hematological malignancies. However, poor uptake and adherence to recommendations are barriers. Less than half of the individuals with cancer will receive exercise recommendations from healthcare providers and up to 55% of individuals enrolled in exercise programs will drop out.

Improving the uptake and adherence to evidence-based recommendations depends on understanding patient and provider experiences with exercise recommendations, including knowledge, acceptance of recommendations, barriers and preferences for receiving interventions. Gaining knowledge about these concerns could provide the requisite knowledge to develop targeted interventions that improve the implementation of and adherence to exercise guidelines in cancer care.

**Methodology or Methods**

Individuals diagnosed with any stage or type of hematological cancer who have completed treatment and psychosocial healthcare providers will be recruited from across Canada. Following the ORBIT model, participants will complete a series of validated outcome measures and researcher-generated questions. A purposive subsample will also complete qualitative interviews. Topics covered will include the history of symptoms, current functioning, if they received recommendations, perceived barriers/facilitators to engage in and preferences for receiving exercise programs. Recurring themes and prevalence estimates will be identified from the data through descriptive and thematic analyses and translated into requirements and recommendations to adapt behavioral interventions.

**Impact on practice or results**

The results will provide insight into the factors associated with the implementation and uptake of evidence-based exercise interventions in Canada.

**Discussion or Conclusions**

In line with the ORBIT model, future research will use recommendations from this study to guide exercise program development and dissemination.

## 9. Final Category: H. Equity, Diversity and Inclusion in Cancer Care and Research

**19**

### 9.1. Diverse Community Members’ Views on Awareness of and Access to Psychosocial Oncology Resources and Services in Nova Scotia

Janice Howes, Marianne Arab and Leslie Hill.
NS Health, Halifax, Canada.

**Background/rationale or objectives/purpose**

Connecting cancer patients with helpful Psychosocial Oncology (PSO) resources in the Cancer Clinics in Nova Scotia can be challenging, as evidenced by the recent survey results of patients and healthcare providers. To better understand African Nova Scotians’ and 2SLGBTQIA+ community members’ views on the awareness of and access to PSO resources, two focus groups were held.

**Methodology or Methods**

A focus group with members of the African Nova Scotian community affected by cancer was held in March 2023 (N = 11) and a focus group with members of the 2SLGBTQIA+ community was held in May 2023 (N = 6). We were interested in what PSO services participants had accessed, the value of these services, barriers to access, how to improve access and best time to access PSO services.

**Impact on practice or results**

The qualitative results revealed that participants from the African Nova Scotian and 2SLGBTQIA+ communities were generally unaware of PSO resources. Despite this, they noted that these services are valuable. Barriers to access that were identified included being unaware of the services, stigma of cancer and needing help, discrimination and services moving to online access. Suggestions for improving access included support (peer and community) and specific clinical/educational programs offered in the community (outreach).

**Discussion or Conclusions**

It is important to address systemic bias, unconscious bias and our individual biases so that patients from all communities feel safe and supported during their cancer experience. We are exploring steps to help make members of diverse communities more aware of PSO resources (e.g., community outreach) and increasing resources targeted specifically at diverse communities.

**37**

### 9.2. “Sharing Our Stories Isn’t Benign”: Preferred Practices and Areas for Improvement While Working with Patient Advisors in Committees

Sevtap Savas ^1,2^, Nadine Frisk ^2,3^, Tristan Bilash ^2,4^, Chantale Thurston ^2,5^ and Kimberley Thibodeau ^2,6^.

^1^  Memorial University of Newfoundland and Labrador, St. John’s, Canada.
^2^  Canadian Association of Psychosocial Oncology (CAPO)—Advocacy Committee, Toronto, Canada.
^3^  Patient advisor, British Columbia, Canada.
^4^  Patient advisor, Saskatchewan, Canada.
^5^  Patient advisor, Manitoba, Canada.
^6^  McGill University Health Center, Montreal, Canada.

**Background/rationale or objectives/purpose**

Patient advisors are essential to improve clinical practice, research, knowledge dissemination and healthcare policies in psychosocial oncology (PSO). Understanding what works and what does not work is crucial for meaningful and effective partnerships with patient advisors.

Our goal is to reflect on our experience with serving in the committees (including the Canadian Association of Psychosocial Oncology—Advocacy Committee) and to make recommendations about the preferred practices while working with patient advisors.

**Methodology or Methods**

Perspectives are presented from the lenses of both patient advisor and non-patient advisor members. Self-reflections and transcript of a CAPO webinar we delivered in 2023 were used to formulate key perspectives and preferred practices.

**Impact on practice or results**

Patient advisors are enthusiastic to do things better for the next patient. While doing this, they also put themselves in vulnerable positions. Emotional, employment-related, technical, accessibility-related and financial barriers to participation exist. To have representation by diverse groups, the removal of these barriers, as well as building guidance, awareness and a desire for patient advisors are critical. Having a safe and welcoming space to contribute to the committee activities, compensation and having continuous and authentic connections that also appreciate and value advisor contributions were among the preferred practices.

**Discussion or Conclusions**

PSO needs to integrate patient and family voices to be effective. To do so, providing optimum conditions is essential by removing barriers and addressing needs and priorities expressed by patient advisors. Building guidance and the implementation of preferred practices through continuous, authentic and clear communications are encouraged for all committees working with patient advisors.

**41**

### 9.3. Exploring Barriers and Facilitators to the Inclusion of Diverse Populations into Patient and Family Advisory Councils in Cancer Care and Research: A Scoping Review

Madison Leia ^1^, Kaitlin See ^1^, Christie Farrer Rogers ^1^, Don Wood and Colleen Cuthbert ^1^.

^1^  University of Calgary, Calgary, Canada.

**Background/rationale or objectives/purpose**

Ensuring diverse patient engagement is critical for addressing disparities in health outcomes and healthcare access. Patient and family advisory councils (PFACs) are a key mechanism for patient engagement in cancer research, policy and healthcare delivery. As part of a broader review to understand the engagement of under-represented populations in PFACs, this study aims to (1) understand which groups have been included when trying to increase diversity in PFACs and (2) synthesize barriers and facilitators supporting the recruitment, retention and engagement of diverse populations into cancer-focused PFACs.

**Methodology or Methods**

The review was conducted following Levac, Colquhoun and O’Brien’s updated scoping review methodology. Electronic databases MEDLINE, EMBASE, PsycINFO, CINAHL and Scopus were systemically searched for peer-reviewed articles published before 19 July 2023. Two authors screened titles, abstracts and full texts independently to determine inclusion and extracted data from eligible full-text publications for quantitative and qualitative analysis.

**Impact on practice or results**

Only six papers (out of n = 44) were found on cancer-focused PFACs, all from Western countries. Four targeted specific racial/ethnic groups, one targeted ethnic minorities broadly and one focused on non-specific equity categories including age, location, disability, gender and sexuality. The findings suggest that barriers and facilitators align closely with established healthcare accessibility frameworks (“approachable, acceptable, available, affordable, and appropriate”).

**Discussion or Conclusions**

Despite increasing attention to equity, diversity and inclusion in research and health services, there is limited literature on engaging diverse cancer patients, families and the public in PFACs. Lessons from other health contexts could inform cancer care and research to bolster the inclusion of under-represented groups.

**76**

### 9.4. Reimagining Advanced Care Planning in Cancer Care through an Accessibility Lens

Karine Diedrich and Merry Parkinson.
Canadian Hospice Palliative Care Association (CHPCA), Ottawa, Canada.

**Background/rationale or objectives/purpose**

As Canada’s population ages, addressing accessibility needs becomes crucial, especially in healthcare for seniors who face higher disability rates and increased cancer diagnoses. Advanced Care Planning (ACP) offers a solution, facilitating clear treatment preferences, reducing unwanted interventions and improving quality of life. Despite its importance, many Canadians still do not engage in ACP.

**Methodology or Methods**

To tackle this issue, Advanced Care Planning Canada collaborated with Healthcare Human Factors (HHF) to develop a more accessible ACP workbook. Grounded in universal design principles and informed by extensive consultations with stakeholders, including people with disabilities, caregivers and healthcare providers, the workbook underwent significant revisions.

**Impact on practice or results**

This initiative enhances ACP engagement and understanding by providing practitioners with a broader understanding of ACP’s value and a more accessible tool to support patients. It empowers healthcare professionals to deliver patient-centered care and actively involve patients and their families in ACP discussions.

**Discussion or Conclusions**

Moving forward, ACP Canada aims to further improve ACP engagement across various healthcare settings, emphasizing disability awareness and inclusivity. By incorporating lessons learned from this initiative, the initiative seeks to continually enhance accessibility and promote person-centered care nationwide.

**91**

### 9.5. Punjabi Exercise Oncology YouTube Series: Tracking Reach and Satisfaction

Mannat Bansal, Imrose Bhullar, Meghan H. McDonough, Tanvir C. Turin and S. Nicole Culos-Reed.
University of Calgary, Calgary, Canada.

**Background/rationale or objectives/purpose**

Individuals of South Asian heritage, the largest ethnic minority group in Canada, are disproportionately impacted by certain cancers. While physical activity (PA) improves health, South Asian individuals living with cancer encounter barriers to PA participation. This includes language barriers to exercise oncology resources. There is a need to develop and disseminate resources in South Asian ethnic languages to facilitate access and increase PA participation.

**Methodology or Methods**

Twelve weeks of Punjabi (the most common South Asian ethnic language) exercise videos based on the Exercise for Cancer to Enhance Living well (EXCEL) classes are being filmed and will be posted on YouTube. Outreach will utilize social media and engage existing network of healthcare providers, exercise professionals and Punjabi participants. Each video will conclude with a survey link for feedback to assess satisfaction and value of the video, along with ways to further disseminate the videos and reach more individuals within this population.

**Impact on practice or results**

Dissemination methods will be tracked and reach will be assessed by tracking online views. The value and satisfaction will be described qualitatively from the open-ended survey responses. The results of this study can inform the ongoing translation and dissemination of Punjabi exercise oncology resources.

**Discussion or Conclusions**

This work is critical to building a more diverse and inclusive exercise oncology environment as part of supportive cancer care and will ensure resources will be available to enhance South Asian engagement. Individuals of South Asian heritage living with and beyond cancer may benefit from the potential health outcomes of exercise oncology resources, ultimately enhancing their quality of life.

**104**

### 9.6. Enhancing Accessibility through Equitable Implementation of Digital Health Interventions: A Concept Analysis of Digital Health Equity

Sydney Wasserman ^1^ and Sylvie Lambert ^1,2^.

^1^  McGill University, Montreal, Canada.
^2^  St. Mary’s Research Center, Montreal, Canada.

**Background/rationale or objectives/purpose**

Self-management interventions are a cost-effective and accessible way for patients living with cancer to manage their cancer-related symptoms. As these interventions become increasingly digitized, it is imperative to understand digital health equity for the more accessible development and dissemination of interventions. The objective of this paper is to analyze the concept of digital health equity to better understand its implications for theory, research and intervention implementation.

**Methodology or Methods**

This paper follows Rodgers’ evolutionary method for concept analysis, exploring findings from a literature review to identify attributes (characteristics used to define a concept), antecedents (incidents before the development of a concept) and consequences (results of using the concept in practice) of “digital health equity”.

**Impact on practice or results**

The search yielded a total of 62 articles, 19 of which were included for analysis. The *attributes* were **1.** determining the needs and usage patterns of end-users based on social determinants of health, **2.** designing digital health interventions that promote accessibility (e.g., gamification features), **3.** shifting healthcare culture towards more acceptance of technology and **4.** identifying users’ technical proficiency. The *antecedents* included **1.** having physical and psychosocial barriers to care, **2.** having inequitable access to education and **3.** experiencing socio-economic and age health disparities. The *consequences* identified were **1.** a reduced burden on the healthcare system, **2.** improved quality of life and **3.** improved health behavior outcomes.

**Discussion or Conclusions**

Understanding digital health equity is fundamental for enhancing the accessibility of care among those living with cancer. These findings may support researchers in adopting equitable strategies when developing digital self-management interventions.

**134**

### 9.7. “They Make Me Feel Whole”—The Significance of Identity for Racially, Ethnically, Gender and Sexually Diverse Adolescents and Young Adults with Cancer

Kaitlyn Hanson ^1^, Andrew Hatala ^2^, Mirha Zohair ^1^, Ian Scott ^3^, Morgan Stirling ^2^, Kristin Wilson ^4^, Roxanne van Velzen ^5^, Allan Garland ^2^, Alyson Mahar ^6^ and Sapna Oberoi ^7,8^.

^1^  Max Rady College of Medicine, University of Manitoba, Winnipeg, Canada.
^2^  Department of Community Health Sciences, Max Rady College of Medicine, University of Manitoba, Winnipeg, Canada.
^3^  CancerCare Manitoba, Winnipeg, Canada.
^4^  Patient Partner, Canada.
^5^  Patient Partner, Winnipeg, Canada.
^6^  Queen’s University, Kingston, Canada.
^7^  Department of Pediatrics and Child Health, Max Rady College of Medicine, University of Manitoba, Winnipeg, Canada.
^8^  Department of Pediatric Hematology–Oncology, CancerCare Manitoba, Winnipeg, Canada.

**Background/rationale or objectives/purpose**

Adolescents and young adults (AYAs, 18–39 years old) with cancer identifying as racially/ethnically diverse or 2SLGBTQIA+ have been under-represented in cancer research. We explored aspects of identity that matter most to these diverse AYAs and how they affect their experiences in the healthcare system.

**Methodology or Methods**

Eligible participants were English-speaking, racially/ethnically diverse and/or 2SLGBTQIA+ AYAs who were diagnosed with cancer between the ages of 15 to 39, currently aged ≥ 18 and receiving or have received cancer care in the Canadian healthcare system. Five patient partners who met the same inclusion criteria were also recruited as research collaborators. Participants were interviewed using a semi-structured interview guide. Framework analysis was used for qualitative analysis.

**Impact on practice or results**

We recruited 23 participants from four provinces (mean age: 28, Range: 20–44); 17 participants identified as racially/ethnically diverse, 1 as sexual/gender diverse and 5 as racially/ethnically and sexually/gender diverse. The identities most significant to participants included their culture/ethnicity, religion/spirituality and sexuality. Only one participant emphasized that being a person with cancer was an essential aspect of their identity. Cancer diagnosis altered the aspects of identity that participants deemed most important. Both visible and invisible aspects of identity shaped participants’ experiences and influenced their trust in the healthcare system.

**Discussion or Conclusions**

Racially, ethnically, gender or sexually diverse AYAs with cancer place significant value on aspects of their identity that align with the communities they identify with. Understanding and acknowledging these identities are crucial for healthcare professionals to provide safe and effective care.

**135**

### 9.8. Understanding the Experiences of Racially, Ethnically, Gender and Sexually Diverse Adolescents and Young Adults with Cancer: A Qualitative Analysis

Kaitlyn Hanson ^1^, Andrew Hatala ^2^, Mirha Zohair ^1^, Ian Scott ^3^, Fiona Schulte ^4^, Vinesha Ramasamy ^5^, Zeba Tayabee ^5^, Allan Garland ^2^, Alyson Mahar ^6^ and Sapna Oberoi ^7,8^.

^1^  Max Rady College of Medicine, University of Manitoba, Winnipeg, Canada.
^2^  Department of Community Health Sciences, Max Rady College of Medicine, University of Manitoba, Winnipeg, Canada.
^3^  CancerCare Manitoba, Winnipeg, Canada.
^4^  Department of Oncology, Division of Psychosocial Oncology, Cumming School of Medicine, University of Calgary, Calgary, Canada.
^5^  Patient Partner, Canada.
^6^  Queens University, Kingston, Canada.
^7^  Department of Pediatrics and Child Health, Max Rady College of Medicine, University of Manitoba, Winnipeg, Canada.
^8^  Department of Pediatric Hematology–Oncology, CancerCare Manitoba, Winnipeg, Canada.

**Background/rationale or objectives/purpose**

This study aimed to investigate the experiences of adolescents and young adults (AYAs) with cancer from racially/ethnically diverse and/or 2SLGBTQIA+ communities within the Canadian healthcare system to identify areas for improvement in their cancer care experience.

**Methodology or Methods**

This study included participants who identified as racially/ethnically diverse and/or 2SLGBTQIA+, diagnosed with cancer (15–39 years), currently ≥18 years and received or were receiving cancer care in Canada. Patient partners with lived experience of cancer were recruited as collaborators. Semi-structured virtual interviews were conducted using an interview guide and transcripts were analyzed using framework analysis.

**Impact on practice or results**

Four of the five recruited patient partners were involved as collaborators, while 23 participants (17 racially/ethnically diverse; 1 sexually/gender diverse and 5 racially and sexually diverse) were interviewed (mean age: 28, range: 20–44). The participants reported both positive and negative experiences with the healthcare system. The most common themes of positive experiences included the ability to identify with healthcare providers, effective communication and information sharing. The prominent themes of unfavorable experiences included judgmental attitudes and racialization by healthcare providers, needing to advocate for resources and barriers to care. The participants’ recommendations included increasing the proportion of diverse healthcare professions, implementing equity, diversity and inclusivity (EDI) training and improving communication for improving cancer care experiences.

**Discussion or Conclusions**

Experiences of AYAs with cancer in Canada are influenced by their diverse racial, ethnic and LGBTQIA+ identities. To provide better care, cancer organizations should integrate principles of EDI at all levels, ensuring safe and inclusive environments for all patients.

## 10. Final Category: J. Implementation Science, Knowledge Translation and Synthesis

**21**

### 10.1. Optimization of Codeine Prescription and Dosing in Clinical Care Nursing: Recommendation for Genetic Testing and Precision Medicine Implementation

Florence Emenji Asaba ^1^ and Joy Johnson Agbo ^2^.

^1^  Cyprus International University, Leftkosia, Cyprus.
^2^  Cyprus International University, North Cyprus, Leftkosia, Cyprus.

**Background/rationale or objectives/purpose**

Codeine is an opioid analgesic that is used to treat pain, cough, cold and diarrhea. It is metabolized by an enzyme known as CYP2D6 to the more potent morphine. It is generally well-tolerated. The primary limitation is related to severe and sometimes life threatening and fatal toxicity/adverse drug reaction (ADR). On the other hand, some patients do experience sub-optimal or no analgesic effect (therapeutic failure).

**Methodology or Methods**

Clinical practice recommendations were developed on “CYP2D6 and its relevance to codeine use” following a comprehensive and systematic review of published studies, as well as clinical practice guidelines from authoritative academic and professional societies, institutions and regulatory bodies.

**Impact on practice or results**

Based on the available evidence and content expert clinical opinion, CPIC, HCSC, FDA, DPWG, CPNDS, Swissmedic and PMDA recommend *CYP2D6* genetic testing in patients prior to the administration of codeine.

**Discussion or Conclusions**

ULTRARAPID METABOLIZER (MORPHINE INTOXICATION): avoid prescribing codeine due to potential risk for toxicity/ADR and consider alternative analgesics that are not metabolized by the CYP2D6 enzyme.

NORMAL METABOLIZER (NORMAL RESPONSE): a standard recommended dose of codeine may be used.

INTERMEDIATE METABOLIZER (INTERMEDIATE RESPONSE): A standard recommended dose of codeine may be used, but the patient should be monitored for reduced efficacy. In case of an inadequate response, consider increasing the codeine dose. If the response is still inadequate, then consider alternative analgesics not metabolized by the CYP2D6 enzyme.

POOR METABOLIZER (POOR RESPONSE): should avoid codeine use due to potential risk for poor or no response and consider alternative analgesics not metabolized by the CYP2D6 enzyme.

**77**

### 10.2. Introducing the Ontario Health (Cancer Care Ontario) Fear of Cancer Recurrence Guideline

Sophie Lebel ^1^, Caroline Zwaal ^2^, Lise Craig, Randy Conrod, Laurie Freeman ^3^, Jacqueline Galica ^4^, Christine Maheu ^5^, Rinat Nissim ^6^ and Josée Savard ^7^.

^1^  University of Ottawa, Ottawa, Canada.
^2^  McMaster University, Hamilton, Canada.
^3^  University of Windsor, Windsor, Canada.
^4^  Queens University, Kingston, Canada.
^5^  McGill University, Montreal, Canada.
^6^  Princess Margaret Hospital, Toronto, Canada.
^7^  Université Laval, Québec, Canada.

**Background/rationale or objectives/purpose**

The guidelines make recommendations based on evidence-based strategies and/or interventions to screen, assess and manage fear of cancer recurrence (FCR) in adults living with cancer and their care partners to improve patient outcomes.

**Methodology or Methods**

The Fear of Cancer Recurrence Guideline Working Group, composed of clinical psychologists, nurses, researchers and patient representatives, was convened at the request of Ontario Health Psychosocial Oncology Program. The Group worked from November 2021 to January 2024 with the Program in Evidence-Based Care to produce evidence-based and evidence-informed guidance documents. The process included a systematic review, interpretation of the evidence by the Working Group and draft recommendations, internal review by content and methodology experts and external review by Ontario clinicians and other stakeholders.

**Impact on practice or results**

Based on the reviewed evidence, we recommended that adults living with cancer complete a single-item screener, the FCR-1r, at each clinical encounter because it can be added into the ESAS, which is currently administered across cancer centers in Ontario. We recommend that those identified as scoring five and more on the FCR-1r complete a standardized FCR measure. We propose a matched care approach for interventions based on the level of FCR (low, moderate and high), starting with psychoeducation for all and reserving FCR-specific CBT of mind–body interventions for those with high FCR. More research is needed to strengthen recommendations for care partners.

**Discussion or Conclusions**

The guidelines focus on recommendations that can be implemented in cancer centers with relative ease and that make use of existing resources.

**83**

### 10.3. Implementation of an Electronic Prospective Surveillance System for Cancer Rehabilitation: Preliminary Results of a Mixed Methods Study

Christian Lopez ^1,2^, Sarah Neil-Sztramko ^3^, Kristin Campbell ^4^, Jackie Bender ^1^, Gillian Strudwick ^5^, David Langelier ^6^, Tony Reiman ^7^, Jonathan Greenland ^8^ and Jennifer Jones ^1,9^.

^1^  Department of Supportive Care, Princess Margaret Cancer Center, Toronto, Canada.
^2^  Institute of Medical Science, University of Toronto, Toronto, Canada.
^3^  Faculty of Health Sciences, McMaster University, Hamilton, Canada.
^4^  Department of Physical Therapy, University of British Columbia, Vancouver, Canada.
^5^  Institute of Health Policy, Management and Evaluation, University of Toronto, Toronto, Canada.
^6^  Cumming School of Medicine, Department of Clinical Neurosciences, University of Calgary, Calgary, Canada.
^7^  Department of Oncology, Saint John Regional Hospital, Saint John, Canada.
^8^  Dr. H. Bliss Murphy Cancer Center, Eastern Health, St. John’s, Canada.
^9^  Institute of Medical Science, University of Toronto.

**Background/rationale or objectives/purpose**

REACH is an electronic prospective surveillance system implemented at four Canadian centers for four disease sites (breast, colorectal, head and neck and lymphoma). REACH remotely monitors cancer survivors’ rehabilitation needs via patient-reported outcomes during and after cancer treatment and delivers support, including links to self-management education and community programs and recommendations for further clinical screening. We report on the preliminary results for the implementation at the first and largest center (Princess Margaret Cancer Centre).

**Methodology or Methods**

A 4-cycle mixed-methods formative evaluation guided by the Implementation Outcomes Framework was conducted to adapt the implementation to address barriers identified during each cycle. Data collection is ongoing. System usage data and documentation of meetings with clinic leadership were analyzed up to 10-months post-implementation. Qualitative data were categorized using the Consolidated Framework for Implementation Research.

**Impact on practice or results**

To date, n = 301 patients have registered to REACH and n = 250 (41% breast, mean age 58 ± 13, 43% currently receiving treatment) have provided consent for research. Preliminary analyses indicate REACH is feasible (the mean duration to complete assessments is 5 min) and has high fidelity (88% assessments completed, 69% of patients viewing ≥ 1 resource recommended). Compatibility with the clinic workflows and systems, relative priorities among clinic staff and meaningful clinic leadership engagement have been identified as critical for successful implementation.

**Discussion or Conclusions**

The preliminary results suggest that a tailored implementation plan can support the implementation of electronic prospective surveillance systems into routine care. A description of the implementation plan along with updated data (a post-implementation patient evaluation survey and interviews with clinic leadership) will be presented.

**106**

### 10.4. Environmental Scan of Programming and Services for Adolescent and Young Adult Cancer Patients across Canada

Nicole Rutkowski ^1,2^, Perri Tutelman ^2,3^, Sara Beattie ^2,3^, Chantale Thurston ^4^ and Fiona Schulte ^3^.

^1^  University of Ottawa, Faculty of Social Sciences, School of Psychology, Ottawa, Canada.
^2^  Psychosocial Oncology, Tom Baker Cancer Center, Calgary, Canada.
^3^  University of Calgary, Department of Oncology, Calgary, Canada.
^4^  Patient Partner, AYA CAN, Winnipeg, Canada.

**Background/rationale or objectives/purpose**

Adolescents and Young Adults (AYAs), ages 15–39 years, are at a developmentally complex period of life and face unique psychosocial challenges when diagnosed with cancer. The incidence of AYA cancers has steadily increased in Canada over the past two decades and the growing number of AYA survivors face a lifetime of physical, cognitive and psychosocial challenges that require specialized support. The purpose of this study is to conduct an environmental scan of current AYA-specific programming and services being offered across Canada.

**Methodology or Methods**

A survey assessing AYA services will be sent out to all pediatric and tertiary hospitals across Canada. The survey will gather data on program logistics, AYA specialized staff and training opportunities, funding, and specific areas of interest for AYA care such as palliative care, fertility, fatigue, and sexual health. Preliminary results will be presented.

**Impact on practice or results**

The environmental scan will provide a comprehensive picture of current services being offered to address AYA-specific needs, as well as identify gaps and barriers to care. The information gathered will facilitate prioritizing program development, implementation and research to address gaps in AYA care, as well as facilitate the identification of existing programming from other provinces that may be implemented or adapted.

**Discussion or Conclusions**

AYAs have distinctive and complex needs that require specialized care and support given their stage of life and the disruptive nature of cancer on the individual and their family. An environmental scan provides a map to existing programming that may be occurring in isolation across Canada.

**109**

### 10.5. Grief Literacy in Canada: Contributing to National Action Plan for Grief

Marney Thompson ^1,2^ and Shelly Cory ^2,3^.

^1^  Victoria Hospice Bereavement Services, Victoria, Canada.
^2^  Canadian Grief Alliance, Canada.
^3^  Canadian Virtual Hospice, Winnipeg, Canada.

**Background/rationale or objectives/purpose**

While grief is a normal reaction upon the diagnosis and progression of cancer and the life changes that follow, it is often not recognized or responded to constructively by patients, families and health care providers. With funding from the Government of Canada, the Canadian Grief Alliance (CGA) is leading a two-year initiative to advance grief literacy, build people’s capacity to understand and respond to grief and develop a National Action Plan for Grief.

**Methodology or Methods**

This workshop will provide an overview of grief literacy principles, share the CGA’s engagement process, carry out a deep dive into the results of the national grief survey of 3800 respondents and the input from cross-Canada consultations and, through small-group discussions, explore gaps and help define priorities for a National Action Plan for Grief.

Learning objectives:

1. Enhance skills to recognize, understand and respond constructively to grief.
2. Provide feedback on emerging themes from CGA consultations and the national survey and identify and explore gaps.
3. Determine priorities to inform a National Action Plan for Grief.

**Impact on practice or results**

This workshop will help participants recognize and respond constructively to patient and family grief, as well as their own grief and that of their colleagues. The participants will become more aware of emerging grief resources.

**Discussion or Conclusions**

This experiential workshop will provide participants with the opportunity to inform the direction of a National Action Plan for Grief and improve their grief literacy skills based on lessons learned from leaders in the field.

**119**

### 10.6. Co-Development of an Implementation Strategy to Establish a Provincial Adolescent and Young Adult (AYA) Cancer Program

Jonathan Avery ^1,2^, Alannah Smrke ^3^, Tiffany Hill ^1^, Ada Okonkwo ^1^ and Cheryl Heykoop ^1^.

^1^  Royal Roads University, Victoria, Canada.
^2^  Princess Margaret Cancer Center, Toronto, Canada.
^3^  BC Cancer, Vancouver, Canada.

**Background/rationale or objectives/purpose**

AYAs (individuals between 15 and 39 years of age) with cancer require life-stage-appropriate care. In Canada, there are few AYA-targeted strategies and in British Columbia (BC), there are currently no provincial AYA cancer care directives. This project seeks to co-design an implementation strategy to develop a provincial AYA cancer care strategy and supportive care program in BC.

**Methodology or Methods**

A qualitative descriptive design informed by appreciative inquiry was used to conduct one-on-one and dyadic semi-structured interviews with cancer care providers, program administrators and directors across the six provincial cancer care sites and BC Children’s hospital. Interviews explored strategies that could be utilized to integrate AYA cancer care in the provincial cancer care system. The constant comparative method was used to analyze the interviews.

**Impact on practice or results**

We interviewed 17 individuals including 8 oncologists, 6 supportive care providers, 2 program administrators/coordinators and 1 nurse practitioner. The results demonstrate three strategies to integrate AYA care within the provincial system: (1) recognition that AYAs are an equity-denied and vulnerable population, (2) integrate AYA care within current strategic initiatives and (3) co-develop clinical programs with AYAs with a focus on scalability.

**Discussion or Conclusions**

Our findings outline an implementation strategy to enhance care for AYAs. Through the utilization of this strategy, we have helped initiate a small pilot project to improve the connection of AYAs to patient and family counselling services at BC Cancer. Additionally, we secured funding from the Canadian Partnership Against Cancer to improve oncofertility support for AYAs residing in BC and the Yukon.

**120**

### 10.7. Where Do We Begin?: A Thematic Analysis Addressing Patient, Provider and Community Partner Concerns Regarding the Perinatal Period after Breast Cancer

Ruth Vanstone ^1^, Karen Fergus ^1,2^, Dalia Peres ^2^, Ellen Warner ^2^, Noor Niyar Ladhani ^2^ and Karen Glass ^2^.

^1^  York University, Toronto, Canada.
^2^  Sunnybrook Hospital, Toronto, Canada.

**Background/rationale or objectives/purpose**

A history of breast cancer introduces challenges for women who wish to conceive after treatment and there remains a dearth of accessible and empirically supported information to address concerns such as the safety of interrupting hormone therapy prior to conceiving and the impact of pregnancy on the risk of cancer recurrence. Further, the research shows that these information gaps are a significant source of distress for this population.

**Methodology or Methods**

The current analysis aims to address information gaps for women in the perinatal period who have a history of breast cancer and their healthcare providers. Preliminary results of focus group discussions conducted with participant groups (patient, provider and community partners) were transcribed and analyzed using thematic analysis. The analysis revealed several areas of concern, including implications of ceasing hormone therapy to become pregnant, concerns around breastfeeding and healthcare providers’ uncertainty in relation to counselling women during this time.

**Impact on practice or results**

These data may guide the development of educational resources for the key participant groups. In conjunction with a scoping review of the current literature, we can provide patients, providers and community partners with up-to-date empirical information that addresses their concerns, with the intention of minimizing patient distress and increasing communication.

**Discussion or Conclusions**

This research provides the foundation for a knowledge translation tool, which will be accessible to patients, healthcare providers and community partners committed to supporting women of childbearing age with a history of breast cancer. This tool will allow patients to actively engage in their care, foster collaborative decision making and lead to improved psychosocial outcomes.

**130**

### 10.8. An Update on the Implementation of the Fear of Recurrence Therapy (FORT) Group Intervention in Five Cancer Centers across Canada

Alanna Chu ^1^, Sophie Lebel ^1^, Sara Beattie ^2^, Sheila Garland ^3^, Cheryl Harris ^4^, Jennifer Jones ^5^, Christine Maheu ^6^, Jackie Bender ^5^, Andrea Feldstain ^2^ and Linda Carlson ^7^.

^1^  University of Ottawa, Ottawa, Canada.
^2^  Tom Baker Cancer Center, Calgary, Canada.
^3^  Memorial University, St. John, Canada.
^4^  The Ottawa Hospital, Ottawa, Canada.
^5^  Princess Margaret Cancer Center, Toronto, Canada.
^6^  McGill University, Montreal, Canada.
^7^  University of Calgary Cumming School of Medicine, Calgary, Canada.

**Background/rationale or objectives/purpose**

Fear of cancer recurrence (FCR) has been identified as the number one unmet need of cancer survivors, with 59% of cancer survivors reporting clinical levels of FCR. The Fear of Recurrence Therapy (FORT) intervention is a six-week, cognitive-existential group therapy that has been shown to effectively reduce FCR in cancer survivors in a randomized controlled trial. However, there is a well-documented knowledge to clinical practice gap, owing to difficulties with implementation. With support from the Canadian Cancer Society, this study aims to assess the implementation of FORT in five Canadian cancer centers.

**Methodology or Methods**

We are conducting a mixed-methods comparative case study, guided by the RE-AIM framework. Phase I: Qualitative interviews (n = 19) were conducted with clinicians, staff and decision makers at all five centers to assess site-specific barriers and create site-tailored implementation strategies. Phase II (ongoing): implementing 2 FORT groups at each site for female-identifying people with breast, gynecological or hematological cancer and collecting implementation outcome data (e.g., number of therapists involved in the delivery of FORT, effectiveness, fidelity and costs).

**Impact on practice or results**

Phase I: each site reported varying implementation considerations (e.g., population spread, structure of cancer unit, psychosocial expertise and competing priorities) and strategies (e.g., hybrid training, combining resources, identifying champions). Phase II: training has been completed in some sites and participant recruitment will begin in April 2024.

**Discussion or Conclusions**

This presentation will report on the implementation of FORT, an evidence-based intervention for FCR in clinical settings. Our findings will help inform navigation of facilitators and barriers when implementing evidence-based interventions in real world clinical settings.

**136**

### 10.9. Preliminary Psychometric Properties of the Child Loneliness Scale for Survivors of Pediatric Brain Tumor

Tessa Chomistek, Hailey Zwicker, Caitlin Forbes, Courtney Tromburg, Mehak Stokoe and Fiona Schulte.
University of Calgary, Calgary, Canada.

**Background/rationale or Objectives/purpose**

Survivors of pediatric brain tumors (SPBT) often face social difficulties following their cancer treatment. There is a current lack of tools to measure loneliness within a SPBT sample. Our aim was to evaluate the psychometric properties of the Child Loneliness Scale (CLS) in a SPBT sample.

**Methodology or Methods**

SPBT (*n* = 33, 54.5% female, mean [SD] age = 13.5 [3.22] years, mean time since diagnosis 7.53 [3.20] years) were recruited from the Alberta Children’s Hospital. Participants completed the CLS. Each item was rated on a Likert scale from one (Always True) to five (Not True at All). Filler items were removed from the original 24 items leaving a total of 16 items. Psychometric properties including skewness, kurtosis and floor/ceiling effects of the CLS and individual items were evaluated.

**Impact on practice or results**

Individual items had acceptable psychometric characteristics, apart from item 24 which performed poorly across the analyses. Some items were slightly positively skewed between 0.66 and 1.60 and the remainder were slightly negatively skewed between −0.08 and −1.998, apart from one item, which was more significantly skewed at −2.72. The majority of the items were platykurtic while the remaining items were leptokurtic. The participants’ CLS total scores ranged from 38 to 61, which falls in the middle of the possible total range of 16 to 80. No floor or ceiling effects were found.

**Discussion or Conclusions**

The preliminary analysis suggests that the CLS may be used effectively with SPBT although further investigation is needed and should include a larger sample size.

## 11. Final Category: K. Innovation in Psychosocial Oncology Interventions

**11**

### 11.1. Beyond Paper-and-Pencil: Electronic Tools for Quality of Life Assessment in Pediatric Advanced Cancer

Lye-Ann Robichaud ^1,2,3^, Michel Duval ^4,5^, Bruno Michon ^6,7^, Marianne Olivier-D’Avignon ^8^, Mathias Tyo-Gomez ^9^, Marc-Antoine Marquis ^4,10^ and Serge Sultan ^1,3,5^.

^1^  Département de Psychologie, Université de Montréal, Montréal, Canada.
^2^  Centre de Recherche, Centre hospitalier universitaire (CHU) Sainte-Justine, Montréal, Canada.
^3^  Centre de Psycho-Oncologie (CPO), CHU Sainte-Justine, Montréal, Canada.
^4^  Département de Pédiatrie, Université de Montréal, Montréal, Canada.
^5^  Département D’hémato-Oncologie, CHU Sainte-Justine, Montréal, Canada.
^6^  Centre Mère-Enfant Soleil, CHU de Québec-Université Laval, Québec, Canada.
^7^  CHU de Québec-Université Laval, Québec, Canada.
^8^  École de Travail Social, Université Laval, Québec, Canada.
^9^  Patient Partenaire, CHU Sainte-Justine, Montréal, Canada.
^10^  Département de Pédiatrie Générale, CHU Sainte-Justine, Montréal, Canada.

**Background/rationale or objectives/purpose**

Assessing Quality of Life (QoL) in advanced cancer is a challenge. Advance QoL is a questionnaire developed specifically for young people with advanced cancer. Two self-reported paper-and-pencil versions (8–12 years; 13–18 years) have previously been developed. The present project aims at testing electronic versions of the tools to facilitate completion by young people.

**Methodology or Methods**

The goal is to recruit 20 French-speaking patients (10 per age group) at CHU Sainte-Justine, CHU de Québec Université-Laval and in collaboration with Leucan, a non-profit organization. Participants must have been diagnosed with cancer ≥ 3 months and be receiving cancer care. Data are collected via 45 min virtual cognitive Think-Aloud interviews. Participants are invited to complete *Advance QoL,* informing on their current status. Participants also complete a satisfaction questionnaire to assess the social validity of *Advance QoL* and a short sociodemographic questionnaire.

**Impact on practice or results**

The interviews are analyzed using a method for interpreting cognitive interviews for instrument development. The preliminary results suggest that the electronic tools are appreciated, easy to use and relevant. The satisfaction measures suggest that it is comparable with the paper–pencil version.

Minor issues, such as validation rules and information gaps in the radar chart, will be addressed through review and revision by the research team, including a resource patient and a team of young collaborators.

**Discussion or Conclusions**

An electronic questionnaire empowers youths by including their voices in QoL discussions and aligns with their preference. The REDCap version will also facilitate multisite data collection. The collected data would facilitate the implementation of targeted interventions to improve overall well-being.

**12**

### 11.2. Mind Matters: Decoding the Psychological Challenges of Breast Cancer with an Integrative Framework and Clinical Tool for Mental Health

Justine Fortin ^1,2,3^, Émilie Rudd ^3,4^, Claudia Trudel-Fitzgerald ^3,5,6^, Alain Brunet ^2,7^ and Marie-France Marin ^1,3^.

^1^  Department of Psychology, Université du Québec à Montréal, Quebec, Canada.
^2^  Douglas Institute Research Center, Quebec, Canada.
^3^  Research Center of the Institut Universitaire en Santé Mentale de Montréal, Quebec, Canada.
^4^  Department of Psychology, Université de Montréal, Quebec, Canada.
^5^  Department of Psychology, Université du Québec à Trois-Rivières, Quebec, Canada.
^6^  Lee Kum Sheung Center for Health and Happiness, Harvard T.H. Chan School of Public Health, Massachusetts, USA.
^7^  Department of Psychiatry, McGill University, Quebec, Canada.

**Background/rationale or objectives/purpose**

Existing integrative psycho-oncology models of mental illness and health are designed for all types of cancer, even though patients’ experiences and issues may vary depending on the specific cancer diagnosis. **Objectives/purpose**: we conducted a narrative review to summarize the different theories and models of mental illness and health related to the breast cancer experience and propose an integrative phasic model for the breast cancer trajectory.

**Methodology or Methods**

**Sample and setting**: five databases were searched for articles or unpublished studies related to breast cancer models and theories of mental health and illness: PsycInfo, Medline, Web of Science, PubMed and CINAHL. **Procedures**: the PRISMA methodology was followed to extract the essential information from the included articles.

**Impact on practice or Results**

**Results**: From the articles extracted, seven landmarks’ phases emerged: screening, diagnosis, post-diagnosis/pretreatment, treatment, post-treatment, survivorship and recurrence. The literature highlights each of these phases’ pathogenic challenge leading to six psychological patterns: (1) psychological distress with anxious features, (2) psychological distress with depressive features, (3) non-specific distress, (4) psychological distress with trauma and stressor-related features, (5) low health-related quality of life and (6) fear of recurrence. These findings informed the creation of a clinical tool for the real-time monitoring of patients’ symptoms and quality of life, designed for both patients and professionals.

**Discussion or Conclusions**

**Conclusion and clinical implications**: The clinical tool incorporates scientific findings and addresses patient experiences and challenges, guiding clinicians to tailor mental health treatments to patients needs, particularly non-resilience trajectories. Moreover, the tool may enhance patient empowerment by allowing them to actively participate in their healthcare decisions.

**49**

### 11.3. Health Coaching in Oncology Settings

A. Justine Dowd, S. Nicole Culos Reed and Julia T. Daun.
University of Calgary, Calgary, Canada.

**Background/rationale or objectives/purpose**

Health coaching is a rapidly growing field for the prevention and management of chronic diseases, including cancer. Health coaching is defined as using evidence-based conversational skills, motivational interviewing and clinical strategies to assist patients in achieving health behavior change goals. The goal of a health coach is to provide tailored support (education, fostering self-regulatory skills) to equip patients with the skills required to make lifelong health behavior changes.

**Methodology or Methods**

Using demonstrations by the presenters, vignettes and role play, attendees will be engaged in opportunities to practice health coaching techniques taught in this interactive workshop.

**Impact on practice or results**

We will teach attendees health coaching skills to promote hope and better support patients in their oncology-related behavior change.

**Discussion or Conclusions**

Attendees will be able to apply health coaching skills to support individuals across the cancer spectrum to achieve their wellness goals and promote feelings of hope on the cancer journey.

**Learning objectives:**

Attendees will have introductory knowledge to:

- Apply HC skills to help patients develop and successfully achieve goals through changing attitudes and behaviors.
- Support patients in health-promoting activities to develop maintenance in behavior change.
- Guide patients in effectively managing health conditions collaboratively with care providers.
- Support equity-deserving and Indigenous populations through culturally sensitive HC practices.
- Understand using HC skills within existing role(s).
- Promote self-compassion on the cancer journey.

**Teaching methods:**

- Demonstrations by the presenters;
- Vignettes to discuss in groups;
- Role play to apply learning.

**59**

### 11.4. Transitioning to a Virtual Group: Practical, Clinical and Evaluation Implications

Ceinwen Cumming ^1,2^, Salvatore B. Durante ^2^, Kim Crosby ^1,2^ and Treena Hinse ^1^.

^1^  Alberta Health Services, Edmonton, Canada.
^2^  University of Alberta, Edmonton, Canada.

**Background/rationale or objectives/purpose**

The purpose of this presentation is to describe the benefits and challenges of offering a virtual group in the clinical, psychosocial oncology setting. An innovative, single-session psycho-educational group was quickly moved to a virtual counseling platform in 2020. Before the COVID-19 pandemic, the group was successfully provided in-person for bereaved family members of cancer patients. Since the spring of 2020, the group has been available in a virtual format when there have been sufficient participants.

**Methodology or Methods**

The benefits and challenges of using the virtual format in the group are described and discussed in the context of the available literature in this presentation.

**Impact on practice or results**

It is documented in the literature that there are practical and clinical challenges and benefits that have emerged in psychological service provisions, generally from the impact of the COVID-19 pandemic. This presentation provides information for practitioners in the field of psychosocial oncology who are now frequently providing and evaluating both types of group formats in clinical service provision in rural, small town, urban and other environments.

**Discussion or Conclusions**

In summary, virtual and in-person formats have advantages and disadvantages in clinical service delivery. Future directions are outlined for clinical service, program evaluation and research in the context of the new focus on the provision of psychological services for cancer patients and family members in our area of the world.

**62**

### 11.5. Development of a Psychosocial Oncology Triage Service

Laura Labelle, Marie de Guzman Wilding, Sarah Hollingsworth and Guilherme Daga
Tom Baker Cancer Center, Supportive Care Services, Cancer Care Alberta, Calgary, Canada

**Background/rationale or objectives/purpose**

In response to the pandemic and with the implementation of Virtual Care, Psychosocial Oncology (PSO) counseling services have become more accessible. In 2021 alone, the Tom Baker Cancer Center (TBCC) saw a 24% annual increase in counseling referrals. Between 2017 and 2022, waiting times for counseling referrals at TBCC increased from 2–4 weeks to 6–12 weeks. In October 2021, the TBCC PSO introduced a psychosocial triage service to provide faster access to brief assessments, support and referrals.

**Methodology or Methods**

A new social work position was created to provide triage visits for “urgent” referrals and, when more information is needed, to direct a referral. The Triage Coordinator determines eligibility, offers resources and referrals and provides brief supportive counseling. Informal feedback from clinicians and service metrics were captured for a five-month period in 2022 to inform service development.

**Impact on practice or results**

From 1 May to 30 September 2022, 1441 patients were referred to Psychosocial Oncology, 365 (25%) received a triage visit and 41% of triaged patients subsequently received counseling within PSO. The results showed that the triage service may expedite urgent referrals and runs in parallel to other PSO services.

**Discussion or Conclusions**

The TBCC Psychosocial Triage service has evolved quickly since its implementation in early 2021. The service supports timely patient access and workforce optimization, enabling a ‘stepped’ model of psychosocial care. The data capture was limited by the previous electronic medical record (EMR). Paired with standardized report creation, evaluating patient experience is a top priority. As the service evolves, there will be a greater capacity to meet patients’ triage needs including earlier access for palliative care referrals.

**89**

### 11.6. A Feasibility Trial of Managing Cancer and Living Meaningfully (CALM) in Patients with Newly Diagnosed and Recurrent Advanced Ovarian Cancer

Megan George ^1,2,3^, Carmine Malfitano ^1^, Stephanie Lheureux ^4^, Lauren Philp ^4^ and Gary Rodin ^5^.

^1^  Princess Margaret Cancer Center, University Health Network, Toronto, Canada.
^2^  Institute of Medical Science, University of Toronto, Toronto, Canada.
^3^  Department of Medicine, University of Toronto, Toronto, Canada.
^4^  Division of Gynecologic Oncology, Princess Margaret Cancer Center, University Health Network, Toronto, Canada.
^5^  Department of Supportive Care, Princess Margaret Cancer Center, University Health Network, Toronto, Canada.

**Background/rationale or objectives/purpose**

Ovarian cancer (OC) is a common gynecologic cancer, with a 5-year survival rate of <25% for patients with advanced disease. Traumatic stress symptoms (TSS) may be severe, particularly at the time of diagnosis and recurrence. However, proactive psychotherapeutic care such as Managing Cancer And Living Meaningfully (CALM) is not routinely implemented as the standard of care in this setting. Herein we describe the protocol for a feasibility study of CALM implemented at the time of diagnosis and recurrence in patients with advanced OC.

**Methodology or Methods**

Patients ≥ 18 years with a new or recurrent advanced OC will be recruited from the Gynecologic Oncology Clinic at Princess Margaret Cancer Center in Toronto, Canada. Patients who provide informed consent will be offered 3–6 sessions of CALM over 3–6 months. CALM is a brief, supportive-expressive therapy that addresses challenges for patients with advanced cancer. Measures will be administered at baseline and at 3 and 6 months to assess traumatic stress, depression, death anxiety and perceived benefit of clinical care. Feasibility criteria include: >30% accrual of newly diagnosed and recurred patients approached over 12 months, ≥64% of participants completing > 3 sessions over 6 months, ≥64% completion of outcome measures at each timepoint and >50% of participants report perceived benefit based on score ≥ 14 on the Clinical Evaluation Questionnaire (CEQ).

**Impact on practice or results**

N/A—protocol.

**Discussion or Conclusions**

This study will assess the feasibility and optimal timing of implementing CALM at the time of either a new diagnosis or the recurrence of an advanced cancer in order to prevent and relieve traumatic stress in this population.

**92**

### 11.7. Efficacy of Accelerated Resolution Therapy in Cancer Care: A Two-Group Nonrandomized Pre–Post Pilot Study

Andrea Feldstain ^1,2^, Linda Carlson ^2^, Marie de Guzman Wilding ^1^ and Janet de Groot ^1,2^.

^1^  Tom Baker Cancer Center, Calgary, Canada.
^2^  Cumming School of Medicine, Calgary, Canada.

**Background/rationale or objectives/purpose**

An escalating demand for psychosocial services (Calgary, Canada) has been met with minimal resource increases. Innovation is necessary. Accelerated Resolution Therapy (ART) is an evidence-based, manualized psychotherapy using eye-movements. ART reduces the course of psychotherapy from >20 sessions to ≤5 transdiagnostically, with superior outcomes and sustainability. Evidence in oncology is lacking. In this oncology pilot study, we compare the single-session efficacy of ART versus evidence-based talking therapy (TALK) in symptom reduction.

**Methodology or Methods**

We used a nonrandomized pre–post design. Standard clinical measures were collected for existing patients between February 2023 and 2024, who had self-selected ART or TALK. Patient-Reported Outcomes (PROs) measured change in 6 symptoms (pain, fatigue, depression, anxiety, wellbeing and brain fog) pre- and post-session with the Edmonton Symptom Assessment System (ESAS). Within- and between-group changes were analyzed with repeated measures ANCOVAs.

**Impact on practice or results**

The participants (35 ART; 32 TALK) had various cancers and stages (59.7% in active treatment). Their ages ranged from 25–80 and 31.4% identified as male. The significant main effects were revealed for overall wellbeing (F(1,65) = 71.84, *p* < 0.001, ηp2 = 0.525) and brain fog (F(1,64) = 8.56, *p* = 0.005, ηp2 = 0.118). Significant interactions were revealed for pain (F(1,65) = 7.66, *p* = 0.007, ηp2 = 0.106), depression (F(1,62) = 10.15, *p* = 0.002, ηp2 = 0.141) and anxiety (F = 5.125, *p* = 0.027, ηp2 = 0.073). Score reductions favored ART.

**Discussion or Conclusions**

Our findings suggest similar or improved single-session PROs with ART. Further study is needed to compare PROs over a psychotherapy course and assess the maintenance of gains and differences in session quantity. If outcomes replicate non-cancer findings, expediting successful discharge may decrease waitlists while maintaining quality care.

**103**

### 11.8. Evaluating an Online Interprofessional Pain Management Group Intervention (ICM-PAC) for Survivors of Breast Cancer: A Feasibility Study

Karen Zhang ^1,2,3^, Greg Tippin ^2,4^, Jonathan Yen ^5^, Som Mukherjee ^1,6^, Greg Pond ^1,3^, Peter Ellis ^1,3^, Veronica Wong ^4^, Jonathan Sussman ^1,3^, Deb Evans ^1^, Denise Bryant-Lukosius ^1,3,7^ and Silva Darrouj ^1^.

^1^  Juravinski Cancer Center, Hamilton Health Sciences, Hamilton, Canada.
^2^  Department of Psychiatry and Behavioral Neurosciences, McMaster University, Hamilton, Canada.
^3^  Department of Oncology, McMaster University, Hamilton, Canada.
^4^  Michael G. DeGroote Pain Clinic, Hamilton Health Sciences, Hamilton, Canada.
^5^  Alan Edwards Pain Management Unit, McGill University, Hamilton, Canada.
^6^  Department of Oncology, Hamilton, Canada.
^7^  School of Nursing, McMaster University, Hamilton, Canada.

**Background/rationale or objectives/purpose**

Post-treatment chronic pain is common and often sub-optimally managed among breast cancer survivors. This proof-of-concept study will evaluate an online interprofessional program that combines psychosocial and exercise components. The objectives are to determine whether the intervention (1) is feasible and acceptable and (2) is associated with improved pain outcomes.

**Methodology or Methods**

This study is conducted at a regional cancer center in Southcentral Ontario. Participants are patients diagnosed with stages 1–3 breast cancer, past surgical excision, completed chemotherapy and/or radiation and currently experiencing bothersome persistent pain. Sample size (~*N* = 30) was determined based on the feasibility of conducting 3 treatment groups (6–10 participants each) over the course of 10 months.

Participants will enroll in a 6-session online pain management program that offers information and strategies to help patients cope with post-treatment pain using a biopsychosocial approach. The intervention will be delivered by nursing, psychology, physiotherapy and occupational therapy. Pre–post measures will assess patient acceptability, satisfaction and symptom outcomes. Study recruitment, data collection procedures, intervention adherence and intervention acceptability will be assessed to evaluate feasibility.

**Impact on practice or results**

Recruitment began in January 2024. The summary statistics will be presented to describe the sample characteristics. The content of the study intervention will be reviewed to gather feedback. The criteria to determine study acceptability and feasibility have been determined a priori and will be discussed.

**Discussion or Conclusions**

The results will inform the design of a future randomized controlled trial aimed at improving post-treatment pain management outcomed and quality of life for breast cancer survivors.

**113**

### 11.9. Adapting the Fear of Cancer Recurrence Therapy (FORT) for Young Adults with Lymphoma: What do Young Adults Have to Say?

Sharlane Lau ^1^, Rinat Nissim ^1^, Norma D’Agostino ^1^, Sophie Lebel ^2^, Christine Maheu ^3^, Madeline Li ^1^, Pamela Mosher ^4^, Marlie Smith ^5^, Jennifer Jones ^5^ and Aliza Panjwani ^1^.

^1^  Department of Supportive Care, Princess Margaret Cancer Center, Toronto, Canada.
^2^  Faculty of Social Sciences, University of Ottawa, Ottawa, Canada.
^3^  Ingram School of Nursing, McGill University, Montreal, Canada.
^4^  Department of Supportive Care, Princess Margaret Cancer Center.
^5^  Princess Margaret Cancer Center, Toronto, Canada.

**Background/rationale or objectives/purpose**

Fear of cancer recurrence (FCR) is a significant concern for young adult (YA) lymphoma survivors. Fear of Recurrence Therapy (FORT) is a cognitive-existential group intervention that effectively reduces FCR in breast and gynecological cancer populations, but has not yet been adapted for YA lymphoma survivors. The purpose of this study is to adapt FORT for YAs with Hodgkin’s or non-Hodgkin’s lymphoma (FORT-YA-L) and virtual delivery.

**Methodology or Methods**

We created an advisory board consisting of six YA patient partners (age range: 20–37; 3 females, 3 males; 4 with Hodgkin’s, 2 with non-Hodgkin’s) with diverse sociodemographic characteristics and treatment histories. Patient partners participated in a series of facilitated group meetings, reviewing FORT session-by-session and providing feedback on content, language, delivery and relevance to identity/diagnosis. Thematic analysis was used to analyze transcripts and findings will be used to facilitate the adaptation of FORT.

**Impact on practice or results**

The preliminary findings show that YA patient partners found FORT to be relevant and useful. To tailor FORT for the target population, they have provided valuable feedback to tailor the language and content to better reflect their developmental life stage (e.g., value domains that correspond with aspects of their identity) and diagnosis (e.g., triggers specific to lymphoma), as well as suggestions to improve the functionality of the patient manual.

**Discussion or Conclusions**

The current study fills an unmet need by tailoring FORT to address FCR in YA lymphoma survivors using a person-centered approach. Future work will include testing FORT-YA-L in a feasibility trial. These findings may be applicable to other YA hematological and cancer populations.

**124**

### 11.10. Sexual Adaptation Styles Amongst Men Following Prostate Cancer Treatment

Lauren M. Walker and Fatima I. Shah.
University of Calgary, Calgary, Canada.

**Background/rationale or objectives/purpose**

Sexual concerns after prostate cancer (PCa) treatment are high. Understanding ways in which patients and partners adapt to maintain sexual activity after PCa treatment is important to support and promote psychosocial outcomes for PCa survivors.

**Methodology or Methods**

We aimed to identify potential sexual adaptation styles that emerge following PCa treatment. Patients who had received PCa treatment completed an online survey to assess sexual practices and outcomes, as well as their perceptions of barriers and facilitators to adaptation. These were analyzed using a mixed thematic approach.

**Impact on practice or results**

Four distinct sexual adaptation styles emerged: Relationship Renegotiation and Sexual Renegotiation, which were couples-focused, and Acceptance/Resignation and Masturbation/Erection which were individual-focused. Successful adaptation as a couple was associated with sexual flexibility and a changed perspective on the expression of the relationship, wherein sexuality was acknowledged as one of several ways of maintaining intimate connection. The direct engagement of the partner facilitated the path to adaptation via renegotiation, allowing for the expansion of and redefinition of sexual expression, but relative emphasis on sexuality differed across couples-based adaptation styles. In contrast, successful adaptation as an individual focused on regaining erectile capacity and emphasizing a return to pre-treatment erectile function.

**Discussion or Conclusions**

Various sexual adaptation styles can be presented to patients (and couples) including insights and associated values to help promote proactive choices regarding adaptation in contrast to more reactive responding.

**125**

### 11.11. Evaluation of an Online versus In-Person Androgen Deprivation Therapy Educational Program for Prostate Cancer Patients

Lauren Walker ^1,2^, Carly Sears ^1^, Erik Wibowo ^3^, Andrew Matthew ^4^, Deborah MacLeod ^5^, John Robinson ^1^ and Richard Wassersug ^6^.

^1^  Division of Psychosocial Oncology, Department of Oncology at University of Calgary, Calgary, Canada.
^2^  Arnie Charbonneau Cancer Institute, at University of Calgary, Calgary, Canada.
^3^  Department of Anatomy, University of Otago, Dunedin, New Zealand.
^4^  University of Toronto, Department of Surgery, Division of Urology, Toronto, Canada.
^5^  Dalhouse University, Faculty of Health, School of Nursing, Halifax, Canada.
^6^  Cellular and Physiological Sciences, Faculty of Medicine, University of British Columbia, Vancouver, Canada.

**Background/rationale or objectives/purpose**

Androgen Deprivation Therapy (ADT) is the standard treatment for systemic prostate cancer (PCa). ADT is associated with many adverse effects that PCa patients are poorly prepared to manage. We thus developed a 1.5 h facilitated class as an evidence-based resource to help PCa patients maintain a good quality of life while on ADT (see (www.LifeOnAdt.com). Here we compare the experience of patients after attending online versus in-person classes.

**Methodology or Methods**

We used a mixed MANOVA design to assess potential differences among patients who attended online versus in-person classes in terms of (1) side-effects experienced, (2) self-efficacy to manage them and (3) bother associated with side-effects at baseline and approximately 10 weeks after attending the class.

**Impact on practice or results**

Both online and in-person classes are associated with a significant reduction in the severity of side-effect bother reported by PCa patients, suggesting the non-inferiority of online versus in-person formats. Side-effect bother decreased pre- to post-class but did not differ between in-person and online class cohorts. While self-efficacy to manage side-effects was slightly higher post-class in both formats, the increase was not statistically significant. Average self-efficacy ratings were significantly higher among in-person versus online class participants (*p* < 0.05; η_p_^2^ = 0.128).

**Discussion or Conclusions**

PCa patients who attended either the in-person or online ADT class were less bothered with ADT side-effects after participating in the program. As a more resource-conservative option, online classes offer greater accessibility, thereby reducing disparities in care.

**139**

### 11.12. Rx Narrative: A Proposal to Enhance Patient Experience among Those with Advanced Cancer

Janet de Groot ^1,2^, Paula Holmes-Redman ^3,4^ and Tom Rosenal ^5^.

^1^  University of Calgary, Cumming School of Medicine, Departments of Psychiatry, Oncology and Community Health Sciences, Calgary, Canada.
^2^  Tom Baker Cancer Center, Department of Psychosocial and Rehabilitation Oncology, Calgary, Canada.
^3^  Independent Researcher, Victoria, Canada.
^4^  All.CAN Knowledge Mobilization Working Group, Canada.
^5^  University of Calgary, Cumming School of Medicine, Calgary, Canada.

**Background/rationale or objectives/purpose**

Patients, informal caregivers and healthcare providers indicate that the inclusion of information about the patient’s personhood on the electronic medical record (EMR) enhances healthcare outcomes. The necessity of person-oriented care is particularly relevant amongst equity-deserving people who are living with advanced cancer. Numerous oncology appointments in often time-pressured settings may emphasize physical symptoms over the person. The Rx Narrative utilizes expressive writing by patients with advanced cancer to create key statements to be shared on the EMR to support communication and fuller personhood throughout cancer care.

**Methodology or Methods**

The multidisciplinary team will review literature about healthcare experiences of patients with advanced cancer, with an emphasis on equity-deserving groups experiencing barriers to care. The initial evidence-based literature on integrating patient narratives into the EMR will also be presented. Next, the facilitators will familiarize participants with the therapeutic use of expressive writing through experiential exercises. Small groups will brainstorm and report back to the larger group on best practices to integrate patient’s key statements into the EMR to ensure healthcare provider accessibility and uptake.

**Impact on practice or results**

At the end of the workshop, participants will be able to:

1. Identify an evidence-based rationale to include patients’ key statements about their core identities, legacy and/or values for inclusion in the EMR.
2. Describe how expressive writing contributes to identifying an individual’s core values and legacy experiences.
3. Plan for the inclusion of a patient’s key sentences into the EMR to enhance healthcare outcomes.

**Discussion or Conclusions**

Workshop outcomes will be documented to share with participants to support implementation in diverse settings.

**150**

### 11.13. Co-Creating Support: A Collaborative Approach to Developing a Parent Mental Health Intervention in Pediatric Oncology

Mélina Rivard ^1^, Zakaria Mestari ^1^, Léandra Desjardins ^2^, Julie Tramblay ^3^ and Élodie Bergeron ^3^.

^1^  UQAM, Montréal, Canada.
^2^  Sainte-Justine Hospital, Montréal.
^3^  LEUCAN, Montréal, Canada.

**Background/rationale or objectives/purpose**

Despite the development of various psychosocial interventions in pediatric oncology, there remains a lack of consensus on delivery modalities, program content, as well as approaches and strategies (Ogez et al., 2019). Furthermore, family members’ perspectives are rarely integrated into the development and evaluation of interventions (Beeler et al., 2023).

**Methodology or Methods**

To address this need, a collaborative approach was undertaken by LEUCAN (a large community pediatric cancer organization), university researchers and families to co-develop a parent mental health support intervention, which was evaluated using a rigorous and planned research process. Specifically, this participative research project consists of three phases.

**Impact on practice or results**

First, it aims to identify psychosocial intervention needs by conducting a survey among over 400 families with children diagnosed with cancer, reviewing the scientific literature and assessing the existing services available to parents. Second, its goal is to collaboratively develop an intervention by and for stakeholders, integrating both evidence-based practices and genuine family needs. Finally, the third objective is to pilot the resulting intervention in a clinical study and evaluate its effects on parental well-being.

**Discussion or Conclusions**

The objective of this presentation is to share the outcomes of the second phase. Specifically, it will outline the findings of the qualitative thematic analysis involving 21 participants (parents, practitioners and researchers) who participated in the process that enabled the development of the intervention. Additionally, we will present the resultant intervention model, with a specific focus on its curriculum and the themes it should encompass, facilitators’ and participants’ characteristics, impact mechanisms, as well as the program’s format and duration.

## 12. Final Category: L. Palliative and End-of-Life Care

**6**

### 12.1. Palliative and End-of-Life Care

Ifeyinwa Carol Onwukeme, Kelechi Uzochukwu Nneke, Joseph Chimezie Onwukeme and John Ubale Gulee.
Sali Hoe Foundation, Abuja, Nigeria.

**Background/rationale or objectives/purpose**

When treatment for cure is not possible and the only option is palliative and end-of-life care, the patient is said to be terminally ill.

The word palliative in Latin means “caring”. This is the total care of patients whose conditions do not respond to curative treatment; sometimes hospice care and palliative end-of-life care have similar goals. However, hospice care is specifically for terminally ill patients while palliative and end-of-life care is offered to patients whose conditions are not necessarily terminal. Palliative begins when illness is diagnosed and continues throughout the duration of the treatment.

**Methodology or Methods**

Nigeria has an estimated population of 220,542,029 and there are large numbers of patients needing palliative and end-of-life care.

Current statistics show that the health institutions rendering health care in Nigeria are 33,303 general hospitals, 20,278 primary health care centers and 59 teaching hospitals and federal medical centers. About 10,655 are private hospitals. Unfortunately, we do not have enough palliative and end-of-life care centers.

**Impact on practice or results**

According to the global directory of palliative and end-of-life care institutions and organizations, Nigeria has 10 centers offering palliative care in the country. About two of these centers are situated in the western part of Nigeria which provides care services.

**Discussion or Conclusions**

There is a compulsory need for the introduction of adequate and functional palliative care in a nation’s health care system for patients with advanced disease and a limited life expectancy to have quality in life and at the end of life.

**39**

### 12.2. Prognostic Beliefs in Patients with Advanced Cancer Receiving Outpatient Palliative Care

Brooke D’Mello ^1,2^, Rebecca Wong ^1,3^, Kenneth Ma ^1^, Athena Li ^2^, Camilla Zimmermann ^1,4,5^, Breffni Hannon ^1,4^, Melissa Miljanovski ^1^ and Gary Rodin ^1,5,6^.

^1^  Department of Supportive Care, Princess Margaret Cancer Center, University Health Network, Toronto, Canada.
^2^  Faculty of Health Sciences, McMaster University, Hamilton, Canada.
^3^  Faculty of Health Sciences, Western University, London, Canada.
^4^  Division of Palliative Medicine, Department of Medicine, Toronto, Canada.
^5^  Department of Psychiatry, University of Toronto, Toronto, Canada.
^6^  Global Institute of Psychosocial, Palliative and End-of-Life Care, University Health Network, Toronto, Canada.

**Background/rationale or objectives/purpose**

Prognostic awareness (PA) refers to the understanding that patients with advanced disease have of their prognosis. We instead propose the term “prognostic beliefs” (PB), since PA implies there is a knowable truth about survival. We aim to assess the PB of patients with advanced cancer and its relationship to their i. recall of prognosis communicated by their physicians, ii. physical and psychological well-being and iii. actual survival.

**Methodology or Methods**

In total, 188 patients with advanced cancer receiving outpatient palliative care completed the Prognostic Beliefs Questionnaire, Satisfaction with Life Questionnaire, Patient Health Questionnaire, Quality of Life at the End-of-Life Cancer Scale and Condensed Memorial Symptom Assessment Scale. PB group comparisons on physical and psychosocial well-being were conducted using one-way ANOVA.

**Impact on practice or results**

Overall, 88.3% of patients recalled discussing treatability/curability with their physicians and 41.0% recalled discussing survivability. For treatability/curability prognoses, 86.4% of patients had PB concordant with their physicians’, 12.9% had more optimistic PB and 0.7% had more pessimistic PB. For survival prognoses, 57.1% had PB concordant with their physicians’, 32.5% had more optimistic PB and 10.4% had more pessimistic PB. In patients who had since died, 47.0% had accurate survival PB, 16.7% had more optimistic PB and 36.4% had more pessimistic PB. No significant associations were observed between PB and psychosocial or physical well-being.

**Discussion or Conclusions**

Individuals with advanced cancer receiving specialized palliative care largely possess PB concordant with physicians’ communicated prognoses regarding treatability/curability and, to a lesser extent, survival. Incorporating PB as a construct may enhance the clarity of research in this domain.

**60**

### 12.3. Developing the ‘Understanding Palliative Care’ Module: Incorporating Public, Patient and Family Caregiver Perspectives

Patricia Biondo ^1,2^, Mary-Ann Shantz ^2^, Yuanjie Zheng ^2^, Miranda Manning ^2^, Maril Murray ^2^ and Louise Kashuba ^2^.

^1^  University of Calgary, Calgary, Canada.
^2^  Covenant Health Palliative Institute, Edmonton, Canada.

**Background/rationale or objectives/purpose**

Improving public awareness of palliative care is crucial for improving access to, and uptake of, palliative care, which has demonstrated benefits for patients and health systems. However, there is a lack of engaging, accessible educational palliative care resources designed for public audiences.

**Methodology or Methods**

After conducting a literature review and environmental scan to identify leading international resources to adapt and promote in Alberta, we developed “*Understanding Palliative Care*”, an innovative, online educational module incorporating best practices for defining and promoting palliative care to a public audience. An expert working group with representation from nursing, medicine, social work, instructional design and care navigation advised on the development of the module. Incorporating the perspectives of Albertans with lived palliative care experience was deemed essential by the working group. We identified three Albertans (one patient and two family caregivers) of diverse ages and cultural backgrounds who had personally benefitted from palliative care and consented to record virtual interviews. We incorporated multiple interview segments into the module that highlight the physical, emotional, social and spiritual supports provided by palliative care. Finally, a panel of thirteen public volunteers provided feedback on the content, design and navigation of the draft module.

**Impact on practice or results**

The “*Understanding Palliative Care*” module fills an important gap in Alberta, providing a free, online, evidence-based and engaging public-facing educational tool to improve public awareness and understanding of palliative care.

**Discussion or Conclusions**

Featuring Albertans in the module with lived palliative care experience allows module users to connect with and understand tangible benefits of this important area of care.

**99**

### 12.4. Psychosocial–Spiritual Alberta Series: A Partnership in Palliative and Grief Education for Psychosocial Clinicians

Sheila Killoran, Ellen Mi, Patricia Biondo, Maril Murray and Louise Kashuba.
Covenant Health, Edmonton, Canada.

**Background/rationale or objectives/purpose**

Clinicians providing psychosocial and spiritual support across oncology, medical, palliative or grief care settings may be working in isolation or as the only member of their discipline on a team. There is often a lack of accessible education on the psychosocial and spiritual aspects of palliative and grief care for clinicians providing these services. While psychosocial and spiritual care is a cornerstone in palliative and grief care, we need to enhance clinician education in this area and foster connections between clinicians providing this vital work.

**Methodology or Methods**

The *Psychosocial–Spiritual Alberta Series* is designed for social workers, spiritual care providers, creative arts therapists, occupational therapists, Indigenous liaisons, counsellors, psychologists, nurses and other clinicians providing direct emotional and spiritual support in palliative and grief settings. A collaboration between Pallium Canada and the Covenant Health Palliative Care Institute, this newly developed monthly webinar series provides education on topics including competency frameworks, complex grief, self-care, non-physical suffering, legacy work, spiritual practices and mindfulness. Specialist clinicians provide presentations and lead break-out room discussions to deepen participant learning.

**Impact on practice or results**

The *Psychosocial–Spiritual Alberta Series* received an overwhelmingly positive response, with 90+ registrants per session from rural and urban care settings and across diverse disciplines. The creation of an Alberta Community of Practice was identified as a next step to further the collaboration and learning opportunities between psychosocial-spiritual professionals.

**Discussion or Conclusions**

This series can serve as a model for other provinces or regions to provide accessible education opportunities and build connections between clinicians providing psychosocial and spiritual support in palliative, oncology and grief care.

**110**

### 12.5. Managing Cancer and Living Meaningfully (CALM): A National Implementation Program to Enhance the Wellbeing of Patients and Families Living with Advanced Cancer

Laura Foran ^1^, Carmine Malfitano ^2^ and Gary Rodin ^2,3^.

^1^  Princess Margaret Cancer Center, Toronto, Canada.
^2^  The Princess Margaret Cancer Center, University Health Network, Toronto, CA, Toronto, Canada.
^3^  The University of Toronto, Toronto, CA, Toronto, Canada.

**Background/rationale or objectives/purpose**

Individuals facing advanced cancer face a set of predictable challenges that cause profound physical and psychological distress in a substantial proportion of these individuals. Managing Cancer and Living Meaningfully (CALM) is a brief, evidence-based psychotherapeutic intervention developed to decrease or prevent distress and increase well-being in this population. A robust, international program of research demonstrated its effectiveness and feasibility. While many individuals across the world have benefited from CALM, it has not yet been systematically implemented as part of routine cancer care. With philanthropic support, we have launched a national Canadian program to implement CALM for patients and families facing advanced cancer in cancer centers across Canada.

**Methodology or Methods**

A 3-phase plan spanning over 4.5 years will aim to establish sustainable CALM clinics in each of the cancer centers. Steps in this process will include the training of therapists and supervisors of CALM by means of a digital training platform and virtual supervision and the establishment of mechanisms for the routine and proactive referral of patients. Prospective CALM clinicians and supervisors will be healthcare professionals already embedded in each center (e.g., social workers and nurses).

**Impact on practice or results**

This program has the potential to enhance the wellbeing of individuals with advanced cancer across Canada and can serve as a template for the application of the CALM approach to the care of patients with other life-limiting major medical conditions and for the support of the parents of children with cancer.

**122**

### 12.6. Advanced Cancer Support in Virtual Rehabilitation and Exercise in North Zone (ACTIVE-North): A Feasibility Study

Sonya Lowe ^1^, Christopher Sellar ^2^, Petra Grendarova ^3^, Naomi Dolgoy ^1^ and Margaret McNeely ^1^.

^1^  University of Alberta, Edmonton, Canada.
^2^  Cancer Rehabilitation Clinic, Edmonton, Canada.
^3^  Vancouver Island Cancer Center, Victoria, Canada.

**Background/rationale or objectives/purpose**

Advanced cancer patients in remote and rural areas lack equitable access to interdisciplinary, individualized support in rehabilitation and therapeutic exercise to optimally address cancer-related fatigue and loss of physical functioning. The aim of this study was to determine the feasibility of a rehabilitation and therapeutic exercise intervention which is delivered virtually by an interdisciplinary team for advanced cancer patients in Alberta Health Services (AHS) North and Central Zones.

**Methodology or Methods**

Twenty adult participants were recruited from AHS Community Cancer Centers and Palliative Care teams. All study components were delivered via secure live Zoom sessions in patients’ homes. A single group pre-to-post-test study design was used, consisting of an individualized 8-week program of one-to-one exercise sessions, one-to-one consultations with a rehabilitation specialist and weekly one-to-one meetings with a palliative care physician. The Edmonton Symptom Assessment System (ESAS-r), Brief Fatigue Inventory (BFI), Lower Extremity and Upper Extremity Functioning Scales, Sit-to-Stand, Sit-and-Reach, Shoulder Range of Motion, 4-Stage Balance Test and 2 Minute Step Test (2MST) were conducted pre- and post-intervention.

**Impact on practice or results**

The primary feasibility outcome of the completion rate was 13/20 (65%), retention rate 13/20 (65%) and adherence rate 93%. No adverse events were reported. Of the 13 participants who completed the study, the majority reported improvement in the 2MST, ESAS-r global distress and BFI total scores.

**Discussion or Conclusions**

The delivery of an interdisciplinary, individualized virtual rehabilitation and therapeutic exercise intervention is feasible in advanced cancer patients who reside in remote and rural areas. A larger trial is warranted to elucidate potential benefits in this population.

**128**

### 12.7. Five Poems on the Experience of Digital Storytelling with Advanced Cancer

Jessame Gamboa ^1^, Kathleen Sitter ^2^, Janet de Groot ^1,3,4^ and Carly Sears ^3^.

^1^  University of Calgary, Dept. of Psychiatry, Calgary, Canada.
^2^  University of Calgary, Faculty of Social Work, Calgary, Canada.
^3^  University of Calgary, Community Health Sciences Department, Calgary, Canada.
^4^  Tom Baker Cancer Center, Dept. of Psychosocial and Rehabilitation Oncology, Calgary, Canada.

**Background/rationale or objectives/purpose**

In the health care setting, digital storytelling provides patients with an opportunity to express their personal stories via digital devices. The process involves creating short multimedia videos using photographs, images and personal narratives. The technique has gained significant momentum within various healthcare settings, particularly in the area of cancer. This study had two aims: (1) to explore the experiences and perspectives of adults with advanced cancer in terms of creating their digital stories and (2) to understand the ways in which digital storytelling may facilitate meaning-making related to the cancer experience.

**Methodology or Methods**

Six participants from the evidence-based Managing Cancer and Living Meaningfully (CALM) pilot trial in Southern Alberta completed the digital storytelling intervention.

One-on-one interviews were conducted with each participant after he/she created a digital story. All interviews were audio recorded and transcribed. Patients’ words were arranged and rearranged to create a poem using Evonne Miller’s ICCEE approach, known as poetic inquiry.

**Impact on practice or results**

Five found poems were constructed from the interview transcripts. A number of themes were generated based on an analysis of poems. Themes include a sense of pride, newness, satisfaction, purpose in helping others, legacy and trust.

**Discussion or Conclusions**

The findings offer a unique view into the personal storytelling experiences of adults with advanced cancer, thereby providing evidence of digital storytelling’s utility in this clinical population. Notably, this study illustrates the benefits of using poetic inquiry to derive rich insights into the experiences of adults with advanced cancer in a way that evokes deeper, nuanced understanding and empathy.

**138**

### 12.8. An Implementation Study of the Managing Cancer and Living Meaningfully (CALM) Psychosocial Intervention in Southern Alberta

Janet de Groot ^1,2^, Carly Sears ^3^, Fay Strohschien ^4^, Kristen Silveira ^5^, Jessame Gamboa ^6^ and Andrea Feldstain ^7,8^.

^1^  Arthur Child Comprehensive Cancer Center, Calgary, Canada.
^2^  University of Calgary, Department of Psychiatry, Oncology and Community Health Sciences, Calgary, Canada.
^3^  University of Calgary, Department of Community Health Sciences, Calgary, Canada.
^4^  University of Calgary, Faculty of Nursing, Calgary, Canada.
^5^  Tall Tree Integrated Health, Victoria, Canada.
^6^  University of Calgary, Department of Psychiatry, Calgary, Canada.
^7^  Tom Baker Cancer Center, Department of Psychosocial and Rehabilitation Oncology, Calgary, Canada.
^8^  University of Calgary, Department of Oncology, Calgary, Canada.

**Background/rationale or objectives/purpose**

A pragmatic implementation study was carried out to guide the integration of the evidence-based Managing Cancer and Living Meaningfully (CALM) intervention for patients with advanced solid-tumor cancers into established psychosocial programming and a palliative care program in Southern Alberta. Patients are encouraged to include a close other in one or more CALM sessions.

**Methodology or Methods**

Referral routes for participants to the CALM intervention study included (a) direct referrals by oncology clinicians, (b) indirect referrals (patients referred to tumor site-specific psychosocial clinicians were offered the CALM intervention as an alternative) and (c) self-referral. Recruitment feasibility was assessed via enrollment rates. Evaluation of intervention acceptability included the proportion of consented patients participating in ≥3 CALM sessions and the proportion that included a close other in ≥1 session. Effectiveness of CALM was assessed for its capacity to contribute to reduced depression levels (PHQ9 questionnaire).

**Impact on practice or results**

Of 141 patients referred to the CALM study, 48.9% (n = 69) completed informed consent for study participation. Of this group, 59.4% (n = 41) completed three CALM sessions and 39.1% (n = 27) included a close other in at least one session. After three months, the mean PHQ9 for participants showed a significant reduction (*p* < 0.05).

**Discussion or Conclusions**

The feasibility and acceptability for the integration of CALM with the benefit of reducing depression was shown across the established psychosocial and palliative care programs. Future directions may include routinely offering the CALM intervention for all patients with non-curative cancers within weeks of their diagnosis. This would require training additional clinicians for competence to provide CALM.

**140**

### 12.9. Exploring Patient-Specific Factors and Personal Reflections about the Managing Cancer and Living Meaningfully (CALM) Intervention for Adults with Advanced Cancer in Southern Alberta: A Mixed Methods Study

Carly Sears ^1^, Scott Patten ^1^, Andrea Feldstain ^2,3^, Jessica Simon ^1,2,4^, Fay Strohschein ^5^, Kristen Silveira ^6^ and Janet de Groot ^1,2,3^.

^1^  University of Calgary, Community Health Sciences Department, Calgary, Canada.
^2^  University of Calgary, Oncology Department, Calgary, Canada.
^3^  Tom Baker Cancer Center, Dept. of Psychosocial and Rehabilitation Oncology, Calgary, Canada.
^4^  Palliative and End of Life Care, Alberta Health Services, Calgary, Canada.
^5^  University of Calgary, Faculty of Nursing, Calgary, Canada.
^6^  Island Health—Vancouver Island Health Authority, Victoria, Canada.

**Background/rationale or objectives/purpose**

The evidence-based Managing Cancer and Living Meaningfully (CALM) psychotherapeutic intervention was designed to address the complex needs of those with advanced cancer. Ample evidence supports the efficacy of CALM therapy; less is known about the patient-specific factors that influence the initiation and continuation of CALM sessions. This project’s aim is to refine our understanding of the referral route and timing and patient-specific factors associated with participation in CALM therapy.

**Methodology or Methods**

Adults with advanced (Stage IV or Stage III recurrent), solid-tumor cancers were recruited for a CALM pilot trial via the Tom Baker Cancer Center (Calgary), the Southwest Palliative Care Team (Lethbridge) and through community cancer care organizations (Calgary). Participants (*n* = 69) who consented to follow-up (*n* = 24) were invited to participate in Zoom-based interviews about their CALM experience (n = 10). A concurrent triangulation mixed-methods design was used. Within an Interpretive Description framework, interview responses were analyzed and triangulated with results of non-parametric analysis of baseline questionnaire data from all participants.

**Impact on practice or results**

The triangulation of quantitative and qualitative results suggests that the initiation and continuation of CALM sessions is affected by multiple, complex factors including mood symptoms, the referral route, the need for support outside of family/friends and a decline in health status over time.

**Discussion or Conclusions**

Overall, the findings support the value of providing comprehensive, sensitively worded information about CALM shortly after an advanced cancer diagnosis is made. Patients prefer autonomy in choosing whether and when to initiate CALM counseling.

**141**

### 12.10. Exploring Patient Experiences with the Managing Cancer and Living Meaningfully (CALM) Intervention: A Qualitative Study Using Interpretive Description

Carly Sears ^1^, Andrea Feldstain ^2,3^, Fay Strohschein ^4^, Jessica Simon^1,2,5^, Scott Patten ^1^, Kristen Silveira ^6^ and Janet de Groot ^1,2,3^.

^1^  University of Calgary, Community Health Sciences Department, Calgary, Canada.
^2^  University of Calgary, Oncology Department, Calgary, Canada.
^3^  Tom Baker Cancer Center, Department of Psychosocial and Rehabilitation Oncology, Calgary, Canada.
^4^  University of Calgary, Faculty of Nursing, Calgary, Canada.
^5^  Palliative and End of Life Care, Alberta Health Services, Calgary, Canada.
^6^  Island Health—Vancouver Island Health Authority, Victoria, Canada.

**Background/rationale or objectives/purpose**

The integration of oncology, palliative and psychosocial care is associated with numerous benefits for patients and their loved ones. The evidence-based Managing Cancer and Living Meaningfully (CALM) intervention may help to bridge these traditionally separate silos of care. This qualitative study thus sought to explore CALM patients’ perceptions of (1) diagnosis and living with advanced cancer, (2) referral to and engagement in CALM therapy and (3) impacts of CALM on their navigation of oncology and palliative care.

**Methodology or Methods**

Adults with advanced (Stage IV or III recurrent) solid-tumor cancers were recruited via the Tom Baker Cancer Center, the Southwest Palliative Care Team and through community cancer care organizations. Participants from an ongoing CALM pilot trial who consented to follow-up were invited to participate in brief virtual interviews. Ten women with diverse advanced cancer diagnoses participated. Within an Interpretive Description framework, thematic analysis was used to analyze interview responses.

**Impact on practice or results**

The CALM patients discussed a variety of ways in which CALM therapy supported their navigation of healthcare systems and life with advanced cancer. The interviewees described a sense of support with (1) self-advocacy as a patient, including communication with care team members and treatment decision making; (2) understanding palliative care as a resource to enhance quality of life beyond the end-of-life period; and (3) shifting perspectives on living well with advanced cancer.

**Discussion or Conclusions**

CALM counseling may enhance the integration of psychosocial, palliative and oncology care by supporting patients as they navigate the complexities of living with advanced cancer, including relationships with oncology and palliative care teams.

**146**

### 12.11. Psychosocial Correlates of Death Anxiety in Advanced Cancer: A Scoping Review

Tyler Brown and Justin Sanders.
McGill University, Montreal, Canada.

**Background/rationale or objectives/purpose**

Individuals living with advanced cancer commonly experience death anxiety, which refers to the distressing thoughts or feelings associated with an awareness of one’s mortality. Identifying the psychological and social factors linked to death anxiety may inform conceptual models, clinical screening and intervention strategies in oncology and palliative care settings. Accordingly, the present scoping review was conducted to assess and summarize the literature on the psychosocial correlates of death anxiety among individuals with advanced cancer.

**Methodology or Methods**

A comprehensive scoping review methodology was used following the Arksey and O’Malley framework. A literature search was conducted using four electronic databases: CINAHL, Embase, PsycInfo and MEDLINE.

**Impact on practice or results**

Sixteen studies met the inclusion criteria. Seventeen psychosocial correlates of death anxiety were identified. The most frequently investigated correlates included depression, spiritual well-being and attachment security.

**Discussion or Conclusions**

This review provides a current summary of psychosocial factors associated with death anxiety in advanced cancer. Multiple psychosocial correlates should be targeted concurrently in research and clinical practice to address death anxiety.

**151**

### 12.12. Death Anxiety among Patients with Advanced Cancer Receiving Outpatient Palliative Care: An Exploratory Analysis

Emily Lau ^1,2^, Melissa Miljanovski ^3^, Camilla Zimmermann ^1^, Breffni Hannon ^3^, Kenneth Mah ^3^ and Gary Rodin ^1,2^.

^1^  Princess Margaret Cancer Center, Toronto, Canada.
^2^  Dalla Lana School of Public Health, University of Toronto, Toronto, Canada.
^3^  Princess Margaret Cancer Centre Melissa Miljanovski1, Toronto, Canada.

**Background/rationale or objectives/purpose**

This study investigated the prevalence and correlates of death anxiety in outpatients with advanced cancer receiving specialized palliative care.

**Methodology or Methods**

Patients with advanced cancer were recruited from an outpatient palliative care clinic in a comprehensive cancer center. Death anxiety was measured with the Death and Dying Distress Scale (DADDS). Exploratory analyses examined univariable associations of death anxiety with variables that included religion, gender, primary language, education level, household income, living arrangement and duration of disease and of palliative care. Significant univariate correlates were then entered into multiple linear regression analyses.

**Impact on practice or results**

Among the 150 participants included in this secondary analysis, moderate-to-severe levels of death anxiety (DADDS score ≥ 25) were reported by 43.3% of participants. When significant correlates in the univariable analysis were entered into the multivariate analysis, a low household income (i.e., <CAD 30,000) and living alone were significantly correlated with a higher DADDS total score (*p* < 0.01 and *p* = 0.02, respectively).

**Discussion or Conclusions**

More than one third of patients with advanced cancer receiving specialized palliative care report moderate-to-severe death anxiety. Those who live alone and with less economic advantage are at the greatest risk of this outcome. These findings suggest the need for more specific interventions in patients with advanced diseases to prevent or alleviate death anxiety, including attention to its social determinants.

**152**

### 12.13. The Clinical Access Responsibility and Ethics in the Desire for Death (CARED) International Task Force on Assisted Dying: Insights from the Muddy Middle

Eugenia Ubeira Amaral ^1^, Gilla Shapiro ^2^, Gary Rodin ^1^ and Madeline Li ^3^.

^1^  Department of Supportive Care, Princess Margaret Cancer Center, University Health Network, Toronto, Canada.
^2^  Department of Supportive Care, Princess Margaret Cancer Center, Toronto, Canada.
^3^  Department of Supportive Care, Princess Margaret Cancer Center, University Health Network.

**Background/rationale or objectives/purpose**

Assisted dying has been legal for decades in America and European countries and more recently in Canada and other global jurisdictions. There is notable heterogeneity in the practice of MAiD with different challenges experienced by different contexts. Accordingly, the Clinical Access Responsibility and Ethics in the Desire for Death (CARED) taskforce was created to generate and disseminate knowledge and to advance research, health policies, education and clinical practice regarding assisted dying in diverse settings.

**Methodology or Methods**

A group of nine international experts in palliative care, mental health, law, ethics, public health and sociology have convened with the goal of developing balanced writing to address issues related to assisted dying. The first step is to create a global assisted dying dashboard to elucidate how various legislations have affected assisted dying practices over time. Euthanasia and assisted suicide metrics have been analyzed from all published reports across 9 countries.

**Impact on practice or results**

Balanced writing about the cultural and anthropological context of assisted dying, the legal, ethical and clinical tensions, the psychological dimensions of the desire for death and the sociological consequences will support high-quality assisted dying practice.

**Discussion or Conclusions**

There is a debate about the balance between providing equitable access to MAID and protecting those with modifiable vulnerabilities. Some advocate that such vulnerabilities should not exclude individuals from accessing MAiD. What is missing in this debate are academic voices that adopt a “muddy middle” approach that takes into account a non-advocacy clinical lens. The CARED project will provide this lens.

**154**

### 12.14. A Qualitative Study of Suffering and Depression in Patients with Advanced Cancer Requesting MAiD

Stefan Aguiar ^1,2^, Aliza Panjwani ^1,2^, Gilla K. Shapiro ^1,2,3^, Olivia Mazzurco ^1,2^, Emma Hagopian ^1^, Robin Graham ^1^, Anne Barbeau ^1^, Anne Rydall ^1^, Gary Rodin ^1,2,3^ and Madeline Li ^1,2^.

^1^  Princess Margaret Cancer Centre—University Health Network, Toronto, Canada.
^2^  University of Toronto, Toronto, Canada.
^3^  Global Institute of Psychosocial, Palliative and End-of-Life Care (GIPPEC), Toronto, Canada.

**Background/rationale or objectives/purpose**

Terminally ill patients with depression are more likely to request medical assistance in dying (MAiD) than those without depression. Depression can result in psychological biases that may alter affective forecasting, resulting in poor predictions of the future and impairments in capacity. The goal of this study was to understand how cognitive and emotional factors affect decision making among depressed and non-depressed patients with advanced cancer who have requested MAiD.

**Methodology or Methods**

Qualitative interviews (n = 10) were conducted with patients diagnosed with advanced cancer, including those with (n = 3) and without (n = 7) moderate or severe depressive symptoms, of which all requested MAiD. Transcripts were coded using a grounded theory approach to explore how cognitive and emotional factors influenced attitudes and decision making about MAiD as well as forms of psychological suffering.

**Impact on practice or results**

Psychological suffering was expressed as fear related to losing capacity or distress around conflicting desires (desire for hope vs accepting the inevitability of death). Patients described challenges, decision-making values (quality vs quantity of life), approaches to managing uncertainty and possible mortality, as well as unhelpful and beneficial aspects of support. Thematic comparisons between depressed and non-depressed participants are currently underway.

**Discussion or Conclusions**

The study’s findings can inform the design of psychotherapeutic interventions and MAiD capacity assessments for patients with significant psychological suffering or depression.

## 13. Final Category: M. Pandemics and Cancer Care Issues

**8**

### 13.1. Capturing Key Attributes of Cancer Team Functioning during and beyond COVID-19 Using Mixed-Methods

Samar Attieh ^1^ and Carmen G. Loiselle ^2,3^.

^1^  Division of Experimental Medicine, Faculty of Medicine and Health Sciences, McGill University, Montreal, QC, Canada;
^2^  Department of Oncology and Ingram School of Nursing, Faculty of Medicine and Health Sciences, McGill University, Montreal, QC, Canada.
^3^  Segal Cancer Center, Jewish General Hospital, CIUSSS Centre Ouest, Montreal, QC, Canada.

**Background/rationale or objectives/purpose**

During COVID-19, cancer teams faced significant work-related challenges due to rapid shifts in practices, burnout and workforce shortages. Key indicators of team functioning include team effectiveness (TE) and relational coordination (TRC). This mixed-method study aimed to (1) explore multi-stakeholder perceptions of team functioning during COVID-19 (T1) and beyond (T2), (2) assess if patient perceptions of TE/TRC were significantly associated with their cancer care experiences and (3) identify key attributes of optimal team functioning.

**Methodology or Methods**

Sixty-six participants representing four groups (i.e., 13 healthcare professionals (HCPs), 40 patients, 6 informal caregivers and 7 volunteers) were recruited from two outpatient cancer clinics at a University-affiliated hospital, in Montreal, QC, and invited to complete e-measures at T1 (2021–2022) and T2 (2023). A subgroup (n = 13) also participated in separate fuzzy cognitive mapping e-sessions.

**Impact on practice or results**

At T1, the TE and TRC ratings (scale from one (low) to five (high)) were high with means of M = 4.47 and 3.77, respectively. The perceptions were relatively unchanged at T2 with no significant differences found across the four stakeholder groups. Patient perceptions of TE/TRC were significantly and positively correlated with cancer care experiences (r_s_ ranging from 0.69 to 0.83; *p* < 0.01). A shared mental model—created by combining stakeholders’ maps—showed the strongest attributes of optimal team functioning: effective communication, enhanced cancer care experiences, care ethos (e.g., empathy, compassion and respect), psychosocial support for patients and HCPs and team role clarity.

**Discussion or Conclusions**

The findings provide quantitative and qualitative insights into optimal team functioning components that can inform future team preparedness when facing new pandemics.

**71**

### 13.2. Cancer Recovery Narratives Pre- and Peri-COVID-19: A Mixed-Methods Comparative Analysis

Lucas Norton ^1^, Karen Fergus ^1,2^, Iana Ianakieva ^1^, Erin Gross ^2^, Benjamin Stevenson ^1^, Catriona Buick ^1^ and David Olson ^1^.

^1^  York University, Toronto, Canada.
^2^  Sunnybrook Health Sciences Center, Toronto, Canada.

**Background/rationale or objectives/purpose**

The COVID-19 pandemic resulted in significant reductions in access to treatment, creating a backlog which continues to impact cancer care. Moving into the recovery phase of treatment is often distressing, as patients confront fears of recurrence, functional limitations and changes in identity. The current study examines the effectiveness and emergent themes of a brief Narrative Care Intervention (NCI) to support individuals during the post-treatment juncture with data collected at pre- and peri-COVID-19 timepoints.

**Methodology or Methods**

Data from both the pre- (n = 27) and peri-COVID-19 (n = 27) groups are compared using a mixed-methods concurrent triangulation approach. Quantitative analyses examine the effectiveness of the narrative intervention through measures taken before and after the NCI. Thematic analysis coding is employed to discern qualitative areas of commonality and difference between the two groups. Research assistants, who are blinded to the pre- and peri-COVID-19 conditions of the study, then apply this coding system to the transcripts.

**Impact on practice or results**

Understanding the impact that the pandemic has had on cancer survivors will generate new knowledge surrounding the needs arising for individuals treated during the peri-COVID-19 period and guide the development of targeted interventions for individuals struggling at the end of treatment. Emerging qualitative themes specific to the peri-COVID-19 period include loneliness, difficulty locating/accessing support and concerns surrounding delayed detection/treatment.

**Discussion or Conclusions**

This study demonstrates the potential benefits of an NCI and describes what has changed experientially for patients since COVID-19 began. Reported experiences of loneliness and difficulty accessing support reinforce the importance of appropriate psychosocial interventions for patients at the post-treatment juncture.

## 14. Final Category: N. Patient-Oriented Research Approaches

**13**

### 14.1. Lessons from Stakeholder Engagement: Two Models for Involving Stakeholders in Research

Julie Easley ^1^ and Margaret Fitch ^2^.

^1^  Horizon Health Network, Chalmers Regional Hospital, Fredericton, Canada.
^2^  Bloomberg Faculty of Nursing, University of Toronto, Toronto, Canada.

**Background/rationale or objectives/purpose**

A growing body of evidence supports the notion that involving stakeholders in research has benefits. Various levels of engagement and roles for stakeholders have been described. However, there remains a lack of consensus about the best way to accomplish successful engagement. The purpose of this presentation is to present the lessons learned from two models of engaging cancer survivors in research projects: CanIMPACT and PRO-ACTIVE.

**Methodology or Methods**

Two research projects involved stakeholders over a period of 5 years and gathered feedback from the stakeholders about their experiences and the outcomes for the respective studies. Qualitative descriptive analysis of the feedback facilitated identification of relevant lessons about how to engage stakeholders effectively.

**Impact on practice or results**

CanIMPACT engaged survivors and family members in a stakeholder advisory group. PRO-ACTIVE engaged survivors/family caregivers, allied health clinicians, physicians and surgeons and policy executives in individual focus groups. Key lessons included (1) the need for recruiting relevant stakeholders, (2) the need for ensuring understanding of the stakeholder role and responsibility, (3) the value of instruction about research and the topic area, (4) the benefit of specific and meaningful requests to the stakeholders, (5) the importance of regular communication between the stakeholders and the investigators and (6) the value of an on-going evaluation during the engagement process.

**Discussion or Conclusions**

Stakeholder engagement requires the intentional planning, implementation and evaluation of processes to facilitate meaningful and respectful involvement. Input from stakeholders can influence the patient-centeredness and effectiveness of research questions and approaches.

**15**

### 14.2. Using the Cancer Experience Measurement Framework to Guide Psychosocial Oncology Practice and Research

Carmen G. Loiselle.
McGill University, Montréal, Canada.

**Background/rationale or objectives/purpose**

Much of psychosocial oncology work focuses on specific facets of cancer-related experiences rather than a more comprehensive approach involving both personal and contextual factors. More delineated depictions of diversity in experiences are necessary to further guide psychosocial oncology practice and research. Recently, the Cancer Experience Measurement Framework was proposed as a useful heuristic to capture patient and family cancer-related experiences in terms of personal and interactional processes (e.g., with healthcare). Herein, people’s distinct preferences for cancer information are used to depict how specific constructs embedded within the framework can best capture complexity and guide more tailored supportive approaches.

**Methodology or Methods**

A large survey (N = 2142) among people diagnosed with cancer within the last 6 months was conducted across 3 university-affiliated cancer centers in Montreal, QC. Participants completed the Ambulatory Oncology Patient Satisfaction Survey (AOPSS) and a brief cancer information-seeking preferences measure.

**Impact on practice or results**

Whereas 60.3% (n = 1292) reported wanting to actively seek information about their cancer; a surprisingly high percentage (i.e., 39.7%; n = 850) did not. Men reported avoiding cancer information to a greater extent than women, χ^2^ (4, N = 2108) = 12.00, *p* = 0.02. Preferences for seeking (or avoiding) cancer-related information were associated with six cancer care satisfaction domains, with intense seekers reporting significantly lower care satisfaction.

**Discussion or Conclusions**

Given the diversity of people’s cancer experiences and preferences, efforts should be made to efficiently capture these through explicit frameworks, concepts and psychometrically sound measures.

**30**

### 14.3. Feasibility of a Patient-Led Breast Cancer Registry: Amplifying Women’s Voices of the Lived Experience of Disease and Psychosocial Impact over Time Using Patient-Reported Outcomes: The PROgress Tracker Registry

Doris Howell ^1^, Shania Leduc, Amanda Gibson ^2^, Michelle Lianne Dean ^2^, Kimberly Carson ^3^ and Omar Khan ^4,5^.

^1^  Princess Margaret Cancer Research Institute, Toronto, Canada.
^2^  University of Calgary, Calgary, Canada.
^3^  Breast Cancer Canada, Sarnia, Canada.
^4^  Cumming School of Medicine, Calgary, Canada.
^5^  Annie Charbonneau Cancer Institute, Calgary, Canada.

**Background/rationale or objectives/purpose**

Breast cancer significantly impacts women’s psychosocial, physical health and quality of life. Directed by Breast Cancer Canada, a registered non-profit patient-led organization and managed by the University of Calgary’s POET Program, PROgress Tracker uses a novel, peer-to-peer support model for recruitment, engagement, retention and amplifying the voices of those with lived experience using Patient-Reported Outcome Measures (PROMs). Our objective is to share preliminary data on feasibility, participants and psychosocial impact.

**Methodology or Methods**

PROgress Tracker, a longitudinal cohort with 10-year enrollment goal of 50,000 Canadians with Stage 0-IV breast cancer captures disease information and extends a series of validated PROMs (PROMIS, PRO-CTCAE, BREAST-Q, FACT-B, PHQ-9, GAD, ISI, COST-FACIT and ESAS-r CA) via a digital platform every 3 months for up to 10 years with dynamic customization.

**Impact on practice or results**

To date, 162 participants from all Canadian geographic regions including remote/rural areas have shared demographic and clinical data including stage, genetic/molecular tumor markers and treatment type(s) and completed baseline PROMs with minimal missing data (<5%); 3-month follow-up surveys show a retention rate of 57%. Participants are young (median age: 54 yrs), Caucasian (95%) and currently working (57%). Of them, 46% reported financial stress (99% response rate) and 63% reported ongoing symptoms. In addition, 6% were BRCA+, 19% triple-negative, 7% experienced recurrence, 3% currently Stage IV, 75% received systemic and/or radiation therapy and 38% received targeted therapy.

**Discussion or Conclusions**

PROMs data can promote the understanding of the lived experience of breast cancer to inform precision/personalized psychosocial care. While early feasibility is evident for a PROgress tracker, further outreach and recruitment initiatives are needed for wide-scale reach.

**32**

### 14.4. Psychosocial Factors with a Focus on Hope in Rare Cancers: Using Mixed Methods to Capture Patients and Families’ Experiences with Biliary Tract Cancers

Samar Attieh ^1^, Christine Lafontaine ^1^, Leonard Angka ^1^, Melinda Bachini ^2^, Rebecca Auer ^1^ and Carmen G. Loiselle ^3,4^.

^1^  Cancer Research Program, Ottawa Hospital Research Institute, Ottawa, Canada.
^2^  Cholangiocarcinoma Foundation, Riverton, USA.
^3^  Department of Oncology and Ingram School of Nursing, Faculty of Medicine and Health Sciences, McGill University, Montreal, Canada.
^4^  Segal Cancer Center, Jewish General Hospital, Montreal, Canada.

**Background/rationale or objectives/purpose**

Biliary Tract Cancers (BTC) including cholangiocarcinoma and gallbladder cancers are characterized by their rarity, late diagnosis, resistance to traditional treatment, poor prognosis, psychosocial distress and hopelessness. Affected Canadians often face challenges accessing both standard healthcare services and promising novel therapeutics through clinical trials. Personal and contextual factors are known to play a significant role in shaping patients’ and families’ experiences and outcomes. The newly formed Canadian Cholangiocarcinoma Collaborative (C3) involves patients, informal caregivers, oncologists, researchers and trainees who work together to offer timely support and facilitate access to care and research. This includes guidance from a Research Navigator, clinical trial matching, access to molecular testing, participation in national BTC multidisciplinary rounds and other clinical research opportunities. Central to C3 and using mixed methods, this study seeks to (1) quantify how personal and contextual factors (e.g., hope, loneliness/social isolation and cancer information-seeking preferences) evolve among affected participants and (2) explore, qualitatively, their experiences with BTC and C3 initiatives.

**Methodology or Methods**

Thirty participants will complete e-measures at baseline, two weeks following the first C3 informational session with the Research Navigator and at 3 months. Five-to-seven dyads of patients and informal caregivers will take part in focus groups to explore their experiences further.

**Impact on practice or results**

Findings provide insights into personal and contextual resources that affect psychosocial adjustment to BTC.

**Discussion or Conclusions**

Findings can help inform future supportive care strategies, their implementation and research programs that foster hope among individuals affected by BTC.

**44**

### 14.5. Co-Designing a National Psychosocial Oncology Advocacy Agenda: Seeking Input from Multiple Stakeholders Including Canadian Cancer Organizations

Samar Attieh ^1^, Sevtap Savas ^2^, Kimberley Thibodeau ^3^ and Carmen G. Loiselle ^4^.

^1^  Department of Experimental Medicine, Faculty of Medicine and Health Sciences, McGill University, Montreal, Canada.
^2^  Division of Biomedical Sciences, Faculty of Medicine, Memorial University, St John’s, Canada.
^3^  McGill University Health Center, Montreal, Canada.
^4^  Department of Oncology and Ingram School of Nursing, Faculty of Medicine and Health Sciences, McGill University, Montreal, Canada.

**Background/rationale or objectives/purpose**

The Canadian Association of Psychosocial Oncology (CAPO) is in the process of bringing together diverse stakeholders, including healthcare professionals, researchers, patient representatives and advocacy organizations, to develop a national advocacy agenda centered around psychosocial oncology (PSO) priorities.

**Methodology or Methods**

Initially, an extensive search (i.e., Medline Ovid, social media and grey literature) and informal feedback followed by a brief CAPO survey served to identify Canadian organizations with a focus on advocating for PSO. A wider-scope survey, co-developed by various stakeholders including patient representatives, is underway to identify the key advocacy priorities, challenges, gaps, successful advocacy strategies and future advocacy directions of the identified organizations.

**Impact on practice or results**

The initial survey of 73 organizations identified 54 of these advocating for PSO. The most prevalent advocacy activities (n = 47; 87%) included raising public awareness about cancer-supportive needs and promoting initiatives that support person-centered psychosocial care for patients and caregivers. The second survey will provide in-depth data on PSO priorities and key action items.

**Discussion or Conclusions**

As we increasingly commit to co-designing cancer care, getting feedback from national organizations will ensure that we develop a comprehensive, inclusive and responsive PSO advocacy agenda.

**73**

### 14.6. So to Speak, So to Think, So to Feel: The Relationship between Metaphor and Coping with Cancer

Ben Stevenson ^1^, Karen Fergus ^1,2^, Iana Ianakieva ^1^ and Lucas Norton ^1^.

^1^  York University, Toronto, Canada.
^2^  Sunnybrook Hospital, Toronto, Canada.

**Background/rationale or objectives/purpose**

While metaphor has been identified as a fundamental part of everyday language and thought (Lakoff and Johnson, 1980), the role of metaphor in coping with cancer is not well understood. The current study investigated the relationship between metaphor and coping by examining participant responses to a Narrative Care Interview (NCI) designed to support patients during the post-treatment juncture. For the present analysis, we were interested in how patients’ implicit use of metaphor in their recounting of their illness related to coping with their diagnosis, treatment and recovery.

**Methodology or Methods**

The analysis was based on a subsample of 20 NCI participants. Participants were sampled purposively to attain heterogeneity of demographics (sex, age and ethnicity) and cancer type until saturation of themes was achieved. NCI transcripts were analyzed employing the ‘contemporary theory of metaphor’ (CTM) (Lakoff, 2004) to identify implicit metaphorical references to cancer and/or coping. The CTM holds that conceptual metaphors like CANCER IS A BATTLE are indicated by linguistic metaphors such as “I am a fighter” or “cancer won’t defeat me” and so on. We tracked all relevant instances of linguistic metaphors from interview transcripts and determined their underlying conceptual metaphors through an iterative and collaborative coding process.

**Impact on practice or results**

Our analysis yielded a rich account of how the implicit metaphoric framing of one’s illness plays a critical role in how individuals make sense of and cope with cancer.

**Discussion or Conclusions**

The identification of implicit metaphor use is valuable in revealing the unconscious dimensions of personal suffering and coping, evident in the everyday poetics of personal cancer narratives.

**81**

### 14.7. Patient Engagement in Cancer Research Community of Practice—Reducing Silos in Patient Engagement Practice

Heather Roy ^1,2,3^, Don Wood ^4,5,6^, Jacqueline Galica ^7,8^, Suzanne Bays ^4,9^, Justin Noble ^10,11^, Meera Rayar ^12,13,14^, Judit Takacs ^15,16^, Sevtap Savas ^17,18^ and Judy Needham ^19,20,21^.

^1^  Caregiver Parent, Calgary, Canada.
^2^  University of Calgary, Calgary, Canada.
^3^  Community of Practice Member, Calgary, Canada.
^4^  Caregiver Partner, Canadian Cancer Society, Toronto, Canada.
^5^  Canadian Cancer Clinical Trials Network, Toronto, Canada.
^6^  Community of Practice Steering Committee Member, Calgary, Canada.
^7^  Queen’s University, Kingston, Canada.
^8^  Community of Practice Member, Kingston, Canada.
^9^  Community of Practice Member, Montreal, Canada.
^10^  Ontario Institute for Cancer Research, Toronto, Canada.
^11^  Community of Practice Steering Committee Member, Toronto, Canada.
^12^  University of British Columbia, Vancouver, Canada.
^13^  BC Children’s Hospital, Vancouver, Canada.
^14^  Community of Practice Steering Committee Member, Vancouver, Canada.
^15^  Canadian Cancer Society, Toronto, Canada.
^16^  Community of Practice Secretariat, Toronto, Canada.
^17^  Memorial University of Newfoundland, St. John’s, Canada.
^18^  Community of Practice Steering Committee Member, St. John’s, Canada.
^19^  Chair, Patient Rep Committee, Canadian Cancer Trials Group, Kingston, Canada.
^20^  Breast International Group, Brussels, Belgium.
^21^  Community of Practice Steering Committee Member and Co-Chair, Kelowna, Canada.

**Background/rationale or objectives/purpose**

Patient engagement (PE) is a crucial part of meaningful cancer research, but has been siloed across Canada. Recognizing many PE initiatives exist, the Community of Practice (CoP)—Patient Engagement in Cancer Research was developed to bridge silos by building connections, developing and harnessing resources and facilitating collaborations.

Objectives: To describe the development, operation and evaluation of the CoP for patient-engaged cancer research.

**Methodology or Methods**

CoP documents were reviewed and descriptively summarized. Documents included group communications (i.e., emails), Terms of Reference, meeting agendas and minutes and results of a member survey.

**Impact on practice or results**

Initiated in 2022 by eight individuals including persons with lived experience, administrators and researchers, by December 2023 the CoP included 69 members from 45 organizations and 7 provinces representing research institutions, trial centers, sponsors, patient advocacy groups, industry and government. CoP development included the establishment of a secretariat for support (currently provided by Canadian Cancer Society) and a Steering Committee that developed Terms of Reference and has been responsible for issuing an initial call for membership and logistics including the number, timing and content of meetings, suggesting speakers and topics, implementing evaluations and determining future directions. A member survey after one year of CoP activity indicated positive experiences and a tangible value, including 21 new connections, cross-organizational collaborations and resource and strategy developments.

**Discussion or Conclusions**

The CoP is dedicated to reducing silos and growing involvement in PE in cancer research across Canada. Growth continues, both in membership and scope, as new avenues for connection and collaboration are explored. The CoP welcomes anyone with interest to join.

**88**

### 14.8. Influence of Patient and Caregiver Characteristics on Perspectives about Value Assessment Frameworks Used to Guide Oncology Treatment Decisions

Jenise Finlay ^1,2^, Stuart Peacock ^3^, Nicole Look Hong ^4,5^, Danielle Rodin ^6,7^, Kelvin Chan ^4,5^, Winson Cheung ^1^ and Colleen Cuthbert ^1^.

^1^  University of Calgary, Calgary, Canada.
^2^  University of Victoria, Victoria, Canada.
^3^  Simon Fraser University, Vancouver, Canada.
^4^  Sunnybrook Odette Cancer Center, Toronto, Canada.
^5^  University of Toronto, Toronto, Canada.
^6^  Princess Margaret Cancer Center, Toronto, Canada.
^7^  University of Toronto, Toronto.

**Background/rationale or objectives/purpose**

Patient demographics, life circumstances and past experiences influence information needs and preferences in treatment decisions. Value assessment frameworks quantify the treatment value by comparing costs to clinical outcomes (e.g., toxicity, survival, clinical benefit and symptom palliation). We explored patients’ and family caregivers’ perspectives on these frameworks, finding they prefer authentic and personalized communication, their values changed throughout the trajectory and existing frameworks lack the depth required for informed treatment decisions. This study examined how demographic characteristics influence these perspectives.

**Methodology or Methods**

Guided by interpretive description methodology, we conducted semi-structured interviews with patients and caregivers from Alberta, Ontario and British Columbia between November 2021 and August 2023. NVivo was used to organize data and coding was carried out to explore demographic variables in relation to themes.

**Impact on practice or results**

There were n= 60 participants including n = 37 females and n = 45 patients. Perspectives varied based on gender, age, role and cancer status. Caregivers emphasized survival and patients emphasized quality of life. Views on treatment outcomes varied by stage and duration of treatment. Females were more often concerned about intimacy, body image and identity and emphasized the importance of compassionate communication and adequate information. Positive communication experiences were more common among men and participants aged 60–79. Patients valued autonomy and feeling heard more frequently than caregivers.

**Discussion or Conclusions**

The findings highlight the diverse perspectives of patients and caregivers regarding cancer treatment decision making. These differences underscore the need for personalized approaches in treatment planning that consider individual values while recognizing the influence of demographics and family caregiver values in the decision-making process.

**96**

### 14.9. Empowering Neuro-Oncology Patients as Partners in Exercise Oncology Programming

Julia T. Daun ^1^, Megan Dow ^2^, Nola Stuckert ^2^, Tana Dhruva ^1^, Mannat Bansal ^1^, Lauren C. Capozzi ^1,3^, Meghan H. McDonough ^1^, Gloria Roldan Urgoiti ^4^, Jacob C. Easaw ^5^, Margaret L. McNeely ^6,7^, George J. Francis ^8,9^, Tanya Williamson ^1^ and S. Nicole Culos-Reed ^1,9,10^.

^1^  Faculty of Kinesiology, University of Calgary, Calgary, Canada.
^2^  Patient Partner, Calgary, Canada.
^3^  Department of Clinical Neurosciences, Cumming School of Medicine, Calgary, Canada.
^4^  Department of Medical Oncology, Alberta Health Services, Calgary, Canada.
^5^  Department of Medical Oncology, Cross Cancer Institute, Edmonton, Canada.
^6^  Department of Physical Therapy, University of Alberta, Edmonton, Canada.
^7^  Department of Oncology, Cancer Care Alberta, Edmonton, Canada.
^8^  Department of Clinical Neurosciences, Cumming School of Medicine, University of Calgary, Calgary, Canada.
^9^  Department of Oncology, Cumming School of Medicine, University of Calgary, Calgary, Canada.
^10^  Department of Psychosocial Resources, Tom Baker Cancer Center, Alberta Health Services, Calgary, Canada.

**Background/rationale or objectives/purpose**

Neuro-oncology patients remain an underserved patient population with limited access to exercise oncology resources. To support patient wellness and build systematic access to exercise oncology programming in neuro-oncology care, the Alberta Cancer Exercise Neuro-Oncology (ACE-Neuro) study was created. To ensure the development and delivery of a program that meets the unique needs of neuro-oncology patients, a patient-oriented research approach was employed across the study process.

**Methodology or Methods**

ACE-Neuro delivered a 12-week exercise program in Calgary and Edmonton. Across the build (i.e., development of program), implementation (i.e., delivery of program) and dissemination (i.e., translation of results) phases of ACE-Neuro, patients were engaged as partners. In phase one, patients were consulted on their needs for an exercise program. In phase two, patients and their families were invited to complete an interview to share their experiences participating in a tailored program. Finally, patients are included as co-authors for manuscripts and presentations as findings are analyzed and disseminated to both academic and lay audiences.

**Impact on practice or results**

A total of 43 patients have been engaged across the research process to date. Three patients were consulted during the build phase, twenty-eight patients and nine caregivers participated in delivery phase interviews and three patients supported dissemination via engagement in knowledge translation activities.

**Discussion or Conclusions**

ACE-Neuro is empowering patients and their families with opportunities for input to their supportive care. This process has ensured that ACE-Neuro is centered around patient-identified priorities and will thus support enhanced care and hope for future patients.

**159**

### 14.10. Two-Step Screening for Depression and Anxiety in Patients with Cancer: A Retrospective Validation Study Using Real-World Clinical Data

Bryan Gascon ^1^, Joel Elman ^2^, Alyssa Macedo ^3^, Yvonne Leung ^4,5^, Gary Rodin ^3,4,6^ and Madeline Li ^3,4,6^.

^1^  MD/PhD program, Temerty Faculty of Medicine, University of Toronto, Toronto, Canada.
^2^  Department of Supportive Care, Princess Margaret Cancer Center, University Health Network, Toronto, Canada.
^3^  Department of Supportive Care, Princess Margaret Cancer Center, University Health Network, Toronto, Canada.
^4^  Department of Psychiatry, Temerty Faculty of Medicine, University of Toronto, Toronto, Canada.
^5^  College of Professional Studies, Northeastern University, Boston, USA.
^6^  Institute of Medical Science, Temerty Faculty of Medicine, University of Toronto, Toronto, Canada.

**Background/rationale or objectives/purpose**

Screening for distress in cancer centers is recommended, but uptake is low. To improve the acceptability of screening programs, reducing clinical burden and improving distress detection are critical. The purpose of this study was to validate the performance of the two-step screening algorithm used in the Distress Assessment and Response Tool (DART) for identifying patients with anxiety and depression.

**Methodology or Methods**

This retrospective validation study consisted of patients at the Princess Margaret Cancer Center (PM) who completed the DART, which included the Edmonton Symptom Assessment System depression (ESAS-D) and anxiety (ESAS-A) items, the Patient Health Questionnaire (PHQ-9) and the Generalized Anxiety Disorder (GAD-7) questionnaire. We evaluated the performance of a two-step screening approach, which modeled ESAS-D followed by PHQ-9 and ESAS-A followed by GAD-7 for predicting depression and anxiety disorders, respectively. Comprehensive psychiatric assessment was used as the gold standard reference. 

**Impact on practice or results**

A total of 172 patients with cancer were included in this study; 39/172 (23%) were diagnosed with an anxiety disorder and 59/172 (34%) were diagnosed with depression. The sequential administration of ESAS-D followed by PHQ-9 significantly improved the performance of predicting depression compared to ESAS-D as a standalone test (ESAS-D C-index = 0.695 vs. ESAS-D + PHQ-9 = 0.728, *p* = 0.003). The sequential administration of GAD-7 after ESAS-A did not improve the predictability of an anxiety diagnosis beyond the performance of ESAS-A as a single test (ESAS-A C-index = 0.545 vs. ESAS-A + GAD-7 = 0.567, *p* = 0.22).

**Discussion or Conclusions**

The present study is among the first to demonstrate that a two-step screening algorithm for depression may improve depression screening in cancer using real-world data. Further research on optimal screening approaches for anxiety in cancer is warranted. The present study is among the first to demonstrate that a two-step screening algorithm for depression may improve depression screening in cancer using real-world data. Further research on optimal screening approaches for anxiety in cancer is warranted. The present study is among the first to demonstrate that a two-step screening algorithm for depression may improve depression screening in cancer using real-world data. Further research on optimal screening approaches for anxiety in cancer is warranted. The present study is among the first to demonstrate that a two-step screening algorithm for depression may improve depression screening in cancer using real-world data. Further research on optimal screening approaches for anxiety in cancer is warranted.

## 15. Final Category: O. Primary, Secondary and Tertiary Cancer Prevention

**156**

### 15.1. Head and Neck Cancer Awareness in University Students

Violet D’Souza ^1,2,3^, Jacob Pete ^1^ and Yuqi Wang ^1^.

^1^  Faculty of Dentistry, Dalhousie University, Halifax, Canada.
^2^  Department of Surgery, Faculty of Medicine, Dalhousie University, Halifax, Canada.
^3^  Department of Community Health and Epidemiology, Dalhousie University, Halifax, Canada.

**Background/rationale or objectives/purpose**

Head and neck cancers (HNC) are the seventh most common cancers worldwide and the ninth most frequent cause of death. Tobacco use, consuming large quantities of alcohol and human papillomavirus (HPV) infections are significant risk factors for HNCs and, thus, are preventable. The aim of this study was to investigate the university students’ knowledge and awareness of the HNC and HPV vaccines.

**Methodology or Methods**

This cross-sectional study was conducted with Dalhousie University students. The data were collected anonymously using a web-based survey tool to explore the students’ awareness of HNCs, HPV-related HNCs, vaccine knowledge, HPV vaccination status and willingness to get immunized against HPV. The data were analyzed using descriptive statistics, mean and standard deviations for continuous variables and the chi-square test for categorical variables.

**Impact on practice or results**

A total of 223 completed the survey. The mean age of the respondents was 23.4 years. Most (71%) were females, 66% were from the health science discipline (66%) and 50% were in Bachelor’s programs. Eighty-eight percent knew about HNCs, only 18% learned about them through their healthcare providers and 29% did not know that they were preventable. Though 66% had heard about HPV vaccines, only 43% had received them. Among the unvaccinated, 42% were willing to get vaccinated against HPV.

**Discussion or Conclusions**

Healthcare providers, specifically physicians and dentists, can screen their patients’ risk for HNCs, inform their patients about HNCs and their prevention to create HNC awareness and advocate screening and HPV immunization to reduce the HPV-related HNC burden.

**157**

### 15.2. Periodontal Disease and Its Association with Colorectal Cancers—A Systematic Review

Violet D’Souza ^1,2,3^ and Jasmine Mann ^1^.

^1^  Faculty of Dentistry, Dalhousie University, Halifax, Canada.
^2^  Department of Surgery, Faculty of Medicine, Dalhousie University, Halifax, Canada.
^3^  Department of Community Health and Epidemiology, Dalhousie University, Halifax, Canada.

**Background/rationale or objectives/purpose**

Periodontal disease (PD), the second most common oral disease, is associated with colorectal cancers (CRCs). CRC is the 3rd most common cancer affecting both men and women. The study aimed to examine the relationship between PD and CRC.

**Methodology or Methods**

This systematic review was conducted by systematically searching the electronic databases PubMed and Embase and manually searching for relevant scientific articles published on the topic. Original studies that investigated the causal relationship between PD and CRC were included if they were population-based, published in English before December 2022, moderate-to-high quality and had at least 50 individuals with CRC. Two reviewers independently screened studies while resolving the disagreement with consensus. Data were extracted using predetermined data extraction forms and a quality assessment was performed.

**Impact on practice or results**

Of the identified 4011 reports, 12 met the inclusion criteria and were included in the synthesis. Of them, seven were prospective cohort studies, four were retrospective cohort studies and one was a case-control study. The studies were of a moderate-to-high quality. In six studies, participants were at least 40 years or older. Three studies reported PD as a significant risk for CRC. One study examined the impact of PD management and reported that those who were treated for PD had a lower risk for CRC. The remaining studies had inconclusive findings.

**Discussion or Conclusions**

Our results suggest that periodontitis may be a risk factor for colorectal cancers, which needs to be confirmed. Also, the prevention and management of PD may reduce the burden of CRC.

## 16. Final Category: P. Sociodemographic, Culture and Sex/Gender Issues in Cancer

**22**

### 16.1. The Journey of Being Non-Binary and Diagnosed with Breast Cancer: A Case Study

Sara Cho and Claire Temple-Oberle.
University of Calgary, Calgary, Canada.

**Background/rationale or objectives/purpose**

A breast cancer diagnosis provides many psychosocial challenges and stresses to individuals and their loved ones. This stress can be further compounded when the diagnosis is typically associated with one gender (female gender) that is incongruent with one’s gender identity (e.g., non-binary). Unfortunately, there is a paucity of research on gender-diverse individuals’ experiences of breast cancer treatment.

The objective is to better understand how gender identity for a non-binary individual influences and plays a role in the breast cancer journey.

**Methodology or Methods**

Qualitative case study methodology was employed to explore the breast cancer journey of a non-binary individual. In-depth semi-structured interview will be conducted. The interview, blogposts and artwork from the individual were analyzed using thematic analysis.

**Impact on practice or results**

The case is a white, non-binary (assigned female at birth) individual (pronouns they/them) in their early 30s diagnosed with stage III invasive ductal carcinoma. This individual had undergone a bilateral mastectomy, is undergoing chemotherapy, radiation and hormone therapy.

A preliminary analysis of blogposts and artwork highlights their grapple with the psychosocial and physical toll cancer treatment has taken. Gender-specific subject matter includes conflicted emotions over unwillingly receiving a gender-affirming surgery (e.g., mastectomy) and hair as part of gender expression. The work also highlights negative and positive healthcare interactions.

**Discussion or Conclusions**

Providers should be aware of the complex relationship some non-binary individuals may have with their breasts and subsequent treatment for breast cancer. More work is needed to educate providers to provide safe and affirming spaces for gender-diverse individuals undergoing cancer treatment.

**142**

### 16.2. Retrospective Chart Review Documentation of Sexual Health Counseling in Adolescent and Young Adult Patients with a Vagina Receiving Radiotherapy

Kaviya Devaraja ^1,2^, Abha A. Gupta ^3,4^ and Jennifer Croke ^5^.

^1^  Institute of Medical Science, University of Toronto, Toronto, Canada.
^2^  Adolescent and Young Adult Program, Department of Supportive Care, Princess Margaret Cancer Center, Toronto, Canada.
^3^  Adolescent and Young Adult Program, Department of Supportive Care, Princess Margaret Cancer Center, University of Toronto, Toronto, Canada.
^4^  Division of Medical Oncology and Hematology, Princess Margaret Cancer Center, University of Toronto, Toronto, Canada.
^5^  Radiation Medicine Program, Princess Margaret Cancer Center, University Health Network, Toronto, Toronto, Canada.

**Background/rationale or objectives/purpose**

Radiotherapy (RT) plays an important role in pelvic malignancies for patients with a vagina. However, associated sexual and vaginal side-effects can impact a patient’s mental health, relationships and quality of life. Sexual health counseling before, during and after therapy are crucial for adolescent and young adult (AYA) (aged 15–39) patients with a vagina, given the unique developmental, emotional and educational aspects of sexual dysfunction during this critical life stage. Our study aims to identify gaps in sexual health counseling for AYAs with a vagina receiving RT to enhance the development of targeted supportive care tools.

**Methodology or Methods**

This retrospective study will examine records of AYAs with a vagina receiving RT between January 2017 and December 2023, at the Princess Margaret Cancer Center. Patient identification will be through the Mosaic database and data extraction will include socio-demographics, diagnosis, treatment details, sexual health counseling, fertility and family planning, sexual dysfunction and overall well-being.

**Impact on practice or results**

Descriptive statistics will summarize quantitative data, comparing data across different groups.

**Discussion or Conclusions**

Our findings will contribute to improving sexual health for AYAs affected by RT by identifying and addressing existing gaps in sexual health counseling. The findings will guide the development of resources, programs and guidelines to deliver comprehensive and effective sexual health counseling, prioritizing interventions tailored to the unique needs of this demographic.

## 17. Final Category: Q. Survivorship

**10**

### 17.1. Next Steps after Breast Cancer Workshop

Luana Flores Pereira and Andrea Carolina Benites.
Princess Margaret Cancer Center, Toronto, Canada.

**Background/rationale or objectives/purpose**

Breast cancer is the most commonly diagnosed cancer in the world, with an estimated 80% 10-year survival. Many breast cancer survivors experience long-term side-effects from treatment and express unmet psychosocial and informational needs, impacting quality of life and confidence navigating survivorship.

**Methodology or Methods**

A virtual educational program was developed to provide information and support to individuals after breast cancer treatment. Presentations delivered monthly by patient partner, clinical nurse specialist, occupational therapist, kinesiologist, dietician and social work included common experiences after completing active treatment and supports and resources available. Participants completed a pre-survey with an option to list their questions and a post-survey for program evaluation.

**Impact on practice or results**

Data collected from June to December 2023 showed 86% of the participants completed the pre-survey and 53% completed post-survey. The level of knowledge in all topics covered (side-effects from treatment, exercising, nutrition, coping, returning to activities and resources available) increased from “fair” to “good”, participants felt questions were very well answered, confidence in navigating survivorship increased from “fair” to “good” and overall satisfaction with workshops was rated as “very good”. The participants also shared feeling supported, validated, informed, grateful and felt, overall, less isolated.

**Discussion or Conclusions**

The virtual workshop is a feasible and effective way to provide orientation and support to breast cancer survivors. The importance of a support group to address psychosocial needs was also identified. Thus, providing more opportunities for peer connection during survivorship could help individuals coping with fear of recurrence and navigating the next steps after treatment.

**14**

### 17.2. Perspectives of Cancer Survivors: Qualitative Analyses of Concerns, Positive Experiences and Suggestions for Improvement through Low-Income and Rural-Living Lens

Irene Nicoll ^1^, Gina Lockwood ^2^ and Margaret Fitch ^3^.

^1^  Independent Health Services Consultant, Toronto, Canada.
^2^  Independent Biostatistician Consultant, Toronto, Canada.
^3^  Bloomberg Faculty of Nursing, University of Toronto, Toronto, Canada.

**Background/rationale or Objectives/purpose**

Cancer survivor numbers in Canada have reached 1.5 million and are expected to grow. It is important to understand cancer survivors’ perspectives regarding the challenges they face after treatment. Various factors can create barriers to accessing assistance; limited income and living rurally may be significant ones. Our goal was to explore major challenges, positive experiences and suggestions for improvement in survivorship care through the lens of a low income and through the lens of living rurally.

**Methodology or Methods**

This presentation describes two secondary qualitative analyses of data from a publicly available databank (Cancer Survivor Transitions Study) regarding experiences of Canadian cancer survivors 1–3 years following treatment. A content analysis was performed for each sample, one with low incomes and one living rurally, utilizing written comments to open-ended questions.

**Impact on practice or results**

A total of 1708 respondents indicated having a low income (<CAD 25,000/year) and 4646 respondents reported living rurally. The most frequently identified challenges identified in each sample were a reduced physical capacity and limits resulting from treatment effects. Positive experiences in both samples emphasized support from family/friends and engaging in self-care activities. Similar suggestions for improvements included needs for better communication and information about self-care and side-effect management. Low-income individuals wanted more programs to assist with finances while rural-dwelling individuals emphasized the availability of programs locally.

**Discussion or Conclusions**

The relationships between the income and management of survivors’ physical, emotional and practical concerns require consideration. A low income or rural living can be barriers to accessing help during survivorship. Risk assessments for potential barriers ought to be conducted prior to transition to survivorship.

**17**

### 17.3. Relationships among Income and Physical, Emotional and Practical Concerns and Help-Seeking in Older Adult Survivors of Cancer (65+ Years)

Fay Strohschein ^1^, Lorelei Newton ^2^, Irene Nicoll ^3^, Gina Lockwood ^4^, Carmen G. Loiselle ^5^, Christopher J. Longo ^6^, Lauren M. Fitch ^7^ and Margaret I. Fitch ^8^.

^1^  University of Calgary, Edmonton, Canada.
^2^  University of Victoria, Victoria, Canada.
^3^  Independent Health Services Consultant, Toronto, Canada.
^4^  Independent Biostatistician Consultant, Toronto, Canada.
^5^  McGill University, Toronto, Canada.
^6^  McMaster University, Hamilton, Canada.
^7^  Moncton City Hospital, Riverview, Canada.
^8^  University of Toronto, Toronto, Canada.

**Background/rationale or objectives/purpose**

Cancer survivors can be left with physical and emotional challenges following treatment which may have profound effects on instrumental activities of daily living. Obtaining assistance for these issues can be problematic for various reasons, including financial. The situation may be especially difficult for older individuals with fixed incomes. This study sought to understand relationships among older survivors’ income and experiences finding help for concerns post-treatment.

**Methodology or Methods**

A Canada-wide survey explored experiences of cancer survivors 1–3 years after treatment. Questionnaires were distributed to a random sample through ten provincial cancer registries. A trend analysis describes associations among income and older cancer survivors’ physical, emotional and practical concerns and help-seeking.

**Impact on practice or results**

The overall survey response rate was 33% (N = 12,929), of which 7975 (62%) respondents were 65+ years. Of these, 5891 (74%) indicated their annual income. Prostate (31%), colorectal (23%) and breast (22%) cancers accounted for 76% of the respondents. Over 90% experienced physical changes, 80% experienced emotional changes and 77% experienced practical changes following treatment. Up to half of those indicated major concerns about the changes. Significant trends across income categories were evident for eight physical symptoms, five emotional concerns and three practical concerns. Survivors with a low annual income (<CAD 25,000) reported higher levels of concern about multiple changes and greater difficulties finding help than those with higher incomes.

**Discussion or Conclusions**

Older cancer survivors can experience various unmet needs post-treatment and difficulty finding help. Those with a lower income were most severely affected. An early assessment of financial risk among older survivors is recommended.

**20**

### 17.4. Living Well with Impairment after Cancer: An Arts-Based Exploration of Women’s Experiences

Sandra Houle and Roanne Thomas.
University of Ottawa, Ottawa, Canada.

**Background/rationale or objectives/purpose**

A pervasive assumption in health care is that one must be absent of impairment to live a good life after cancer treatment. However, women have reported positive adaptation and overall well-being, despite long-term physical and psychosocial impacts. Nonetheless, little research has examined how women living with cancer experience the apparent dichotomy between living well and living with impairment. Consequently, there have been few opportunities for women to share their lived experiences of this phenomenon. Using arts-based methods, our research team explored how women reimagined a good life despite experiencing impairment.

**Methodology or Methods**

Sample and setting: Ten women who experienced cancer participated in the study. They engaged in a series of virtual, mixed-media, arts-based workshops facilitated by a community artist and arts-based researcher.

Procedures: The workshops were video-recorded and art pieces were photographed. We also conducted individual, audio-recorded interviews pre- and post-workshops. All recordings were transcribed verbatim and analyzed along with the photographs, using interactive and reflexive processes (NVivo).

**Impact on practice or results**

Our data analysis revealed three inter-related themes related to the women envisioning a good life as a process of positive adaptation and resilience in the face of impairment. These include (1) acknowledging the “good with the bad”, (2) being open to change and (3) choosing growth and acceptance.

**Discussion or Conclusions**

Our study helps to expand the current knowledge on living a good life beyond cancer. Our findings reinforce the need for more expansive approaches to cancer research and care to support individuals in navigating the realities of living well despite impairment.

**23**

### 17.5. Early Intervention Necessary for Breast Cancer Survivors: An Analysis of the 3-Year Trajectory of Insomnia, Fatigue and Cognitive Impairment

Rachel Lee ^1,2^, Krista Greeley ^1,2^, Joshua Rash ^1^, Melanie Seal ^1^, Joy McCarthy ^3^ and Sheila Garland ^1,2^.

^1^  Memorial University of Newfoundland, St. John’s, Canada.
^2^  Beatrice Hunter Cancer Research Institute, Halifax, Canada.
^3^  Eastern Health, St. John’s, Canada.

**Background/rationale or objectives/purpose**

Breast cancer is the most common form of cancer among Canadian women. Survivorship challenges include fatigue, sleep disturbance and cognitive impairment. This study examined (1) the symptom trajectory from diagnosis to three years, (2) whether symptom changes in the first four months were associated with prolonged difficulties after three years and (3) which factors were associated with deterioration in symptoms during the first four months.

**Methodology or Methods**

This prospective observational cohort study examined 53 women (*M_age_* = 58.6, 96.2% White, 67.9% Stage I) with newly diagnosed breast cancer over three years. Women completed assessments within 30 days of starting treatment and four months and three years after diagnosis. Three-way repeated-measures ANOVAs evaluated symptom trajectories. A repeated-measures mediation analysis was performed to determine if change from pre-treatment to four months accounted for the change from pre-treatment to three years. A series of between-subject ANOVAs were used to determine what variables significantly differed by the deterioration status.

**Impact on practice or results**

The perceived cognitive impairment and fatigue increased linearly from diagnosis to three years. A change in fatigue in the first four months fully accounted for its change over three years. Insomnia severity and sleep quality deteriorated from diagnosis to four months, but returned to pre-treatment levels at three years. Those whose fatigue and cognitive ability deteriorated during the first four months were younger.

**Discussion or Conclusions**

Efforts to identify those who are at risk of experiencing fatigue, sleep disturbance and cognitive impairment, monitor patients early after receiving a diagnosis and provide targeted interventions may prevent long-term deterioration and improve well-being.

**27**

### 17.6. Educational Outcomes in Survivors of Acute Lymphoblastic Leukemia and Solid Tumor Controls

Mehak Stokoe ^1^, Jenny Duong ^1^, Kaelyn McDonald-Wirasinghe ^1^, Caitlin Forbes ^1^, Heide Adham ^1^, David Nordstokke ^1^, Gabrielle Wilcox ^1^ and Fiona Schulte ^1,2^.

^1^  University of Calgary, Calgary, Canada.
^2^  Alberta Children’s Hospital, Calgary, Canada.

**Background/rationale or objectives/purpose**

This study is used to examine differences on the measures of reading, spelling and math computation between survivors of pediatric acute lymphoblastic leukemia (ALL) and non-central nervous system solid-tumor (non-CNS ST) controls. This will enable us to understand treatment-related differences that may impact these areas of achievement.

**Methodology or Methods**

Data collection is ongoing. As part of a larger study, participants attend a one-time appointment at the Alberta Children’s Hospital. Reading, spelling and math computation are assessed using the Wide Range Achievement Test—Fifth Edition (WRAT-5).

**Impact on practice or results**

Currently, 12 survivors have participated (n = 6 survivors of ALL; mean age = 12.5 (SD = 2.7) years; n = 6 non-CNS ST controls; mean age = 13.2 (SD = 2.6) years). The highest completed grade level ranged from 2–10 for survivors of ALL and 4–11 for non-CNS ST controls. The mean scores on WRAT-5 reading, spelling and math computation fell within the Average range for survivors of ALL: 99.3 (SD = 9.9), 91.2 (SD = 8.0) and 92.7 (SD = 14.3), respectively. However, for non-CNS ST controls, the scores fell within the Average range for reading (97.2 [SD = 13.4]) and Low-Average range for spelling (87.3 [SD = 21.9]) and math computation (87.7 [SD = 14.5]). An independent sample t-test revealed no statistically significant differences between groups across all measures.

**Discussion or Conclusions**

Functional implications include providing more specific instruction about spelling (e.g., sounds of words and types of errors) and math computation (e.g., correct operations). Future research should focus on interventions targeting these areas so students do not fall behind peers and to understand why non-CNS ST survivors may be achieving lower in spelling and math.

**28**

### 17.7. Barriers and Enablers to Attending Long-Term Follow-Up Care among Survivors of Pediatric Cancer in Canada

Holly Wright ^1^, Sharon Hou ^1,2^, Brianna Henry ^1^, Rachelle Drummond ^1^, Kyle Mendonca ^3^ and Fiona Schulte ^1,3^.

^1^  Department of Oncology, Cumming School of Medicine, University of Calgary, Calgary, Canada.
^2^  Department of Psychology, BC Children’s Hospital, Vancouver, Canada.
^3^  Alberta Children’s Hospital, Calgary, Canada.

**Background/rationale or objectives/purpose**

Survivors of pediatric cancer are at risk for post-treatment late effects and require diagnosis-specific long-term follow-up (LTFU) care. Yet, less than 50% of survivors attend LTFU care. This study aimed to identify barriers and enablers of attending LTFU care perceived by survivors of pediatric cancer and healthcare providers (HCPs).

**Methodology or Methods**

Canadian survivors (*n* = 108, M_age_ = 28.3 years) and HCPs (*n* = 20, M_age_ = 48.3 years) were recruited via LTFU clinics and social media to complete surveys assessing barriers and enablers to LTFU care that were summarized using descriptive statistics. Participants were invited to participate in a follow-up, including survivor focus groups (*n* = 22) or HCP semi-structured interviews (*n* = 7) that were analyzed using reflexive thematic analysis.

**Impact on practice or results**

Survivors reported work (27.8%, *n* = 30), location (25.0%, *n* = 27) and finances (21.3%, *n* = 23) as barriers. HCPs reported work (85.0%, *n* = 17), transportation (85.0%, *n* = 17) and location (80.0%, *n* = 16) as barriers. Focus groups/interview themes were generally consistent with these findings. Additional themes that emerged included the survivors’ lived experience and the healthcare system capacity as barriers. Survivors reported family support (66.7%, *n* = 72), healthcare team support (56.5%, *n* = 61) and friend support (40.7%, *n* = 44) as enablers. HCPs reported family support (100.0%, n = 20), healthcare team support (95.0%, *n* = 19), employer support (80.0%, *n* = 16) and healthcare team familiarity (80.0%, *n* = 16) as enablers. Focus groups/interview themes were generally consistent with these findings. Additional themes that emerged included resources and self-advocacy as enablers.

**Discussion or Conclusions**

The results identify barriers and enablers to attending LTFU care. The findings can guide interventions to improve LTFU care engagement, with the potential to better manage and decrease the late effect burden.

**31**

### 17.8. Cancer-Related Dyadic Efficacy for Navigating the Post-Treatment Transition: A Qualitative Research Protocol for Examining Couples’ Goal-Directed Actions and Efficacy Appraisals

Danielle Brosseau ^1^, José Domene ^2^, Karen Fergus ^3,4^ and Jill Turner ^5^.

^1^  King’s University, Edmonton, Canada.
^2^  University of Calgary, Calgary, Canada.
^3^  York University, Toronto, Canada.
^4^  Sunnybrook Odette Cancer Center, Toronto, Canada.
^5^  Cross Cancer Institute, Edmonton, Canada.

**Background/rationale or objectives/purpose**

Annually, more than 150,000 Canadians transition into cancer survivorship. This post-treatment transition commonly involves physical, psychological and practical challenges for survivors and their partners. Dyadic illness management theory outlines an interaction between dyadic appraisals and dyadic management behaviors that influence each member’s health and the health of the dyad. This study will examine couples’ dyadic efficacy, a confidence appraisal in the couple’s joint abilities, as it relates to the goal-directed actions couples complete during the transition to survivorship.

**Methodology or Methods**

Eligible couples (*N* = 30) will be composed of individuals who are 18+ years of age with one person who completed primary treatment for cancer within the past year. Purposive sampling will enhance sample diversity regarding gender, cancer type and race. The Action-Project Method of qualitative research will be used to identify couples’ actions and goals as they adjust to life after primary treatment. Data will be collected in an initial interview, 6-month monitoring period and final interview. Analysis will focus on descriptions and observations of intentions, actions, efficacy and goals.

**Impact on practice or results**

This study will yield detailed descriptions of the health-related goals that couples pursue individually and jointly when adjusting to life after cancer. These actions, together with the couples’ dyadic efficacy appraisals, can inform the focus of personalized survivorship care plans.

**Discussion or Conclusions**

Investigating what couples do to manage their holistic health during the transition to survivorship will enhance the scaffolding of support practices to augment couples’ existing efforts. The researchers invite feedback on methodological decisions (i.e., inclusion criteria) and suggestions for dissemination formats to reach professionals involved in survivorship care planning.

**46**

### 17.9. Improving Cancer-Related Fatigue through Cognitive Behavioral Therapy for Insomnia: A Secondary Analysis of a Randomized Controlled Trial

Krista Greeley ^1^, Joshua Tulk ^2^, Rachel Lee ^1^, Joshua Rash ^1^ and Sheila Garland ^1^.

^1^  Memorial University of Newfoundland, St. John’s, Canada.
^2^  University of Calgary, Calgary, Canada.

**Background/rationale or objectives/purpose**

Cancer-related fatigue (CRF) is one of the most common negative side-effects experienced after a cancer diagnosis and is often comorbid with insomnia, cognitive impairment (CI) and mood disturbances. Cognitive behavioral therapy for insomnia (CBT-I) has shown efficacy in improving CRF and cognitive impairment in cancer survivors. The current study evaluated whether CBT-I resulted in a significant improvement in CRF, after statistically adjusting for a change in insomnia, CI and mood disturbances.

**Methodology or Methods**

Cancer survivors from Atlantic Canada with insomnia and cognitive impairments completed CBT-I as part of a randomized controlled trial. Participants were randomized to the CBT-I or waitlist control. Measures of CRF (Multidimensional Fatigue Symptom Inventory—Short Form; MFSI), insomnia (Insomnia Severity Index), CI (Functional Assessment of Cancer Therapy—Cognitive version) and mood disturbances (Hospital Depression and Anxiety Scale) were administered at pre-, mid- and post-treatment. Clinically significant improvement in the CRF was defined as a reduction of 10.79 points.

**Impact on practice or results**

The sample consisted of 132 cancer survivors (78% female, M_age_ = 60.12 years). The most common cancer type was breast (45%). A mixed-effects model adjusting for insomnia, CI and mood disturbances showed a significant group-by-time interaction post-treatment, *p* < 0.001. The group who completed CBT-I experienced a 20.6-point reduction in CRF compared to a 3.7-point reduction in the waitlist control.

**Discussion or Conclusions**

CBT-I resulted in a significant improvement in CRF relative to waitlist control and these effects were not accounted for by a change in insomnia, CI and mood disturbances. CBT-I is a robust intervention with efficacy for improving fatigue among cancer survivors with insomnia.

**47**

### 17.10. How Social Support Helps Integrate Cancer into One’s Identity: An Interpretative Phenomenological Analysis of Experiences of Long-Term AYA Leukemia Survivors

Camille Bourdeau ^1,2^, Andy Charbonneau ^1^, Carole Provost ^3^, Émélie Rondeau ^4^, Caroline Laverdière ^3,5^, Roxane de la Sablonnière ^1^ and Serge Sultan ^1,4,5^.

^1^  Department of Psychology, Université de Montréal, Montreal, Canada.
^2^  Research Center, CHU Sainte-Justine, Canada.
^3^  Hemato-Oncology Department, CHU Sainte-Justine, Montreal, Canada.
^4^  Research Center, CHU Sainte-Justine, Montreal, Canada.
^5^  Department of Pediatrics, Université de Montréal, Montreal, Canada.

**Background/rationale or objectives/purpose**

Beside challenges associated with survivorship, AYA cancer survivors face unique issues related to their psychosocial development. Integrating the cancer experience into one’s identity could promote positive long-term adjustment in this population. Social support is considered as key to this integration, but little is known about how this occurs. Survivors are experts to uncover potential mechanisms of the action of social support.

**Methodology or Methods**

Long-term pediatric leukemia survivors (n = 12, 10 women, age = 18–27 years) were recruited in the long-term follow-up clinic of CHU Sainte-Justine. We led in-depth individual narrative interviews (McAdams, 2007) and analyzed them within an Interpretative Phenomenological Analysis framework (Smith et al., 2022). A post hoc description of well-being (WHO-5), social support (SSQ-6) and health status (15D) was also available.

**Impact on practice or results**

The verbal material highlights the meaning of the cancer experience and how social support may reinforce or undermine each experiential facet. Four Group Experiential Themes emerged from the participants’ discourse: protecting others and the loss of childhood, being different and seeking normality through the approval of others, characteristics of support contributing to the participants’ identity integration and the role of others in growing after cancer.

**Discussion or Conclusions**

Focusing on the subjective experience of participants provides a deep understanding of how social support functions promote adjustment. The social support mechanisms identified could help to optimize interventions directed to families and friends in aftercare. The present narrative study sheds light on the survivors’ perceptions of their own trajectory of how social relationships helped them integrate their cancer experience into their identity.

**54**

### 17.11. Post-Traumatic Reactions and Quality of Life after Pelvic Exenteration for Gynecologic Cancer

Kristine A. Donovan ^1^, Lora M.A. Thompson ^2^, Nele Loecher ^3^ and Alexandra Albizu-Jacob ^4^.

^1^  Mayo Clinic, Rochester, USA.
^2^  Moffitt Cancer Center, Tampa, USA.
^3^  St. Jude Children’s Research Hospital, Memphis, USA.
^4^  University of South Florida, Tampa, USA.

**Background/rationale or objectives/purpose**

Pelvic exenteration (PE) is a rare, radical, life-altering surgery to treat recurrent gynecologic cancer. The current study sought to examine women’s post-traumatic reactions and quality of life (QOL) after PE.

**Methodology or Methods**

Participants completed Impact of Event Scale-Revised (IES-R), Post-Traumatic Growth Inventory (PTGI) and European Organization for Research and Treatment of Cancer 30-item core Quality of Life Questionnaire (EORTC QLQ-C30).

**Impact on practice or results**

Twenty-one women (mean age = 57.5 years; mean time since surgery = 3.8 years) reported a mean IES-R score of 21.2 ± 19.4; 30% had scores suggesting significant traumatic stress symptoms. The mean PTGI score was 68.2 ± 17.8. Correlations between the IES-R and PTGI scores were moderate and negative: more stress was associated with less growth. The global health status on the EORTC-QLQ-C30 was about average (57.5 ± 21.9). The IES-R and PTGI total scores, respectively, were negatively and positively associated with the global health status. The function scale scores ranged from 45.2 ± 33.8 for social function to 73.02 ± 24.4 for cognitive function. More stress was associated with lower functioning in all domains except social functioning. More growth was associated with greater emotional and cognitive function. Symptom scales ranged from 11.1 ± 14.3 for nausea/vomiting to 52.4 ± 37.4 for financial difficulties. More stress was associated with more fatigue, pain, dyspnea, insomnia and appetite loss. More growth was associated with less fatigue and insomnia.

**Discussion or Conclusions**

Both traumatic stress and growth are present after PE and likely contribute to QOL. Findings have clinical implications for survivorship care. Psychosocial interventions to address traumatic stress symptoms and to improve coping and adaptation should be recommended and available to patients to improve long-term QOL.

**68**

### 17.12. Understanding the Contribution of Attitude, Perceived Control, Subjective Norms and Intention to Physical Activity and Nutrition Behaviors in Pediatric Oncology

Ariane Levesque ^1,2^, Daniel Curnier ^1,2^, Valérie Marcil ^1,2^, Maxime Caru ^3^, Émélie Rondeau ^2^, Caroline Meloche ^2^, Isabelle Bouchard ^2^, Caroline Laverdière ^1,2^, Daniel Sinnett ^1,2^ and Serge Sultan ^1,2^.

^1^  Université de Montreal, Montreal, Canada.
^2^  Sainte-Justine University Health Center, Montreal, Canada.
^3^  Penn State College of Medicine, Hershey, USA.

**Background/rationale or objectives/purpose**

To promote healthy lifestyles in pediatric cancer aftercare and survivorship, it is necessary to understand how and why families with children diagnosed with cancer engage in health behaviors. This study aimed to (1) explore adherence to contrasted physical activity and nutrition lifestyle scenarios and (2) examine the contribution of attitude, perceived control, subjective norms and intention to parent-reported child behaviors in these scenarios.

**Methodology or Methods**

Four questionnaires measuring variables from the Theory of Planned Behavior were completed by parents to assess attitude, perceived control, subjective norms, intention and behavior. A MANOVA was conducted to investigate aim 1. Bootstrap multiple linear regressions and the PROCESS macro for mediation analyses were used to investigate aim 2.

**Impact on practice or results**

The MANOVA yielded a main effect of the behavior domain (F(3, 4828.66) = 6.467, *p* < 0.001, ηp2 = 0.004) and of the healthy/unhealthy scenario (F(3, 152.86) = 76.495, *p* < 0.001, ηp2 = 0.600). We found that attitude, perceived control and subjective norms contributed to behavior. We found that intention was a significant partial mediator in the relationships between attitude/perceived control/subjective norms and behavior.

**Discussion or Conclusions**

Variables from the Theory of Planned Behavior contribute to physical activity and nutrition behaviors. This study showed that attitude should be targeted for both physical activity and nutrition behaviors (healthy and unhealthy), that perceived control only contributes to adherence to healthy behaviors (in physical activity and nutrition) and that subjective norms only contribute to nutrition behavior. Intention should also be targeted as a result of its significant role as a mediator.

**79**

### 17.13. Exploring Expectancy and Perceptions of Credibility among Cancer Survivors with Cognitive Complaints Who Received Treatment for Insomnia

Brianna George ^1^, Josua Tulk ^1^, Josée Savard ^2,3^, Joshua Rash ^1^, Christopher Quinn-Nilas ^1^ and Sheila Garland ^1,4^.

^1^  Department of Psychology, Faculty of Science, Memorial University, St. John’s, NL, Canada.
^2^  School of Psychology, Université Laval, Quebec, QC, Canada.
^3^  CHU de Québec-Université Laval Research Center, Quebec, QC, Canada.
^4^  Discipline of Oncology, Faculty of Science, Memorial University, St. John’s, NL, Canada.

**Background/rationale or objectives/purpose**

Cognitive Behavioral Therapy for Insomnia (CBT-I) is an evidence-based intervention that effectively improves insomnia and perceived cognitive impairment (PCI) in cancer survivors. This secondary analysis of a randomized controlled trial (RCT) of virtually delivered CBT-I to improve PCI examined post-treatment perceptions of insomnia and the PCI outcome.

**Methodology or Methods**

Virtual interviews were conducted using a subsample of cancer survivors from Atlantic Canada, following their participation in the larger RCT of CBT-I. All participants met DSM-5 criteria for insomnia disorder and had PCI. A semi-structured interview guide explored the impact of CBT-I and factors associated with credibility and expectancy beliefs. Findings were synthesized using a reflexive thematic approach.

**Impact on practice or results**

Among the 21 participants, (M_age_ = 65, SD = 8.3; 95% female), 71% reported improvements in their sleep and cognition after CBT-I. The themes related to improvement in sleep and cognition were: (1) consistence and persistence, (2) a relationship with the therapist and (3) convenience of the virtual treatment delivery format. Of the participants who held positive pre-treatment beliefs, 52% commented on a logical connection between cognition and sleep and were open-minded about the treatment. Still, many had doubts and were skeptical, owing in part to the lack of understanding of CBT-I.

**Discussion or Conclusions**

Pre-treatment beliefs shape the perception of, and engagement in, treatment. Clinicians can enhance the effectiveness of CBT-I among cancer survivors by (1) providing psychoeducation to promote an understanding of sleep, (2) setting appropriate pre-treatment beliefs that foster optimism and mitigate skepticism, (3) promote consistent engagement with treatment and (4) foster positive therapeutic relationships.

**87**

### 17.14. Utilizing Patient-Reported Outcomes Measurement Information System (PROMIS) Measures to Screen for Psychosocial Distress in Children Attending a Long-Term Survivor Clinic

Caitlin Forbes ^1^, Mehak Stokoe ^2^, Jenny Duong ^1^, Jennifer Giles ^3^, Kathleen Reynolds ^1,3^ and Fiona Schulte ^1,3^.

^1^  Cumming School of Medicine, University of Calgary, Calgary, Canada.
^2^  Werklund School of Education, University of Calgary, Calgary, Canada.
^3^  Hematology, Oncology, and Transplant Program, Alberta Children’s Hospital, Calgary, Canada.

**Background/rationale or objectives/purpose**

Survivors of childhood cancer (SCC) are at risk of experiencing psychological distress. Regular screening is recommended by the International Guideline Harmonization Group for Late Effects of Childhood Cancer to ensure timely access to appropriate services. Screening must be reliable but avoid becoming burdensome for patients and clinicians. This study describes the use of parent-proxy PROMIS measures as part of a clinical screening tool.

**Methodology or Methods**

Parents of children attending regular follow-up appointments complete an online health questionnaire. Anxiety and depression are assessed through yes/no questions (e.g., Is your child having problems with anxiety?) and short-form PROMIS measures. PROMIS measures are automatically scored (T-scores with mean 50, SD = 10) and color-coded to flag areas of concern for the clinician (i.e., scores > 65 are red for severe). Parents of 96 patients (average age = 11.9 years (SD = 2.63); age at diagnosis = 2.7 years (SD = 2.17)) consented to including their child’s most recent questionnaire in this study.

**Impact on practice or results**

Measures of anxiety (r(91) = 0.424, *p* < 0.001) and depression (r(92) = 0.487, *p* < 0.001) were moderately correlated. Nine (9.4%) parents reported their child as “feeling frequently sad or depressed”, yet 14 (14.6%) scored in the moderate/severe range on the PROMIS measure. Clinicians reported that the color flagging informed their clinical plan and focused conversations.

**Discussion or Conclusions**

PROMIS measures identified psychosocial distress in more patients than the single-item questions. These short and free measures were able to facilitate discussion between patients, parents and the healthcare team to ensure that SCC are provided with access to appropriate resources.

**93**

### 17.15. A Quality Improvement Project to Optimize Access to Psychosocial Care for Cancer Survivors with Fear of Recurrence

Luana Flores Pereira, Patricia Nguyen, Aronela Benea and Carol Townsley.
Women’s College Hospital, Toronto, Canada.

**Background/rationale or objectives/purpose**

The prevalence of moderate-to-high levels of fear of cancer recurrence (FCR) may vary from 22% to 87%. The After-Cancer Treatment Transition (ACTT) clinic in the Women’s College Hospital (Toronto) provides follow-up care to cancer survivors, but in a sample of patients seen, only 1.5% were referred to a social worker for psychosocial support. Our goal was to evaluate the use of the single-question screening tool for FCR among cancer survivors and its impact on access to psychosocial care.

**Methodology or Methods**

Between July and October 2022, patients completed the screening tool ranging from 1 (no fear) to 5 (fearful all the time) during their clinic visit. Those who endorsed moderately severe (4)-to-high levels (5) of fear of cancer recurrence were offered a referral to social work.

**Impact on practice or results**

In total, 330 (41.9%) patients completed the screening tool for FCR. Most patients were female (96%), the average age was 60 years and most were diagnosed with breast cancer (90%). Among the screened patients, 118 (35.7%) indicated moderate and 37 (11%) a moderately severe-to-high level of FCR. All 37 were offered a social work referral and 22 (59.5%) accepted the referral. In comparison, referrals increased from 1.5% to 6.7% after screening implementation.

**Discussion or Conclusions**

Implementing FCR screening in an ACTT clinic was feasible with the use of the single-question screening tool, which optimized the access to psychosocial support by identifying patients in need and prompting referrals to appropriate services. The screening rate may potentially increase with an automated process, whereby the screening tool is smoothly integrated into the flow of clinical care.

**95**

### 17.16. Program Evaluation Plan for a Hybrid Psychoeducational/Cognitive Behavior and Process Group Intervention for New Cancer Survivors

Kim Crosby ^1,2^, Michelle McLean ^2^, Nicola Michaud ^1^, Ceinwen Cumming ^1,2^ and Treena Hinse ^1^.

^1^  Alberta Health Services, Edmonton, Canada.
^2^  University of Alberta, Edmonton, Canada.

**Background/rationale or objectives/purpose**

This presentation describes the evaluation plan to assess the short-term impact of a group intervention tailored for cancer patients transitioning into survivorship. The Mental Wellness After-Treatment group is an 8-week 90 min virtual and in-person hybrid psychoeducational/cognitive behavior and process group intervention tailored for cancer patients recently or near to discharge from cancer treatment in Alberta. The group content addresses a number of common concerns identified by cancer survivors.

**Methodology or Methods**

Planned outcome evaluation to assess the short-term effects of group intervention on health-related quality of life (HRQOL), coping response and fear of cancer recurrence during transition to survivorship. Group participants will be invited to complete an optional and anonymous survey, pre- and post-group. The survey will collect demographic and cancer-related information (i.e., type of cancer, types of treatments), self-report questionnaires for HRQOL, adaptive/maladaptive coping responses and subjective fear of cancer recurrence. In the post-group survey, additional open-ended questions will be added to elicit feedback from participants regarding group experiences.

**Impact on practice or results**

This evaluation will answer the following questions: To what extent does the group intervention impact subjective health-related quality of life? To what extent does group intervention increase the use of adaptive coping strategies and reduce the use of maladaptive coping responses? Finally, to what extent does the intervention impact subjective fear of cancer recurrence.

**Discussion or Conclusions**

The results of this evaluation will serve to inform future group planning and tailoring the intervention according to patient needs and feedback.

**97**

### 17.17. “Now What?” Development of a Hybrid Psychoeducational/Cognitive Behavior and Process Group Intervention for Post-Cancer Transition

Kim Crosby ^1,2^, Nicola Michaud ^1^, Michelle McLean ^2^ and Treena Hinse ^1^.

^1^  Alberta Health Services, Edmonton, Canada.
^2^  University of Alberta, Edmonton, Canada.

**Background/rationale or objectives/purpose**

As survival rates improve across cancers, more patients are transitioning into survivorship. Commonly reported unmet needs for cancer survivors include physical, emotional, relational, personal and practical concerns (Shakeel, Tung, Rahal and Finley, 2020). This presentation will describe the development of a group intervention tailored towards the psychosocial and informational needs of cancer survivors post-treatment.

**Methodology or Methods**

An 8-week hybrid psychoeducational/cognitive behavior and process group intervention was designed for cancer patients to facilitate transition post-treatment. First, a literature review was conducted of prominent biopsychosocial concerns of post-treatment individuals. This informed group topics, which included physical and cognitive consequences of cancer and treatment, returning to work, emotional impact of cancer experience, fear of cancer recurrence, social support and relationships, meaning-making and post-traumatic growth and navigating health care post-cancer. The group includes cognitive behavioral, psychoeducational and process-oriented interventions.

**Impact on practice or results**

The group intervention was launched in 2023 and is presently in its fourth cohort at the Cross Cancer Institute in Edmonton, Alberta. It has been presented in both virtual and in-person contexts facilitated by two registered psychologists. The first three cohorts consisted of patients in the Edmonton/North Zones and the fourth has been expanded to include patients in Calgary and the South Zone.

**Discussion or Conclusions**

The implementation of this group intervention post-cancer treatment has the potential to streamline transition to survivorship, reduce impact of psychosocial distress and improve quality of life for patients.

**98**

### 17.18. Conceptual Frameworks Utilized to Explore Factors Associated with Long-Term Follow-Up Care for Survivors of Childhood Cancer: A Scoping Review

Rachelle Drummond and Fiona Schulte.
University of Calgary, Calgary, Canada.

**Background/rationale or objectives/purpose**

Adherence to long-term follow-up care (LTFU) for survivors of childhood cancer (SCC) has been shown to improve the long-term wellbeing of survivors through the early detection of secondary cancers and monitoring of late-effects. However, less than 50% of survivors adhere to recommended LTFU care. Several factors have been studied that may be related to adherence, but little research has considered factors in a conceptual framework. This study aimed to review existing frameworks used to explore adherence to LTFU for SCC.

**Methodology or Methods**

Three databases (MEDLINE, EMBASE and PubMed) were searched. Included frameworks must (1) be applied in a population of SCC and (2) measure (quantitatively) or capture (qualitatively) adherence. SCC include those diagnosed from 0 to 18 years of age who have completed all cancer treatments.

**Impact on practice or results**

One conceptual framework was identified: Oeffinger’s (2003) theoretical model of barriers and enablers for adherence to LTFU care among adult SCC. Such limited findings highlighted a significant gap in conceptualizing the unique challenges and experiences that SCC face in adhering to LTFU care. To address this research gap, we propose a six-stage hierarchical framework that outlines specific skills, behaviors and beliefs that a SCC must acquire or overcome in order to adhere to LTFU care.

**Discussion or Conclusions**

One SCC-specific conceptual model may not encompass the complex interactions between the unique experiences and factors that may impact a survivor’s ability to adhere to LTFU. The conceptualization of a framework that considers the unique experiences and needs of SCC can help guide evidence-based research aimed at improving adherence to LTFU care for SCC.

**101**

### 17.19. Investigating Social Information Processing, Social Adjustment and the Impact of Radiation in Survivors of Pediatric Brain Tumors

Courtney Tromburg ^1,2^, Hailey Zwicker ^2^, Mehak Stokoe ^2^, Caitlin Forbes ^2^, Taryn Fay-McClymont ^3^, Gregory Guilcher ^2^, Lucie Lafay-Cousin ^2^, Douglas Strother ^2^, Keith Yeates ^2^, Kevin Krull ^4^ and Fiona Schulte ^2.^

^1^  University of Alberta, Edmonton, Canada.
^2^  University of Calgary, Calgary, Canada.
^3^  University of British Columbia Okanagan, Kelowna, Canada.
^4^  St. Jude Children’s Research Hospital, Memphis, USA.

**Background/rationale or objectives/purpose**

Research understanding potential social adjustment and social information processing (SIP) deficits in survivors of pediatric brain tumors (SPBTs) is limited. This study investigated the differences in SIP between SPBT who did or did not receive cranial radiation therapy (CRT) and healthy controls. In addition, we explored the relationship between components of SIP (theory of mind [ToM], affect recognition, executive function and attention) and social adjustment.

**Methodology or Methods**

SPBT were recruited from a pediatric hospital. Eligible participants were 8–18 years old, >2 years post-treatment or 5 years post-diagnosis and not enrolled in full-time special education. Healthy controls were recruited through a healthy child cohort.

Caregivers completed parent-proxy reports of executive function, attention and social adjustment. SPBT completed assessments of affect recognition and ToM.

**Impact on practice or results**

In total, 32 SPBT (53.1% female, mean age = 13.4, received radiation = 14) and 35 controls (48.6% female, mean age = 11.09) participated. SPBT performed significantly worse than the controls on affect recognition, *t*(39.6) = −2.84, *p* = 0.007, executive function, *t*(56.4) = 2.91, *p* = 0.005 and social adjustment *t*(65) = −4.18, *p* < 0.001. No significant group differences in ToM were identified. SPBT who completed CRT had poorer scores on affect recognition compared to those who did not, *t*(30) = 2.19, *p* = 0.037. Group membership, age, sex, affect recognition and attention together explained 34% of the total variance in social adjustment, *F*(5,51) = 6.69, *p* < 0.001.

**Discussion or Conclusions**

Affect recognition and attention are key components of SIP and appear to be compromised in SPBT, especially those who received CRT, compared to healthy peers. This study may influence future interventions in SPBT wellbeing.

**105**

### 17.20. Assessing Gender as a Moderator for Likelihood of Cannabis Use for Sleep in Colorectal Cancer Survivors

Chloe House, Rachel Lee, Jennifer Donnan, Nick Harris and Sheila Garland.
Memorial University of Newfoundland, St. John’s, Canada.

**Background/rationale or objectives/purpose**

Roughly one in four cancer survivors use cannabis as a sleep aid, despite a lack of empirically demonstrated efficacy. Individuals with colorectal cancer report a high degree of symptom burden. Research in the general population suggests that gender may affect the likelihood of use and consumption patterns. This study investigates whether gender influences the likelihood of cannabis use for sleep in a subset of colorectal cancer survivors.

**Methodology or Methods**

Adult Canadian cancer survivors were recruited via the Angus Reid Institute. Participants provided information on their sleep using the insomnia severity index and their cannabis use behaviors. Moderation analyses were performed to determine if gender influences the relationship between cancer type and methods of cannabis ingestion.

**Impact on practice or results**

Of the participants (*N* = 94), 41 men and 53 women (M_age_ = 66.4) with colorectal cancer participated. Of them, 23.2% of men (*n* = 10) and 20.7% of women (*n* = 11) diagnosed with colorectal cancer used cannabis as a sleep aid. Gender moderated the likelihood of using cannabis for sleep (β = 0.066, *p* = 0.021), indicating that men are more likely to use cannabis for sleep than women. Men were more likely to vaporize cannabis, *p* = 0.033, and use concentrated THC formulations, *p* = 0.036, than women. There was no gendered difference in the likelihood of ingesting cannabis by smoking, oils, capsules, edibles, topical applications and concentrated CBD.

**Discussion or Conclusions**

Men and women with colorectal cancer appear to use cannabis differently to help with sleep. Future research should explore the reasons for these differences.

**107**

### 17.21. Cannabis Use and Insomnia Severity among Cancer Survivors with and without Anxiety and Depression

Emily White ^1^, Rachel Lee ^1,2^, Payton Peach ^1^, Jennifer Donnan ^3^, Nick Harris ^1^ and Sheila Garland ^1,2,4^.

^1^  Department of Psychology, Faculty of Science, Memorial University of Newfoundland, St. John’s, Canada.
^2^  Beatrice Hunter Cancer Research Institute, Halifax, Canada.
^3^  School of Pharmacy, Memorial University of Newfoundland, St. John’s, Canada.
^4^  Discipline of Oncology, Faculty of Medicine, Memorial University, St. John’s, Canada.

**Background/rationale or objectives/purpose**

This study investigated differences in cannabis use and insomnia severity among cancer survivors with and without psychiatric conditions.

**Methodology or Methods**

Canadian cancer survivors completed an online survey that included questions about psychiatric diagnoses, cannabis use and the Insomnia Severity Index (ISI). Participants (*N* = 1464) were grouped by psychiatric diagnoses: only diagnosed with depression (*n* = 124), only anxiety (*n* = 92), both depression and anxiety (*n* = 91) or no psychiatric condition (*n* = 874). Participants with other psychiatric conditions (*n* = 176) or who did not disclose (*n* = 107) were excluded. Descriptive statistics and one-way ANOVAs were performed on ISI scores and cannabinoid content for each group.

**Impact on practice or results**

Of the participants, current cannabis use for sleep was reported by 27% (*n* = 34) with depression only, 28% (*n* = 26) with anxiety only, 41% (*n* = 37) with comorbid depression and anxiety and 18% *(n* = 155) with no psychiatric condition.

Among participants with comorbid depression and anxiety, the most common cannabinoid content consumed was balanced amounts of THC and CBD (*n* = 14, 38%). Cannabis containing mostly THC was most common for those with depression only (*n* = 11, 32%), anxiety only (*n* = 9, 36%) and no psychiatric condition (*n* = 63, 41%).

Participants with comorbid depression and anxiety that consumed cannabis with mostly THC (*n* = 9, 24%) had greater insomnia severity (*M* = 15.22) than those that consumed balanced THC and CBD (*M* = 8.21), *p* = 0.019, but not those that consumed mostly CBD (*n* = 8, 22%; *M* = 8.63), *p* = 0.076. There were no significant differences in the ISI scores based on the cannabinoid content for those with depression only, anxiety only or no psychiatric condition.

**Discussion or Conclusions**

Cancer survivors appear to be self-medicating with cannabis to manage mood and insomnia symptoms.

**112**

### 17.22. Evaluating the Use of a Video to Introduce Sexual Health Education to Female Adolescents and Young Adults with Cancer

Natalie Pitch ^1^, Rebecca Cote ^1^, Chana Korenblum ^1^, Jennifer Catsburg ^2^, Jonathan Avery ^3^ and Abha Gupta ^4^.

^1^  SickKids Hospital, Toronto, Canada.
^2^  Princess Margaret Cancer Center, Toronto, Canada.
^3^  Princess Margaret Cancer Center, Toronto, Canada.
^4^  SickKids, Toronto, Canada.

**Background/rationale or objectives/purpose**

Female adolescents and young adults (AYAs) aged 15–39 with cancer have life-long risks of developing sexual health-related challenges. However, there is a gap in the provision of sexual health-related care. The AYA Program at the Princess Margaret Cancer Center developed a patient education video that explains common sexual health challenges that can arise from cancer and its treatment. Our study aims to evaluate the effectiveness, understandability and perceived quality of the sexual health video with AYAs.

**Methodology or Methods**

A mixed-methods sequential (Qual-Quant) approach will be used. English-speaking females between the ages of 15 and 39 with a diagnosis of cancer will be recruited from a pediatric hospital in Toronto, Canada, and from across Canada through our collaborators. Before watching the video, demographic information will be collected and AYAs will complete a knowledge-check questionnaire. They will complete the same questionnaire after viewing the video, with additional items asking for their impressions of the content and delivery. *T*-tests will be used to analyze whether a significant difference exists between pre- and post-video knowledge. Bivariate analyses will be performed to determine if certain demographic variables predict knowledge change. Focus groups will explore the results of the survey.

**Impact on practice or results**

This poster will showcase preliminary findings from surveys. We predict that there will be a meaningful knowledge change between the pre- and post-video surveys.

**Discussion or Conclusions**

While it is difficult to predict the general impressions of the video, we will use these findings to guide focus groups to gain in-depth evaluations of the video from the perspective of AYAs.

**117**

### 17.23. The Ottawa Clinical Fear of Recurrence (OCFR) Measures for Clinical Fear of Recurrence: First Look into the Screener’s Psychometric Properties

Lauriane Giguère ^1^, Emma Kearns ^2^, Mutsaers Brittany ^1^, Cheryl Harris ^2^, Smith Ben ^3^, Gerald Humphris ^4^, Daniel Costa ^5^, Cary Kogan ^1^, Sebastien Simard ^6^ and Sophie Lebel ^1^.

^1^  School of Psychology, University of Ottawa, Ottawa, Canada.
^2^  The Ottawa Hospital Research Institute, Ottawa, Canada.
^3^  The Daffodil Center, The University of Sydney, Sydney, Australia.
^4^  School of Medicine, University of St Andrews, St Andrews, United Kingdom.
^5^  School of Psychology, The University of Sydney, Sydney, Australia.
^6^  Department of Health Sciences, Université du Québec à Chicoutimi (UQAC), Saguenay, Canada.

**Background/rationale or objectives/purpose**

The Ottawa Clinical Fear of Recurrence Screener (OCFR-S or FCR-1r) was created to add a one-item screener to the measures currently used to routinely assess psychosocial issues in cancer centers. The screener, rating fear of cancer recurrence (FCR) on a 10-point scale, was first piloted with a group of survivors who deemed the item wording and response scale acceptable.

**Methodology or Methods**

The screener was subsequently administered online to 300 mixed cancer survivors from The Ottawa Hospital who agreed to be contacted for research. The screener was compared to the Edmonton Symptoms Assessment System—Revised (ESAS-R)’s anxiety item and assessed for its incremental validity using hierarchical regressions in R.

**Impact on practice or results**

The data analysis is underway. The results will be presented.

**Discussion or Conclusions**

This study complements the preliminary validation of the screener providing more support for the screener’s validity. The screener will be an important addition to the toolbox of brief validated assessment instruments available to healthcare professionals to detect potential clinically significant FCR.

**121**

### 17.24. Advocating for the Right to Choose: Exploring the Needs, Challenges and Opportunities to Enhance Oncofertility Support for Adolescent and Young Adults with Cancer

Jonathan Avery ^1,2^, Lillian Rogers ^1^, Tiffany Hill ^1^, Param Gill ^1^, Ada Okonkwo ^1^, Jennifer Wolfe ^1^ and Cheryl Heykoop ^1^.

^1^  Royal Roads University, Victoria, Canada.
^2^  Princess Margaret Cancer Center, Toronto, Canada.

**Background/rationale or objectives/purpose**

Fertility preservation is an essential consideration for adolescent and young adults (AYAs) (individuals between 15 and 39 years of age) facing cancer treatment. Yet, many barriers exist that prevent AYAs from being informed about their options and navigating the process. Building from the principles of participatory action and patient-oriented research, we facilitated a co-development workshop with AYAs with cancer, to explore oncofertility needs, challenges and opportunities to support AYAs more effectively.

**Methodology or Methods**

We engaged in a 2 h co-development session over Zoom with 15 AYAs. The session focused on discussions exploring what AYAs need when navigating oncofertility, what healthcare providers need to know and what opportunities exist for improvement. Group discussions were audio-recorded and transcribed verbatim. A graphic recorder was also present to create graphic recordings of the session. Transcripts and graphic recordings were analyzed using the constant comparative method.

**Impact on practice or results**

The participants expressed a lack of agency to decide for themselves if they wanted to pursue fertility preservation. Advocating for the right to choose, needing honest and clear communication that is holistic and patient-centered and having universal access to oncofertility information, affordable preservation and mental health support emerged as key themes to support young adults more effectively.

**Discussion or Conclusions**

Fertility is a key piece of the AYA identity that transcends age, gender and culture and additional work is needed to explore the lived experience of under-represented groups. The next steps include using these results to explore fertility in more depth through a funded project with the Canadian Partnership Against Cancer.

**126**

### 17.25. Living with Lung Cancer: A Mixed-Methods Study on the Experience of Fear of Progression in Individuals with Lung Cancer Receiving Immunotherapy or Targeted Therapy

Alanna Chu ^1^, Cheryl Harris ^2^, Sara Moore ^3^, Veronika Huta ^1^, Christine McPherson ^1^, Kimberly McMillan ^1^, Rinat Nissim ^4^ and Sophie Lebel ^1^.

^1^  University of Ottawa, Ottawa, Canada.
^2^  The Ottawa Hospital, Canada.
^3^  The Ottawa Hospital, Ottawa, Canada.
^4^  University Health Network, Toronto, Canada.

**Background/rationale or objectives/purpose**

Lung cancer is the leading cause of cancer deaths in Canada. The recent development of immunotherapy (IO) and targeted therapy (TT) drugs offer a new hope for prolonging survival in patients with advanced lung cancer. However, patients’ responses to treatment are variable (ranging from months to multiple years) and treatment does not have a curative intent. Patients must cope with long periods of uncertainty and fears about progression and death, which is associated with significant distress. This study aims to update an existing model of Fear of Cancer Recurrence/Progression (FoP). Specifically, to assess the experience of and relationship between FoP, illness uncertainty, symptom burden, supportive care needs and death anxiety.

**Methodology or Methods**

A mixed methods study will be conducted. Approximately 150 patients with advanced lung cancer receiving IO or TT from The Ottawa Hospital will complete quantitative measures of fear of cancer progression, death anxiety, symptom burden, demographic and medical information, supportive care needs and uncertainty in illness. Qualitative interviews will be conducted in a subset of the participants to obtain an in-depth understanding of the survey responses.

**Impact on practice or results**

Recruitment is ongoing. Recruitment rates, progress and preliminary results will be presented.

**Discussion or Conclusions**

Lung cancer is the highest-incident cancer in Canada and the population of long-term survivors is rapidly growing owing to IO and TT. It is vital to understand FOP in this population in order to adapt existing FOP interventions to meet the needs of these individuals.

**127**

### 17.26. Construct Validity of the Ottawa Clinical Fear of Cancer Recurrence—Measure (OCFR-M) in a Large Cohort of Cancer Survivors

Suchithra Shenthil ^1^, Lauriane Giguère ^1^, Brittany Mutsaers ^1^, Cheryl Harris ^1,2^, Ben Smith ^3^, Gerald Humphris ^4^, Daniel Costa ^3^, Cary Kogan ^1^, Sebastien Simard ^5^ and Sophie Lebel ^1^.

^1^  University of Ottawa, Ottawa, Canada.
^2^  The Ottawa Hospital, Ottawa, Canada.
^3^  The University of Sydney, Sydney, Australia.
^4^  University of St Andrews, St Andrews, United Kingdom.
^5^  Université du Québec à Chicoutimi (UQAC), Saguenay, Canada.

**Background/rationale or objectives/purpose**

Fear of cancer recurrence (FCR) is a common challenge among cancer survivors that can be associated with adverse outcomes including psychological comorbidity, diminished quality of life and increased healthcare usage. Many healthcare providers lack sufficient training and tools to assess FCR effectively, leading to the underutilization of psychosocial support services and challenges in managing patients’ emotional and psychological needs. To address this gap, this study evaluates the convergent and discriminant validity of the newly developed self-report Ottawa Clinical Fear of Recurrence Measure (OCFR-M) in a sample of 300 cancer survivors.

**Methodology or Methods**

Approximately 300 cancer survivors from The Ottawa Hospital completed a questionnaire package that included the OCFR-M, the FCR Inventory—Short Form (FCRI-SF), the Cancer Worry Scale (CWS) and Patient-Reported Outcome Measures Information System (PROMIS) scales for depression, anxiety and life satisfaction (PROMIS-D-8a, PROMIS-A-8a and PROMIS-GLS-SF5a). Descriptive statistics characterized the sample and correlational analyses evaluated construct validity. The convergent validity between OCFR-M and established FCR measures (FCRI-SF and CWS) and discriminant validity between OCFR-M and related psychological constructs measured by PROMIS scales were calculated.

**Impact on practice or results**

Data collection is currently ongoing, with results forthcoming.

**Discussion or Conclusions**

This study proposes the OCFR-M as a brief tool to assess FCR in cancer survivors. As a promising standardized tool, the OCFR-M has the potential to quickly and accurately screen and identify patients requiring additional support.

**148**

### 17.27. The Role of Tattoos in Response to Breast Cancer: An Intersection of Art and Health

Jennifer Buckle and Sonya Corbin Dwyer.
Grenfell Campus, Memorial University, Corner Brook, Canada.

**Background/rationale or objectives/purpose**

The prevalence of tattoos has increased exponentially in recent years. With that increase has come a greater appreciation and understanding of the role tattoos may play for those who choose them, such as self-expression, memorialization, commemoration and social connection. While the motivations for obtaining a tattoo are becoming increasingly well-established, there is much less known about the role of tattooing in response to health conditions, particularly in response to breast cancer. The limited research in this area has highlighted the theme of using tattooing as a means of reclaiming control over the body after treatment for breast cancer, specifically with respect to mastectomy scars (Allen, 2017; Reid-de Jong, 2022). Given the limited research, the purpose of the present qualitative study was to explore the role of tattoos in response to breast cancer, in all their forms and placement on the body.

**Methodology or Methods**

Ten women with tattoos in response to their breast cancer diagnosis and treatment, ranging in age from 42–65 years (mean age of 54.30 years), volunteered to be interviewed and to have their tattoos photographed.

**Impact on practice or results**

Using hermeneutic phenomenology analysis, key themes emerged highlighting the important role these tattoos played in the psychological recovery and healing from breast cancer, with the imagery and symbolism in the tattoo photographs providing rich visual data to support and illustrate these significant themes.

**Discussion or Conclusions**

The results of this study offer a unique and compelling perspective on well-being in recovery, as the tattoos provided a meaningful representation of how these women processed their breast cancer experiences.

**155**

### 17.28. Oral Health-Related Quality of Life in Head and Neck Cancer Survivors—An Interim Analysis

Violet D’Souza ^1,2,3^, Shauna Hachey ^1,4^, Yuqi Wang ^5^ and Rebecca Affoo ^6^.

^1^  Dalhousie University, Halifax, Canada.
^2^  Department of Surgery, Faculty of Medicine, Dalhousie University, Halifax, Canada.
^3^  Department of Community Health and Epidemiology, Dalhousie University, Halifax, Canada.
^4^  School of Dental Hygiene, Faculty of Dentistry, Halifax, Canada.
^5^  Faculty of Dentistry, Halifax, Canada.
^6^  School of Communication Sciences and Disorders, Faculty of Health, Dalhousie University, Halifax, Canada.

**Background/rationale or objectives/purpose**

To assess the oral health-related quality of life (OHRQoL) of head and neck cancer survivors (HNCS) in Nova Scotia.

**Methodology or Methods**

This cross-sectional study was conducted in Halifax, Nova Scotia. Participants were HNCS who completed cancer treatment at least one year prior to participating in the study. The OHRQoL was assessed using the Oral Health Impact Profile 14 (OHIP-14), a 14-item questionnaire with responses ranging from ‘Never’ to ‘Very often’ and scores ranging from 0–56. A higher score indicates a worse OHRQoL. Also, participants’ oral health status and dental care needs were assessed through clinical examination. Informed consents were obtained from all participants.

**Impact on practice or results**

Twenty-two HNCS (17 male and 5 female) participated in the study. The mean age was 69 years, 82% had a dental visit within the past year and 77% had dental insurance. The mean number of missing teeth and decayed teeth requiring restoration and/or extraction were 10.09 and 1.09, respectively. The mean OHIP-14 score was 25.7, which represents 46% of the scale range, indicating a significantly poor quality of life. Fifty-nine percent of participants reported “Often” or “Very often” to at least one OHRQoL item. “Having trouble pronouncing words” and “Being uncomfortable to eat any foods” were the most reported impacts.

**Discussion or Conclusions**

Our study findings suggest that HNC survivors continue to experience significant challenges in their mouths that affect their day-to-day functioning. While these findings are important, they need to be confirmed. Furthermore, other factors contributing to the OHRQoL of HNCS need to be explored to enhance HNCS survivorship care planning.

## 18. Final Category: R. Other Value-Based and Person-Centered Cancer Care

**18**

### 18.1. Patients’ and Healthcare Professionals’ Awareness of Psychosocial Oncology Resources and Services in Nova Scotia: Challenges and Recommendations

Janice Howes, Marianne Arab, Leslie Hill, Jill Petrella and Devbani Raha.
NS Health, Halifax, Canada.

**Background/rationale or objectives/purpose**

Connecting cancer patients with Psychosocial Oncology (PSO) resources at the appropriate time can be challenging. During the fall of 2022, cancer patients and health care providers (HCPs) of the Nova Scotia Health Cancer Care Program (NSH CCP) were surveyed to better understand their awareness of and access to PSO patient resources.

**Methodology or Methods**

Four hundred and thirty-two (432) respondents completed the patient survey and 104 respondents completed the HCP survey.

**Impact on practice or results**

Patient survey results indicated that the majority of patients rated psychosocial services and information as a very important or an important part of their cancer care. Patient respondents had moderate-to-low awareness of specific psychosocial resources.

The HCP survey results indicated that HCP awareness of specific PSO resources ranged from 18% to 79%. A majority of HCPs reported that they never recommend specific PSO resources to patients, with the exception of printed information sheets.

We are now working to raise awareness and develop a communication plan for both HCPs and patients.

**Discussion or Conclusions**

The low awareness of cancer patients for specific PSO resources is mirrored by the low awareness by HCPs. Of patients who were unaware of PSO resources, many would have liked to have received information. Patients clearly value PSO resources and indicated that they want provision of services and resources throughout their cancer experience. We are addressing the frequency with which HCPs recommend PSO resources. We will discuss the use of a new electronic platform to improve both patient and HCP awareness of PSO resources.

**74**

### 18.2. Conceptualizing Cancer Patient Navigation as the First Step in a Supportive Care Pathway

Claire Link, Andrea DeIure, Kathryn Burrows, Se’era Anstruther and Linda Watson.
Alberta Health Services, Calgary, Canada.

**Background/rationale or objectives/purpose**

The incidence of cancer is rising and patient volumes are higher than ever. Each patient’s cancer journey will be different and recognizing this helps Cancer Care Alberta (CCA) provide person-centered care, understanding that the unique circumstances of each patient shape their experience and supportive care needs. The Cancer Patient Navigation (CPN) program within CCA was established in 2012 and provides tailored navigation support to Albertans. Recognizing the growing population of patients with distinct needs and circumstances, who may benefit from navigation support, CCA set out to conduct a robust evaluation of the CPN program in 2023.

**Methodology or Methods**

Interviews were completed with multiple stakeholders across the province including Supportive Care Services, clinical educators and managers and the navigators themselves. Supporting analyses were conducted using administrative data and patient experience data from CCA’s Ambulatory Oncology Patient Satisfaction Survey.

**Impact on practice or results**

Findings from the evaluation indicate that having navigation as the first step in a supportive care pathway is an enabler to providing ongoing supportive care throughout the patient’s cancer journey. With CPNs assessing patients’ needs at the intake and encouraging the adoption of a collaborative, multi-disciplinary, person-centered approach, navigation can help ensure patients are well-supported in all aspects of their care, beyond only the medical treatment of cancer.

**Discussion or Conclusions**

This session will discuss the importance of CPNs as step one in an effective supportive care intervention and how navigation can facilitate patient connections and referrals to other specialized supportive care services such as social work, psychology, rehabilitation/exercise, palliative care and the further medical management of symptoms if necessary.

**111**

### 18.3. Distress Screening with Patient-Reported Outcomes in Gynecologic Oncology Patients Undergoing Active Treatment: Common Concerns

Julien Courville ^1^, Krisen Jean Julien ^2^, Samara Perez ^3^, Sylvie Lambert ^4^ and Shuk On Annie Leung ^1^.

^1^  Division of Gynecologic Oncology, Department of Obstetrics and Gynecology, McGill University Health Center, Montreal, Canada.
^2^  St. Mary’s Research Center, CIUSSS de l’Ouest de l’Ile de Montréal, Montreal, Canada.
^3^  Department of Oncology, Faculty of Medicine and Health Sciences, McGill University, Montreal, Canada.
^4^  Ingram School of Nursing, McGill University, Montreal, Canada.

**Background/rationale or objectives/purpose**

Psychosocial distress is common among patients with gynecologic malignancies and has been associated with reduced adherence to treatment, morbidity and mortality. Canadian cancer programs have been mandated to implement distress screening to identify psychosocial distress in patients. This study aimed to explore some main causes of distress in gynecologic oncology patients who are undergoing active treatment.

**Methodology or Methods**

All consenting gynecologic oncology patients receiving anti-cancer treatment at the McGill University Health Center since September 2022 were included. The Edmonton Symptom Assessment System Revised and Distress Thermometer was administered at initial and follow-up visits. Descriptive statistics were subsequently performed.

**Impact on practice or results**

There were 12 patients. The most reported symptoms associated with any level of distress were well-being issues (80%) and tiredness (77%). Meanwhile, tiredness (25%) and sleep (14%) were the issues most reported and associated with severe distress. Nausea (10%) and diarrhea (12%) were the least reported symptoms. The main practical concerns among patients included body image (25%), travel/holidays (25%) and feeling like a burden to others (24%).

**Discussion or Conclusions**

The most bothersome symptoms included those associated with well-being and fatigue, while nausea and diarrhea were the least bothersome. This highlights the need to focus on sleep hygiene, light exercise and behavioral therapy. Meanwhile, the patients’ biggest concerns included body image, traveling and feeling like a burden to others and need to be included in supportive care services for this patient sub-group. These distress screening data allow the tailoring of appropriate psychosocial resources and the development of targeted interventions.

**118**

### 18.4. Improving Pediatric Radiation Patient Experience through the Usage of Video Distraction

Heather Roy ^1,2^, Dr Natalie Logie ^3,4,5^, Dr Douglas Holt ^6,7^ and Brian Miller ^8,9,10^.

^1^  University of Calgary—Masters of Social Work Student, Calgary, Canada.
^2^  Patient Parent, Calgary, Canada.
^3^  University of Calgary—Clinical Assistant Professor, Calgary, Canada.
^4^  Tom Baker Cancer Centre—Radiation Oncologist, Calgary, Canada.
^5^  FRCPC, DABR, Calgary, Canada.
^6^  Eastern Idaho Regional Medical Center—Radiation Oncologist, Idaho Falls, USA.
^7^  St. John’s Hospital—Radiation Oncologist, Jackson Hole, USA.
^8^  University of Arizona, Department of Radiation Oncology/Banner University Medical Centre—Medical Physicist, Phoenix, USA.
^9^  University of Arizona, College of Optical Sciences—PhD, Phoenix, USA.
^10^  DABR, Phoenix, USA.

**Background/rationale or objectives/purpose**

Pediatric cancer treatment can be traumatic, especially in radiation therapy where children are isolated from their caregiver while in the radiation vault. One key strategy to reduce stress, anxiety and anesthesia is the utilization of video-based distraction therapy. Until recently, video distraction has been an underutilized tool due to technical challenges of providing a radiation compatible video system. With modern advances available through RadFlix, a new non-profit entity, this limitation is now removed.

With the growth of psychosocial care and research for improved practices for children with cancer, it is vital to explore the positive effects of video-based distraction in patients receiving radiation and how associations between this type of social support and quality of life are correlated. We plan to explore the patient-centered view of this pediatric population and their families.

**Methodology or Methods**

A qualitative survey of children’s experiences will be examined when utilizing video-based distraction while receiving radiation treatment. Interviews with patients, families and practitioners will be completed, examining participants’ experiences of being supported in this capacity.

This study will aim to characterize how the utilization of video distraction without positional limitations can improve patient experience and reduce overall patient stress, anxiety and anesthesia use.

**Impact on practice or results**

By elevating patient experience through the utilization of video-based distraction and ensuring that patient voices are centered in cancer care, improving quality of life, we are hypothesizing to see a reduction in stress, anxiety and anesthesia use.

**Discussion or Conclusions**

The utilization of video distraction through RadFlix can become an integral part of cancer care treatment in pediatrics.

**143**

### 18.5. Cross-Sectional Assessment of Sexual Health in Patients with a Vagina Receiving Radiotherapy

Kaviya Devaraja ^1,2^, Abha A. Gupta ^2,3^ and Jennifer Croke ^4^.

^1^  Institute of Medical Science, University of Toronto, Toronto, Canada.
^2^  Adolescent and Young Adult Program, Department of Supportive Care, Princess Margaret Cancer Center, University of Toronto, Toronto, Canada.
^3^  Division of Medical Oncology and Hematology, Princess Margaret Cancer Center, University of Toronto, Toronto, Canada.
^4^  Radiation Medicine Program, Princess Margaret Cancer Center, University Health Network, Toronto, Canada.

**Background/rationale or objectives/purpose**

Radiotherapy (RT) plays an important role in pelvic malignancies for patients with a vagina. However, associated sexual and vaginal side-effects can impact a patient’s mental health, relationships and quality of life. Sexual health assessments, especially for adolescents and young adults (AYAs) (aged 15–39) with a vagina, are essential as they are at a unique life stage, navigating fertility preservation and developmental stages. This study explores the experiences and challenges faced by AYAs with a vagina receiving RT to inform life-stage appropriate support and resources.

**Methodology or Methods**

This simultaneous mixed-methods study involves a cross-sectional survey of AYA patients with a vagina who received RT at the Princess Margaret Cancer Center to assess socio-demographics, sexual health experience and satisfaction with sexual health education and management. Additionally, one-on-one semi-structured interviews are conducted to explore sexual health experiences, challenges and needs. Survey responses will be summarized using descriptive statistics, while a thematic analysis of interviews will be performed with NVivo 10.

**Impact on practice or results**

The results will unveil patterns, recurring themes and meaningful insights within the narratives of AYA patients with a vagina who have undergone pelvic radiotherapy, shedding light on their unique experiences, challenges and needs related to sexual health.

**Discussion or Conclusions**

These findings will provide valuable insights into the sexual health experiences of AYAs with a vagina after RT, enabling the identification of care gaps and patient needs. These insights will inform the development of targeted supportive care tools.

**158**

### 18.6. Taking a Pulse: Insights into the Canadian Association of Psychosocial Oncology’s Value and Priorities

Sydney Wasserman ^1^, Sitara Sharma ^2^ and Doris Howell ^3^.

^1^  McGill University, Ingram School of Nursing, Montreal, Canada.
^2^  University of Ottawa, School of Human Kinetics, Ottawa, Canada.
^3^  University Health Network, Princess Margaret Cancer Research Center and Lawrence S. Bloomberg Faculty of Nursing, University of Toronto, Toronto, Canada.

**Background/rationale or objectives/purpose**

The *Canadian Association of Psychosocial Oncology* (CAPO) formulated a Strategic Plan for 2022–2027, outlining its vision and mission based on member priorities. Key goals include (1) leadership/advocacy, (2) innovation/education, (3) clinical/community care and (4) research/knowledge translation. This descriptive study aimed to “take a pulse” of CAPO to assess its alignment with this plan and deepen the future direction.

**Methodology or Methods**

In October 2023, CAPO members were solicited via targeted emails to complete an online survey evaluating their perspectives on organizational value and priorities. Descriptive statistics were used to describe the sample and analyze close-ended questions; abductive content analysis was performed to interpret open-ended responses.

**Impact on practice or results**

Of the 58 survey respondents, the majority identified as female (69%), White (70.7%) and were based in Central/West Canada (67.2%). The participants were generally engaged in clinical (53.4%) or academic roles (20.7%), with less student (10.3%) and patient/caregiver partner (6.9%) representation. Most (90%) perceived value in their CAPO membership, citing benefits like access to “up-to-date information” and “high-quality resources” on best practices, alongside networking and educational opportunities (e.g., annual conferences, research webinars). Nevertheless, participants emphasized a need for CAPO to prioritize accessibility (e.g., financial aid and hybrid event options) and engagement (e.g., tailored and digestible content, online communities, stakeholder and diverse group involvement and collaboration/mentorship) moving forward.

**Discussion or Conclusions**

The findings indicate some alignment between CAPO’s Strategic Plan and recent member feedback, while also highlighting areas for improvement. Insights may inform the development of more inclusive and relevant resources to better support those dedicated to improving psychosocial oncology care across Canada.

Author NameProgram Codes *Adham, Heide27, 80Affoo, Rebecca155Agnew, Kelly116Aguiar, Stefan
154
Ahmed, Saima
69
Albizu-Jacob, Alexandra54Alexander, Sarah50, 114Ali, H M Ashraf
40
Angka, Leonard32Anstruther, Se’era34, 74Arab, Marianne18, 19Askari, Zahra147Attieh, Samar8, 32, 44Audoin, Michelle78Auer, Rebecca32Avery, Jonathan48, 119, 112, 121Bachini, Melinda32Bansal, Mannat91, 96Barbeau, Anne154Barbera, Lisa34Bates, Alan45Baydoun, Mohamad
25
Bays, Suzanne81Beattie, Sara35, 106, 130Ben, Smith117Bender, Jackie83, 130Benea, Aronela93Benites, Andrea Carolina10Bennett, Julie48Bergeron, Élodie55, 150Bhullar, Imrose91Bilash, Tristan37Biondo, Patricia60, 99Bouchard, Isabelle68Bourdeau, Camille
47
Boychuk, April35Brisson, Jeff75Brittany, Mutsaers117Brosseau, Danielle
31
Brown, Tyler
146
Brownstone, David50Brunet, Alain12Bryant-Lukosius, Denise103Buckle, Jennifer
148
Buick, Catriona71Burrows, Kathryn74Butler, Chelsea
147
Campbell, Kristin83Campbell, Tavis132Cannon, Jocelyn85Capozzi, Lauren C.96Carlson, Linda43, 92, 123, 129, 130Carson, Kimberly30Caru, Maxime68Cassidy, Christine9Catsburg, Jennifer112Chamorro-Viña, Carolina115Chan, Kelvin88Charbonneau, Andy47Cheung, Winson88Cho, Sara22, 82Chomistek, Tessa
136
Chu, Alanna126, 130Conradi, Hanna43Conrod, Randy77Corbin Dwyer, Sonya148Cory, Shelly109Costa, Daniel117, 127Cote, Rebecca50, 112Courville, Julien
111
Craig, Lise77Cripps, Hannah86, 100, 115Croke, Jennifer142, 143Crosby, Kim59, 95, 97Culos Reed, S. Nicole38, 49, 78, 85, 86, 90, 91, 96, 100, 115Cumming, Ceinwen59, 95Curnier, Daniel68Cuthbert, Colleen38, 41, 88D’Agostino, Norma113D’Mello, Brooke
39
D’Souza, Melba
4
D’Souza, Violet155, 156, 157Daga, Guilherme62Daly, Gordon26Daniels, Maureen48Danyluk, Jessica85Darrouj, Silva
103
Daun, Julia49, 85, 96de Groot, Janet92, 128, 138, 139, 140, 141de Guzman Wilding, Marie62, 92de la Sablonnière, Roxane47Dean, Michelle Lianne30Deckert, Eleanor
36
DeIure, Andrea34, 74Deleemans, Julie
43
Desjardins, Leandra55, 150Devaraja, Kaviya48, 142, 143Dhruva, Tana90, 96Diedrich, Karine
76
Dolgoy, Naomi122Domene, José31Donnan, Jennifer105, 107Donovan, Kristine A.
54
Dow, Megan
96
Dowd, A. Justine
49
Dowling, Gavin26Dreger, Julianna38, 100Drummond, Rachelle28, 52, 63, 98, 137Duong, Jenny27, 80, 82, 87Durante, Salvatore B.59Durante, Stephanie
40
Duval, Michel11Easaw, Jacob C.96Easley, Julie13Edelstein, Kim48El Houjairy, Intisar
133
Ellis, Peter103Elman, Joel159Emenji Asaba, Florence
21
Evans, Deb103Farrer Rogers, Christie41Fay-McClymont, Taryn101Feldstain, Andrea92, 130, 138, 140, 141Fergus, Karen31, 71, 73, 120Finlay, Jenise
88
Fisher, Sara115Fitch, Lauren M.17Fitch, Margaret13, 14, 17Flores Pereira, Luana10, 93Foran, Laura
110
Forbes, Caitlin27, 52, 63, 80, 82, 87, 101, 133, 136, 137Forster, Victoria82Fortin, Justine
12
Francis, George85, 96Freeman, Laurie77Frisk, Nadine37Galarneau, Luc116Galica, Jacqueline77, 81Gamboa, Jessame128, 138Garland, Allan134, 135Garland, Sheila23, 46, 79, 105, 107, 130Gascon, Bryan
159
Gendron Granger, Nadège
55
George, Brianna
79
George, Megan
89
Gibson, Amanda30Gibson-Haerle, Janelle75, 82Giguère, Lauriane75, 117, 127Giles, Jennifer87Gill, Param121Glass, Karen120Graham, Robin154Greeley, Krista23, 46Greenland, Jonathan83Grendarova, Petra122Gross, Erin71Guidelines Panel Members, SIO-ASCO Anxiety-Depression123Guilcher, Gregory101Gulee, John Ubale6Gupta, Abha48, 112, 142, 143Hachey, Shauna155Hagopian, Emma154Hannon, Breffni39, 151Hanson, Kaitlyn134, 135Harris, Cheryl117, 126, 127, 130Harris, Nick105, 107Hatala, Andrew134, 135Henry, Brianna28, 52, 63, 82, 137Heykoop, Cheryl119, 121Hill, Leslie18, 19Hill, Tiffany119, 121Hinse, Treena59, 95, 97Ho, Cheryl45Hollingsworth, Sarah62Holmer, Pauline52Holmes-Redman, Paula139Holt, Dr Douglas118Holy, Celeste75Hou, Sharon28, 63, 137Houle, Sandra20House, Chloe
105
Howell, Doris30, 158Howes, Janice18, 19Humphris, Gerald117, 127Huta, Veronika126Ianakieva, Iana71, 73Jean Julien, Krisen111Jibb, Lindsay114Johnson Agbo, Joy21Jones, Jennifer83, 113, 130Joy, Anil Abraham78Judd, Susie116Kaluznick, Georgia115Kansiime, Rachel
2
Kanter, Cheryl48Karim, Safiya129Karkada, Subrahmanya
4
Kashuba, Louise60, 99Kearley, Allison
9
Kearns, Emma117Keats, Melanie9, 38Kenny, Sarah J.90Khan, Omar30Kildea, John116Killoran, Sheila
99
Kim, Yoojung53, 69Kizito, Simon2Knaapen, Loes116Kogan, Cary117, 127Korenblum, Chana50, 112Krull, Kevin101Labelle, Laura
62
Labonté, Nathalie55Ladhani, Noor Niyar120Lafay-Cousin, Lucie101Lafontaine, Christine32Laizner, Andréa Maria
116
Lambert, Sylvie104, 111Langelier, David83, 85, 90Langleu, Jodi9, 38Lau, Emily
151
Lau, Sharlane
113
Laverdière, Caroline47, 68Lebel, Sophie75, 77, 113, 117, 126, 127, 130Leduc, Shania
30
Lee, Rachel23, 46, 105, 107Leia, Madison
41
Leung, Bonnie45Leung, Shuk On Annie111Leung, Yvonne159Levesque, Ariane
68
Lheureux, Stephanie89Li, Athena39Li, Madeline113, 152, 154, 159Link, Claire34, 74Lockwood, Gina14, 17Loecher, Nele54Logie, Dr Natalie
118
Loiselle, Carmen8, 15, 17, 32, 44, 53, 69Longo, Christopher J.17Look Hong, Nicole88Lopez, Christian
83
Louis-Delsoin, Wendy55Lowe, Sonya
122
Ma, Kenneth39Macedo, Alyssa159MacLeod, Deborah125MacNeil, Mary9Mah, Kenneth151Mahar, Alyson134, 135Maheu, Christine77, 113, 130Male, Dana
35
Malfitano, Carmine89, 110, 114Mann, Jasmine157Manning, Miranda60Marcil, Valérie68Marin, Marie-France12Marquis, Marc-Antoine11Mason, Warren48Matthew, Andrew125Mazzurco, Olivia154McCarthy, Joy23McDonald-Wirasinghe, Kaelyn27, 80, 82McDonough, Meghan H.91, 96McLaughlin, Emma86, 100, 115McLean, Michelle95, 97McMillan, Kimberly75, 126McNeely, Margaret38, 78, 96, 122McPherson, Christine126Meloche, Caroline68Mendonca, Kyle28, 137Mestari, Zakaria150Mi, Ellen99Michaud, Nicola95, 97Michon, Bruno11Miljanovski, Melissa39, 151Miller, Brian118Mittal, Ridhi116Mksyartinian, Patil147Monarque, Marika55Moore, Sara126Moorman, Jessie35Moraru, Camelia67Mosher, Pamela113Mukherjee, Som103Murray, Maril60, 99Mutsaers, Brittany127Mutto, Milton2Nairy, Ashwin
4
Nanos, Stephanie
114
Naylor, Desiree
145
Needham, Judy81Neil-Sztramko, Sarah83Nette, Hannah
4
Newton, Lorelei17Ng, Raymond45Nguyen, Patricia93Nguyen, Tina
129
Nicoll, Irene14, 17Nissim, Rinat77, 113, 126Nneke, Kelechi Uzochukwu6Noble, Justin81Nordstokke, David27Norton, Lucas71, 73Nunez, John-Jose
45
Oberoi, Sapna134, 135Ogunlowo, Oluwapelumi26Okonkwo, Ada119, 121Olivier-D’Avignon, Marianne11Olson, David71Omobhude, Oserekpamen Favour52Onwukeme, Ifeyinwa Carol
6
Onwukeme, Joseph Chimezie6Oprea, Ruxandra Mara67Orem, Jackson2O’Brien, Suzanne69Panjwani, Aliza113, 154Parkinson, Merry76Patten, Scott140, 141Peach, Payton107Peacock, Stuart88Pelletier, Wendy75Penney, Bridget115Penney, Mira115Peres, Dalia120Perez, Samara111Perry, Sarah85Pete, Jacob156Petrella, Jill18Philp, Lauren89Pink, Jennifer35Pitch, Natalie112Pituskin, Edith78Pond, Greg103Popa, Isai67Provost, Carole47, 55Qi, Siwei34Quinn-Nilas, Christopher79Raha, Devbani18Rahamatullah, Iqra63Ramasamy, Vinesha135Rash, Joshua23, 46, 79Rayar, Meera81Reiman, Tony83Reynolds, Kathleen87Rivard, Mélina
150
Roberts, Araby52Robichaud, Lye-Ann
11
Robinson, John125Rodin, Danielle88Rodin, Gary39, 89, 110, 114, 151, 152, 154, 159Rogers, Lillian121Roldan Urgoiti, Gloria96Rondeau, Émélie47, 68Rosberger, Zeev55Rosenal, Tom139Roy, Heather81, 118Rudd, Émilie12Russell, K Brooke
52
Rutkowski, Nicole
106
Rydall, Anne154Sanders, Justin146Santa Mina, Daniel38Savard, Josée77, 79Savas, Sevtap37, 44, 81Schulte, Fiona27, 28, 35, 52, 63, 75, 80, 82, 86, 87, 98, 101, 106, 133, 135, 136, 137Scott, Ian134, 135Seal, Melanie23Sears, Carly125, 128, 138, 140, 141See, Kaitlin41Sellar, Christopher122Shah, Fatima I.124Shallwani, Shirin M.
78
Shantz, Mary-Ann60Shapiro, Gilla152, 154Sharma, Sitara
158
Shenthil, Suchithra
127
Sibley, Daniel
38
Silveira, Kristen138, 140, 141Sim, Jenna90Simard, Sebastien117, 127Simon, Jessica140, 141Simoneau, Rick69Sinnett, Daniel68Sirois-Leclerc, Heloise75Sitter, Kathleen128Smith, Ben127Smith, Marlie113Smrke, Alannah119St. John, Ph.D., Laura86Stevenson, Ben71, 73Stirling, Morgan134Stokoe, Mehak27, 52, 80, 82, 87, 101, 136Strohschein, Fay17, 138, 140, 141Strother, Douglas101Strudwick, Gillian83Stuckert, Nola
96
Sultan, Serge11, 47, 55, 68Sussman, Jonathan103Taccone, Michael82Takacs, Judit81Tayabee, Zeba135Temple-Oberle, Claire22Thibodeau, Kimberley37, 44Thomas, Roanne
20
Thompson, Lora M.A.54Thompson, Marney
109
Thurston, Chantale37, 106Tippin, Greg103Toma, Camelia67Tomfohr-Madsen, Lianne52Townsley, Carol93Tramblay, Julie150Trinh, Linda38Tromburg, Courtney101, 136Trudel-Fitzgerald, Claudia12Tsang, Derek S.48Tsimicalis, Argerie55Tulk, Joshua46, 79, 132Turin, Tanvir C.91Turner, Jill31Tutelman, Perri35, 75, 106Tyo-Gomez, Mathias11Ubeira Amaral, Eugenia
152
Udrea, Iulia
67
Urquhart, Robin9van Velzen, Roxanne134Vanstone, Ruth
120
Verhesen, Tessa52Vu, Linh
26
Wagoner, Chad38, 85, 90Walker, Lauren124, 125Wang, Yuqi155, 156Warner, Ellen120Warner, Grace9Wasserman, Sydney104, 158Wassersug, Richard125Watson, Linda34, 74West, Christina144White, Emily
107
Wibowo, Erik125Wich, Laura115Wilcox, Gabrielle27Williams, Tristan116Williamson, Tanya96Wilson, Kristin134Winther Klippenstein, Andrea
144
Wolfe, Jennifer121Wong, Rebecca
39
Wong, Veronica103Wood, Don41, 81Worley, Josh
40
Wright, Holly28, 52, 137Wurz, Amanda86, 100, 115Yeates, Keith101Yen, Jonathan103Zari Rukundo, Godfrey2Zhang, Karen
103
Zheng, Yuanjie
60
Zimmermann, Camilla39, 151Zohair, Mirha134, 135Zwaal, Caroline77Zwicker, Hailey101, 136* if no program code was given for a submission, then this will show the submission serial number.

